# Carbazole‐Based Tetrapodal Anchor Groups for Gold Surfaces: Synthesis and Conductance Properties

**DOI:** 10.1002/anie.201911652

**Published:** 2019-11-27

**Authors:** Luke J. O'Driscoll, Xintai Wang, Michael Jay, Andrei S. Batsanov, Hatef Sadeghi, Colin J. Lambert, Benjamin J. Robinson, Martin R. Bryce

**Affiliations:** ^1^ Department of Chemistry Durham University, Lower Mountjoy Stockton Road Durham DH1 3LE UK; ^2^ Physics Department Lancaster University Lancaster LA1 4YB UK; ^3^ School of Engineering University of Warwick Coventry CV4 7AL UK

**Keywords:** DFT calculations, molecular electronics, monolayers, oligo(phenylene-ethynylene), scanning probe microscopy

## Abstract

As the field of molecular‐scale electronics matures and the prospect of devices incorporating molecular wires becomes more feasible, it is necessary to progress from the simple anchor groups used in fundamental conductance studies to more elaborate anchors designed with device stability in mind. This study presents a series of oligo(phenylene‐ethynylene) wires with one tetrapodal anchor and a phenyl or pyridyl head group. The new anchors are designed to bind strongly to gold surfaces without disrupting the conductance pathway of the wires. Conductive probe atomic force microscopy (cAFM) was used to determine the conductance of self‐assembled monolayers (SAMs) of the wires in Au–SAM–Pt and Au–SAM–graphene junctions, from which the conductance per molecule was derived. For tolane‐type wires, mean conductances per molecule of up to 10^−4.37^ G_0_ (Pt) and 10^−3.78^ G_0_ (graphene) were measured, despite limited electronic coupling to the Au electrode, demonstrating the potential of this approach. Computational studies of the surface binding geometry and transport properties rationalise and support the experimental results.

## Graphical abstract

**Four‐legged friends**: Self‐assembled monolayers of molecular wires with new anchoring units containing four thiomethyl groups were formed on gold and their conductance was measured. The tetrapodal anchors provide effective binding but contribute minimally to the conductance pathway of the wires. Surface binding and electronic coupling can therefore be considered independently.

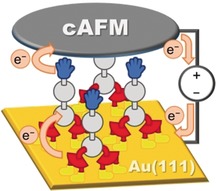

## Introduction

Anchor groups fulfil a critical role in materials designed to assemble on surfaces.^1^ In the field of molecular‐scale electronics, they serve as contact points between conductive molecules and electrode surfaces. The strength of binding interactions and extent of electronic coupling between an anchor group and an electrode are important factors when designing conductive organic materials.^2^ For fundamental studies, an anchor able to form a conductive junction with a lifetime longer than a molecular conductance experiment is adequate. However, when working towards the goal of functional devices based on conductive molecules,2a such as thermoelectric generators^3^ and Peltier coolers,^4^ strengthening the interactions between these molecules and electrode surfaces is necessary to achieve effective device lifetime and performance. Improved anchor groups can also result in more ordered surface assemblies.

Thiols, thioethers, and pyridines, amongst others, are common anchor groups in fundamental molecular conductance studies using gold surfaces.^2^ Recently, several new anchor groups have been designed to exhibit increased affinity for gold surfaces, with various applications.1a–1c, 5 Some examples pertinent to molecular electronics are shown in Figure S1.29 in the Supporting Information (SI). In many cases, these anchors are tripodal with three conventional anchor groups working together to achieve enhanced surface binding, akin to the chelate effect. Surface–π interactions can contribute additionally to this binding. Tripodal anchors often incorporate an sp^3^‐hybridised carbon centre to induce the desired geometry. High molecular conductance is associated with conjugation,2a meaning that sp^3^‐hybridisation is considered undesirable in conductive materials, although a study of a pyridine‐anchored tripod describes contrasting results.5a Positioning the sp^3^‐centre outside the conductance pathway of the molecule can avoid any impact on conductance.5b An additional feature of larger anchors of this type is that they increase the spacing between conductive molecular backbones and thereby prevent intermolecular interactions.5c

In this work, we present a new tetrapodal anchoring motif designed to bind strongly to gold surfaces and form ordered self‐assembled monolayers (SAMs) with well‐spaced conductive backbones. Inspired by tripodal systems,5a, 5b, 5d–5i multiple anchoring points are incorporated into our design to ensure efficient surface binding. In contrast to many current approaches, we aimed to separate these additional anchoring points from the conductive backbone of the molecule and avoid sp^3^‐hybridisation in the conductive pathway. We note that while this study focuses on the molecular electronics applications of our new anchoring motif, it may also prove adaptable as a useful scaffold for other applications, for example, gold surface‐assemblies of optoelectronic materials,^6^ switches5d, 7 or polymerisation initiators.^8^

## Results and Discussion

Taking the ubiquitous oligo(phenylene‐ethynylene) (OPE) backbone as a starting point, we selected the *meta*‐positions with regard to the conductive backbone as an ideal point to incorporate additional anchoring functionality. Conductance through a *meta*‐conjugated pathway is considerably lower than the favoured *para*‐conjugated pathway due to destructive quantum interference (QI) effects.2b, 9 Therefore, any additional *meta*‐functionalisation should contribute minimally to the conductance of the molecule. Inspired in part by the spirobifluorene tripods reported by the Mayor group,5b, 5e carbazole was selected as the basis of the additional anchors. This choice further disfavours any conductance through the poorly conducting *meta*‐positions as steric factors should induce a significant twist between the backbone and carbazole π‐systems, reducing conjugation between them. Furthermore, this twist should help to direct the conjugated backbone away from the surface. In addition to any interactions between its π‐electrons and the surface, carbazole can be readily functionalised in the 3‐ and 6‐positions, allowing convenient incorporation of additional binding functionality.

**Figure 1:**
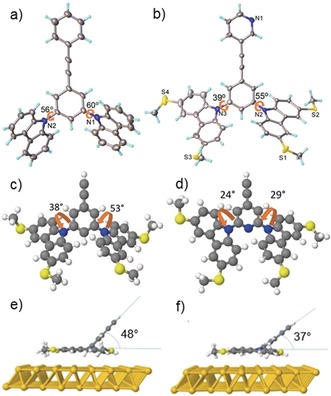
a) X‐ray structure of model compound **1**; b) X‐ray structure of **B*m*P**; c) Gas phase DFT relaxed model system with benzene base; d) Gas phase DFT relaxed model system with pyridine base; e) model system with benzene base relaxed on Au(111) surface; f) model system with pyridine base relaxed on Au(111) surface.

**Scheme 1:**
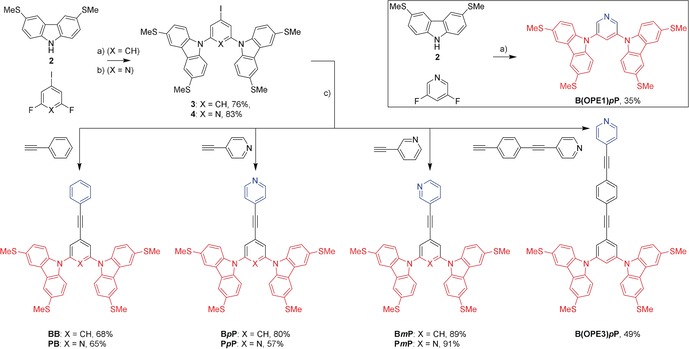
Synthesis of tetrapodal molecular wires. Reagents and conditions: a) Cs_2_CO_3_, DMF, 100 °C, 17 h; b) K_2_CO_3_, DMSO, 70 °C, 2 h; c) CuI, Pd(PPh_3_)_2_Cl_2_, THF, DIPEA, RT, 80–120 min. For the tetrapodal molecular wires, the anchoring unit is coloured red, and the head unit blue (note that for **B(OPE1)*p*P** the head unit pyridine ring also forms part of the anchor group).

To test this design the model compound **1** was initially prepared (see SI, Section 1.6). The single crystal X‐ray structure (Figure 1 a) confirmed the expected twist between the backbone and the carbazole units. To investigate the anchoring properties of the unit a series of OPE2 type molecules (where 2 refers to the number of aryl rings in the backbone) were then synthesised, each with one tetrapodal unit (anchor) and one simple terminal aryl group (head). Two anchors were proposed, with either a central benzene (X=CH) or pyridine (X=N) ring, in each case functionalised with two 3,6‐bis(methylthio)carbazole units, that is, four thiomethyl groups per anchoring unit. The thiomethyl anchor groups were preferred to acetyl‐protected thiols to ensure compatibility with the synthetic route and to reduce synthetic complexity. The use of multiple thioethers has been reported to result in strong anchoring to a gold surface, where each thioether makes a contribution comparable to that expected from a thiol.^10^ For the benzene systems, anchoring is expected from only the functionalised carbazole subunits, whereas the pyridyl nitrogen may be able to interact additionally with the gold surface. Three head groups were investigated, namely benzene, *p*‐pyridine and *m*‐pyridine. The benzene head group has no defined anchor point and should therefore interact poorly with metallic electrodes. Benzene should, however, be able to interact with graphitic surfaces through π–π overlap at appropriate contact angles. Both *p*‐ and *m*‐pyridyl head groups should bind to metallic electrodes through the nitrogen lone pair, with the *para* derivative expected to show a higher molecular conductance than the *meta* derivative due to QI effects. The structures of these species are shown in Scheme 1. Our naming convention is to indicate the anchor unit (**B** or **P**) followed by the head unit (**B**, ***p*** **P** or ***m*** **P**), for example, **B*p*P** is the compound with a benzene‐based anchor and a *para*‐pyridyl head.

A key building block is the thiomethyl‐substituted carbazole derivative **2**. Although it has been previously reported, limited experimental details and characterisation data are available.^11^ We developed an alternative synthesis using 3,6‐diiodocarbazole^12^ as the precursor. After TBDMS‐protection of the *N*‐position, thiomethyl substituents were introduced via lithiation followed by treatment with dimethyl disulfide, although optimisation was required to minimise by‐product formation and achieve practical yields (see SI, Section 1.7). Straightforward deprotection of the TBDMS group using TBAF afforded **2**. By using nucleophilic aromatic substitution (S_N_Ar) reactions it was then possible to prepare the benzene‐ and pyridine‐based scaffolds **3** and **4** (Scheme 1). Both are functionalised with an iodide group as a convenient synthetic handle for subsequent synthetic transformations. This is an advantage of the S_N_Ar route; alternatives based on metal‐catalysed coupling reactions would have a higher potential for by‐product formation and would either rely on statistical reactions or utilise a less‐reactive bromide group as the resulting synthetic handle. A disadvantage of the S_N_Ar approach is that it is poorly compatible with central rings bearing electron‐donating substituents, as these disfavour S_N_Ar reactions.

With the iodide species in hand, the targeted library of asymmetric molecular wires was prepared using Sonogashira protocols (Scheme 1). For comparison in the conductance studies, a series of analogous OPE2 derivatives were also prepared with a simple (protected) thiol anchor (denoted with **S** in our naming convention) and the three head groups used in the tetrapodal series (See SI, Section 1.8). To probe the effect of changes to the length of the conductive backbone, the shorter (**B(OPE1)*p*P**) and longer (**B(OPE3)*p*P**) analogues of **B*p*P** (Scheme 1) were also prepared. They differ by the effective subtraction or addition of a phenylethynyl unit into the conductive OPE2 backbone.

Single crystal X‐ray structures were obtained for model compound **1** and for **B*m*P**. For **1**, the torsion angles between the central benzene ring and the two carbazole units are 56° and 60° (Figure 1 a), whereas for **B*m*P**, which showed noticeable structural disorder in the positions of the pyridyl‐nitrogen and the thiomethyl groups (see SI, Section 2.1), these angles are 55° and 39° (Figure 1 b). Using the SIESTA package,^13^ density functional theory (DFT) simulations (see SI, Section 3.1) of model, alkyne‐terminated systems in the gas phase gave corresponding angles of 38° and 53° for a benzene (X=CH) base (Figure 1 c) and 24° and 29° for a pyridine (X=N) base (Figure 1 d). The reduced torsion in the latter can be attributed to reduced steric hindrance. H‐bonding interactions may also contribute (see SI, Section 2.2). In all cases, to differing extents, the designed twist is observed between the carbazole and OPE π‐systems, which should reduce the influence of the carbazole units on the conductive backbone. DFT was also used to simulate the assembly of these model systems on Au(111) surfaces. As shown in Figures 1 e and 1 f, the two carbazole units splay out to lie essentially flat on the gold surface (indicative of favourable surface‐π interactions), with the OPE backbone protruding at an angle. The angle between the gold surface and the conductive backbone is 48° for a benzene base and 37° for a pyridine base. Therefore, little conjugation is expected between the conductive backbone and the carbazole units in this conformation.

The binding energies of these conformers to a Au(111) surface, calculated using the counterpoise correction method,^14^ are −1.86 eV (X=CH) and −1.68 eV (X=N). These values suggest that the pyridine nitrogen atom does not enhance surface binding (at least in this conformation), and in fact results in slightly weaker surface interactions than the benzene analogue. The binding energies of some simple, conventional anchors were reported previously using the counterpoise method.^15^ Comparison with these values shows that both tetrapodal anchors have considerably higher binding energies than amine (−0.30 eV), nitrile (−0.41 eV), dihydrobenzothiophene (−0.41 eV) and pyridine (−0.50 eV) anchors, and have enhanced binding compared to thiols (−1.51 eV). A significant increase in binding energy is also observed in comparison to phosphine‐based tripodal anchors with three thiomethyl anchoring groups (ca. −1 eV).5f The adsorption energies of triptycene tripods with three thiol anchors, which account for loss of hydrogen upon binding to a gold surface, were found to be −1.62 eV (for less flexible aryl thiols) and −2.67 eV (for more flexible benzylic thiols).5g The tetrapodal anchors perform comparably to the former case despite using thiomethyl anchors rather than thiols. Although it binds strongly, the latter triptycene design includes sp^3^‐carbons so is likely poorly suited to molecular electronics applications.

The properties of SAMs of the tetrapodal molecules on Au(111) surfaces were assessed using a range of techniques. Details of the preparation of SAMs, characterisation methods and associated images can be found in the SI (Section 2.3). Atomic force microscope (AFM) imaging showed that the SAMs were densely packed films with uniform structures in the range of 0.5–1 nm. The thickness of the SAMs was determined using a nano‐scratching method.^16^ The averaged film thickness for the OPE2 molecules was in the range of 0.6–0.65 nm, and for OPE3 derivative **B(OPE3)*p*P** it was 0.9±0.04 nm. These values correspond to tilting angles of ca. 35° between the OPE backbones and the substrate surface, in reasonable agreement with the DFT calculations (Figures 1 e,f). SAMs of the model compound **1** could not be prepared on gold, showing that the thiomethyl groups play a critical role in surface assembly. DFT simulations confirm this, showing stepwise increases in binding energy as thiomethyl groups are sequentially added to the carbazole‐based anchoring motif (see SI, Section 3.1 for further discussion).

The density of molecules on the gold substrate was determined using reductive desorption and quartz crystal microbalance (QCM) measurements. Reductive desorption allowed the molecular area to be calculated based on the charge density of the desorption peak, under the assumption that four electrons correspond to desorption of a single molecule (i.e. one electron per thiomethyl anchor group). A second cycle of desorption confirmed that all molecules were desorbed from the surface in the first sweep (SI, Figure S2.07). **BB** and **PB** were investigated as representative molecules and had estimated molecular occupation areas of 265 Å^2^ and 300 Å^2^, respectively. This agrees well with a DFT‐estimated molecular footprint of 265 Å^2^ for either benzene‐ or pyridine‐based tetrapods (see SI, Section 3.1). The desorption potential of benzene‐based material **BB** was −0.57 V vs. SCE, whereas for **PB** the desorption potential was −0.67 V vs. SCE. In this case, the pyridine‐based anchoring unit appears to provide a small additional anchoring effect compared to the benzene‐based analogue. Although this appears to disagree with the DFT‐calculated binding energies stated above, we note that the methods may not be directly comparable as reductive desorption relates to electrochemical stability, whereas the binding energies relate to adsorption. QCM measurements (see SI, Section 2.5) gave estimated molecular areas around 30 % lower than those derived from reductive desorption. AFM imaging of the QCM substrate (SI, Figure S2.1) showed that the surface was much rougher than those used in the other measurements and was therefore likely to result in underestimation of molecular areas. As the substrate used for reductive desorption is comparable to those used in conductance studies, we believe this is the more reliable method for determining molecular area in this case.

**Table 1:** Summary of values derived from reductive desorption and quartz crystal microbalance studies.

Molecule	Desorption potential/ V vs. SCE	Molecular area/ Å^2^ (reductive desorption)	Molecular area/ Å^2^ (QCM)
**BB**	−0.57	265	187
**PB**	−0.67	301	220

Investigations of the conductance of molecules with multiple binding sites using single‐molecule junction techniques can be challenging, as various junction configurations are possible.5h This generally results in complex conductance data from which it can be difficult to determine the contributions of different configurations. To avoid this complication, the conductance properties of the tetrapodal wires were investigated using conductive probe AFM (cAFM). The analysis of SAMs of the tetrapods indicated that the molecules were binding to the gold surface with all four thiomethyl groups in the designed manner. Therefore, it was expected that the protruding head groups would be contacted with the cAFM probe and afford the desired Au‐anchor‐head‐probe junction configuration. A further advantage of cAFM is that measuring the conductance of SAMs should be more representative of potential large‐area devices than single‐molecule methods.

**Figure 2:**
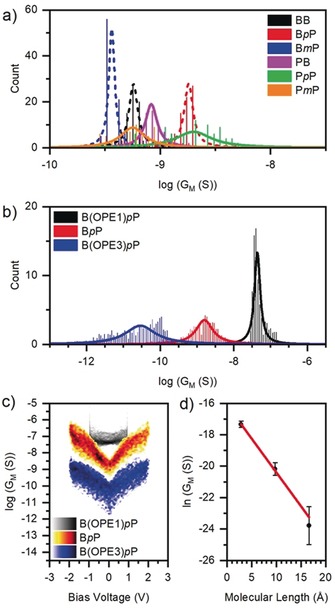
a) Histograms of conductance per molecule (*G*_M_) for Au‐SAM‐Pt junctions containing tetrapodal OPE2 molecular wires at low bias voltage (−0.1 V to 0.1 V), fit curves are a guide for the eye; b) Histograms of *G*_M_ values for Au‐SAM‐Pt junctions containing tetrapodal molecular wires of different lengths at low bias voltage (−0.1 V to 0.1 V), fit curves are a guide for the eye; c) Differential conductance (per molecule) versus voltage for Au‐SAM‐Pt junctions containing tetrapodal molecular wires of different lengths; d) Plot of ln (*G*_M_) vs. molecular length which allows the tunnelling decay factor, *β*, to be calculated.

Electrical maps obtained via cAFM revealed that, in general, the SAMs showed very good electrical uniformity. However, the electrical uniformity of SAMs of tetrapods with pyridine bases was poorer than that of SAMs of benzene‐based analogues prepared under the same conditions: there are clearly some uncovered regions in the electrical map in the pyridine case (cf. **P*p*P** and **BB**, SI, Figure S2.11). The electrical conductance of the SAMs was determined through cAFM IV measurements using both Pt‐ and graphene‐coated probes (See SI, Section 2.6). After gently approaching the surface with a new cAFM probe, the contact force was set to 2 nN and the bias voltage was swept (typically from −1 V to +1 V) at at least 20 randomly selected locations on the sample surface (Figures 2 c, S2.12, S2.13 and S2.15). At least 3 IV sweeps were conducted at each location and used to calculate the differential conductance of the junction (*G*_J_). The number of molecular junctions formed between the probe and the substrate was estimated using the Hertz model^17^ (see SI, Section 2.6). This allowed the conductance contribution of a single molecule (*G*_M_) to be calculated for each IV curve. At least 60 IV curves were measured for each molecule, giving a distribution of *G*_M_ values (Figures 2 a,b, S2.14 and S2.16). The mean *G*_M_ values for each molecule are listed in Table 2.

**Table 2:** Summary of mean single‐molecule conductance (*G*_M_) values derived from cAFM measurements (uncertainties are standard deviations). Structures of thiol‐anchored reference compounds **SB**, **S*p*P** and **S*m*P** can be found in Scheme S3 in the SI.

	Pt probe		Graphene probe	
Molecule	*G*_M_/ nS	log(*G*_M_/*G*_0_)	*G*_M_/ nS	log(*G*_M_/*G*_0_)
**BB**	0.89(±0.07)	−4.94	4.5(±0.38)	−4.24
**PB**	1.36(±0.14)	−4.76	6.5(±0.89)	−4.08
**B*p*P**	2.8(±0.15)	−4.44	8.6(±0.94)	−3.95
**P*p*P**	3.3(±0.36)	−4.37	12.9(±1.02)	−3.78
**B*m*P**	0.49(±0.03)	−5.20	2.5(±0.22)	−4.49
**P*m*P**	0.97(±0.10)	−4.90	4.1(±0.41)	−4.28
**B(OPE1)*p*P**	29.5(±2.16)	−3.42	–	–
**B(OPE3)*p*P**	0.047(±0.01)	−6.22	–	–
**SB**	6.4(±0.58)	−4.08	–	–
**S*p*P**	12.8(±1.25)	−3.78	–	–
**S*m*P**	5.2(±0.47)	−4.17	–	–

Using either Pt‐ or graphene‐coated probes, for a given head group, the average molecular conductance, *G*_M_, of a pyridine‐based wire is higher than that of the equivalent benzene‐based wire. However, the conductance distribution of pyridine‐based wires tends to be broader than that of the benzene‐based analogues (Figures 2 a and S2.14 in the SI). This agrees with the observation above that SAMs of pyridine‐based species have lower electrical uniformity than SAMs of their benzene analogues. Given the close proximity to the Au surface, the slightly reduced steric bulk of a pyridine lone pair versus a benzene hydrogen atom could mean that more molecular conformations are possible within the Au–SAM–probe ensemble in the pyridine case. The extent of electronic coupling between the Au surface and the conductive backbone through the pyridine nitrogen, a known anchor group,^18^ could vary significantly with conformation, thus affording a broad conductance distribution.

*G*_M_ for the tetrapodal wires is 3–5 times higher in Au‐SAM‐graphene junctions than in Au–SAM–Pt junctions. Stronger molecule–probe interactions are possible in the former case as π–π overlap may occur between the head groups and graphene, which could enhance electronic coupling and therefore increase conductance. Alternatively, π–π overlap could move the electronic contact point further down the backbone, effectively shortening the conductive pathway, thereby affording a higher conductance.

For molecules with a given anchor group, *G*_M_ varies with head group according to the trend ***p*** **P** > **B>** ***m*** **P** in all cases. The ***p*** **P** head group was expected to perform best with a metallic probe, due to interactions between the probe and the pyridine lone pair, which were anticipated to enhance electronic coupling, and constructive QI effects. Interestingly, this head group also showed the highest *G*_M_ when using a graphene‐coated probe. These results are consistent with constructive (***p*** **P**) and destructive (***m*** **P**) QI effects occurring for both probes, even though in short single molecules, such effects are sensitive to the nature of the molecule–electrode contact and can be masked, due to parallel transport through the σ‐channel and π–σ mixing.^19^ The higher conductance of the **B** head group relative to ***m*** **P** suggests that π–probe interactions contribute significantly to electronic coupling for both probes, as the former has no other binding functionality.

The effect of molecular length on *G*_M_ was studied using **B*p*P** and its two analogues **B(OPE1)*p*P** and **B(OPE3)*p*P**. As seen in Figures 2 b–d, conductance decreases with molecular length, as expected for OPE‐type molecular wires. Indeed, a plot of ln(*G*_M_) against DFT‐relaxed molecular length gives the expected linear trend (Figure 2 d), allowing for estimation of the tunnelling decay factor, *β*. For this series, *β*=0.42±0.03 Å^−1^, which is slightly larger than the reported range for OPEs of 0.2–0.34 Å^−1^.2b, 18 This discrepancy could relate to the incorporation of the pyridine head group into the tetrapodal base of **B(OPE1)*p*P** in order to create a shorter analogue of **BP**. This structural change may have an additional effect on conductance and therefore distort the *β*‐value. From the plot in Figure 2 d the contact resistance of the benzene‐based anchoring unit is estimated as 9.8 MΩ.

We further investigated these three molecules and two additional, longer analogues using DFT‐based charge transport calculations with the Gollum package^20^ (see SI, Section 3.2). The logarithms of the calculated conductances of the OPE2 to OPE5 species follow the expected linear trend with molecular length, with *β*=0.21 Å^−1^ (SI, Figure S3.02). The calculated conductance of **B(OPE1)*p*P** is much higher than would be expected by extrapolating this trend, indicating that it may not be a representative member of the OPE series. In the simulations, the short conductive backbone of **B(OPE1)*p*P** results in additional electronic coupling between the top electrode and the carbazole units, which affords additional conductance pathways and a higher molecular conductance. When this additional coupling is artificially removed, the calculated conductance is lower and closer to the expected trend (SI, Figure S3.02). The linear trend observed in the cAFM experiments suggests that electronic coupling through the carbazole units is hindered in this case. This could be due to roughness in the top contact or the presence of solvent molecules.

**Figure 3:**
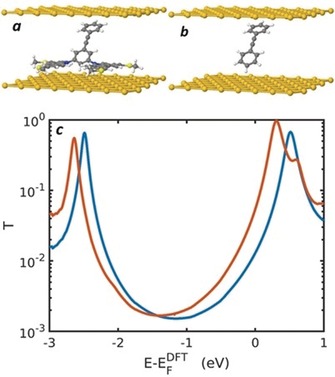
a) DFT‐relaxed geometry of a **BB** tetrapod in a molecular junction; b) a junction containing only the conductive OPE backbone of **BB**, held in the same position as the backbone in a); c) Red curve: transmission function for a **BB** tetrapod in a molecular junction as shown in a), Blue curve: transmission function for the OPE2 backbone of **BB**, with the carbazole‐based anchoring groups removed, held in a molecular junction as shown in b).

Compared to thiol‐anchored analogues **SB**, **S*p*P** and **S*m*P**, results from the Pt probe show that *G*_M_ for the benzene‐ and pyridine‐based tetrapodal wires is 5–10 times and 4–5 times lower, respectively. This is reasonable as a thiol anchor provides strong electronic coupling to a gold surface through Au‐S bond formation, whereas the conductive backbones of the tetrapodal species are unable to interact directly with the surface to the same extent. To probe the nature of the electronic coupling between the tetrapods and the gold surface, we conducted charge transport simulations in which **BB** was compared to a simple, unfunctionalised OPE2, which was held in the same geometry as the OPE2 backbone of **BB** when relaxed in a junction configuration (Figures 3 a,b). The resulting transmission functions (Figure 3 c) are remarkably similar, suggesting that the carbazole anchoring units have negligible influence on electronic coupling and therefore on G_M_, despite providing an efficient means of surface binding. In effect, our molecular design decouples surface binding from electronic coupling. This important observation potentially allows for investigations of unconventional or weakly binding anchor groups, which could be held in place near the surface by a tetrapodal unit, allowing their electronic coupling to be probed regardless of their surface binding properties.

Section 3.2 of the SI discusses additional charge‐transport calculations investigating the effects of factors such as the spacing between the molecules and the top electrode, the tilt angle of the conductive backbone in the junction, and binding geometries between the head group and top contact.

The single‐molecule conductance of several tripodal molecular wires has been reported in the literature, typically using scanning tunnelling microsocopy or mechanically controlled break‐junction methods (STM‐BJ and MCBJ, respectively). Any comparisons with our *G*_M_ values obtained using cAFM therefore require benchmarking. The molecular conductances of thiol‐anchored species **S*p*P** and **S*m*P** have been reported using the MCBJ method.^21^ Our cAFM method results in slightly lower conductances for both **S*p*P** (log *G*_M_/*G*_0_=−3.78 vs. log *G*/*G*_0_=−3.2^21^) and **S*m*P** (log *G*_M_/*G*_0_=−4.17 vs. log *G*/*G*_0_=−3.9^21^); a comparable deviation between cAFM and MCBJ has been observed previously.^22^ Such discrepancies may result from differences in the nature of the junctions or assumptions made when calculating *G*_M_ from *G*_J_. It can be concluded, however, that it is reasonable to compare our *G*_M_ values with conductances determined using MCBJ, with the caveat that the *G*_M_ values may be slight underestimates. As MCBJ conductances have been shown to be comparable with those obtained using STM‐BJ,^15^, ^23^ it follows that comparisons with STM‐BJ data are also valid.

Spirobifluorenes are amongst the most conductive tripodal molecular wires, with reported5b log *G*/*G*_0_ of −3.2 (STM‐BJ) and −3.0 (MCBJ) for a wire of similar length to our OPE2 derivatives (log *G*_M_/*G*_0_ ≤ −4.37), but with much stronger direct thiol anchoring of the conductive backbone to the gold surface. Although they are more conductive, the spirobifluorenes require a much lengthier synthesis than the tetrapods. The OPE2 tetrapods have conductances comparable to molecular wires based on tetraphenylmethane tripods, with three thiol anchors (log *G*/*G*_0_=−3.4 for a slightly shorter wire and log *G*/*G*_0_=−4.8 for a slightly longer wire, both STM‐BJ5h), pyridine anchors (log *G*/*G*_0_=−4.5 (STM‐BJ)5i) or thiophene anchors (log *G*/*G*_0_=−4.7 (STM‐BJ)5i).

## Conclusion

Tetrapodal anchor units for gold surfaces based on thiomethyl‐substituted carbazole have been developed. The synthetic route is convenient and adaptable for other possible applications. The anchor units were incorporated into OPE‐type molecular wires bearing benzene, *para*‐pyridine or *meta*‐pyridine head groups, and their conductance was investigated using cAFM and charge transport calculations. The molecules form uniform SAMs on Au(111) in which the carbazole π‐systems and sulfur atoms bind to the surface with the conductive OPE backbones protruding at an angle of 35–50°. The stabilising effect of multiple thiomethyl groups was demonstrated using DFT‐calculated binding energies, which were higher than those of conventional anchoring groups and analogues where thiomethyl groups were sequentially removed. Using cAFM, the conductance of Au–SAM–Pt and Au–SAM–graphene junctions was measured and used to determine the conductance contribution per molecule, *G*_M_, based on the contact area of the AFM probe and the area occupied by a molecule as determined by reductive desorption studies. For OPE2 tetrapods in Au–SAM–Pt junctions, *G*_M_ varied from 10^−5.20^ G_0_ (**B*m*P**) to 10^−4.37^ G_0_ (**P*p*P**). In all cases, tetrapods with pyridine in the base unit gave higher *G*_M_ than their benzene‐based analogues, but tended to show broader conductance distributions. For a given base, *G*_M_ varied with head group in the order ***p*** **P** > **B>** ***m*** **P**. For Au–SAM–graphene junctions, similar trends were observed but *G*_M_ increased by a factor of ca. 4. Charge transport calculations showed that the carbazole units play little or no role in the conductance pathway of the molecules, and that electronic coupling to the surface is through the benzene or pyridine ring of the anchoring unit. Such decoupling of surface binding and electronic coupling could enable the use of unconventional functional groups, which may afford strong electronic coupling but only weak physical binding, in tandem with ancillary strong anchor groups with poor electronic coupling. Despite the lack of strong electronic coupling between the tetrapods and the gold surface, comparison with the literature shows that their conductance is comparable to existing tripodal systems. Ongoing research in our laboratories is exploring ways to enhance the electronic coupling, and therefore molecular conductance, of systems based on a tetrapodal anchor motif, while retaining a convenient synthetic approach.

## Conflict of interest

The authors declare no conflict of interest.

## Supporting information

As a service to our authors and readers, this journal provides supporting information supplied by the authors. Such materials are peer reviewed and may be re‐organized for online delivery, but are not copy‐edited or typeset. Technical support issues arising from supporting information (other than missing files) should be addressed to the authors.

Supplementary
