# Supplementary material for: Carbazole‐Based Tetrapodal Anchor Groups for Gold Surfaces: Synthesis and Conductance Properties
Source: Angew Chem Int Ed Engl. 2019 Nov 27;59(2):882–9. doi: 10.1002/anie.201911652 (PMC7027450; doi:10.1002/anie.201911652)
Supplement: Supplementary file 1 — Supplementary [file ANIE-59-882-s001.pdf]

Angewandte Chemie
Eine Zeitschrift der Gesellschaft Deutscher Chemiker

Supporting Information

Carbazole-Based Tetrapodal Anchor Groups for Gold Surfaces: Synthesis and Conductance Properties

*Luke J. O'Driscoll, Xintai Wang, Michael Jay, Andrei S. Batsanov, Hatef Sadeghi, Colin J. Lambert, Benjamin J. Robinson, and Martin R. Bryce**

anie_201911652_sm_miscellaneous_information.pdf

Abstract: As the field of molecular-scale electronics matures and the prospect of devices incorporating molecular wires becomes more feasible, it is necessary to progress from the simple anchor groups used in fundamental conductance studies to more elaborate anchors designed with device stability in mind. This study presents a series of oligo(phenylene-ethynylene) wires with one tetrapodal anchor and a phenyl or pyridyl head group. The new anchors are designed to bind strongly to gold surfaces without disrupting the conductance pathway of the wires. Conductive probe atomic force microscopy (cAFM) was used to determine the conductance of self-assembled monolayers (SAMs) of the wires in Au−SAM−Pt and Au−SAM−graphene junctions, from which the conductance per molecule was derived. For tolane-type wires, mean conductances per molecule of up to $10^{-4.37}$ G_0 (Pt) and $10^{-3.78}$ G_0 (graphene) were measured, despite limited electronic coupling to the Au electrode, demonstrating the potential of this approach. Computational studies of the surface binding geometry and transport properties rationalise and support the experimental results.

DOI: 10.1002/anie.201911652

Table of Contents

Table of Contents 2
Experimental Procedures and Additional Discussion 2
1. Synthesis and NMR Spectra 2
1.1 General Experimental Details 2
1.2 Synthetic Procedures. 3
1.3 ^{1}H NMR Spectra 16
1.4 ^{13}C NMR Spectra 30
1.5 Recently Developed Anchoring Groups for Gold Surfaces 44
1.6 Synthesis of Model Compound **1** 44
1.7 Optimisation of the Synthesis of *N*-*tert*-Butyldimethylsilyl-3,6-bis(methylthio)carbazole, **6** 44
1.8 Synthesis of Thioacetate Functionalised Series 46
2. Materials Characterisation and Properties 48
2.1 X-Ray Crystallography 48
2.2 UV-Visible Absorption Spectroscopy 49
2.3 SAM Formation and Imaging 51
2.4 Reductive Desorption 52
2.5 Quartz Crystal Microbalance 53
2.6 Conductive AFM 54
2.7 SAM Stability 58
3. Computational Studies 60
3.1 Geometry Optimisation 60
3.2 Charge Transport Studies 61
References 66
Author Contributions 66

Experimental Procedures and Additional Discussion

1. Synthesis and NMR Spectra

1.1 General Experimental Details

Reagents were purchased commercially and used as received unless otherwise stated. Dimethyl disulfide was stored over molecular sieves. The concentration of ^{t}BuLi was determined by titration according to a literature method[1] prior to use. Anhydrous solvents were prepared using an Innovative Technology solvent purification system and stored in ampoules under argon, with the exception of anhydrous DMSO and DIPEA (both Acros Organics Acroseal®, used as received). Thin-layer chromatography (TLC) analysis was carried out using Merck silica gel 60 F_{254} TLC plates and spots were visualised using a UV lamp emitting at 365 or 254 nm. Column chromatography was performed using silica gel 60A (40-63 µm) purchased from Fluorochem. ^{1}H and ^{13}C NMR spectroscopy was carried out on a Bruker AV400 NMR spectrometer. For ^{1}H NMR spectra, chemical shifts are reported relative to the residual solvent peak (7.26 ppm for $CHCl_3$) and for ^{13}C NMR spectra, chemical shifts are reported relative to the solvent peak (77.16 ppm for $CDCl_3$). These, and any other residual solvent peaks were referenced to values reported in the literature.[2] All NMR spectra were processed using MestReNova V12. ASAP mass spectrometry was carried out using an LCT Premier XE mass spectrometer (Waters Ltd, UK) using TOF detection. Exact mass measurements were processed using Elemental Composition 4.0 embedded within MassLynx 4.1 (Waters Ltd, UK). Melting points were determined using a Stuart SMP40 machine with a ramping rate of 3 °C min^{-1}. The videos produced by the machine were analysed manually to determine the melting point. Elemental analysis was performed on an Exeter Analytical E-440 machine.

Unless otherwise stated, reactions were conducted under an argon atmosphere. Glassware was first dried under vacuum using a heat gun, then filled directly with argon. Solvents and liquid reagents were added by syringe or cannula, and solid reagents were added under a positive pressure of argon. Degassing was conducted by bubbling argon through the reaction mixture using an argon-filled balloon fitted with a syringe needle. All mixed solvents in this work were prepared as v/v mixtures.

1.2 Synthetic Procedures.

***N-tert*-Butyldimethylsilyl-3,6-diiodocarbazole**, **7**

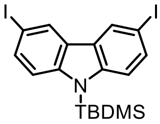

This synthesis was prepared based on a reported protection of 3-bromocarbazole.[3]

3,6-Diiodocarbazole[4] (4.00 g, 9.55 mmol) was dissolved in anhydrous THF (50 mL) at RT in a dry flask under argon. Sodium hydride (344 mg, 14.3 mmol, 1.5 eq.) was added to the stirred solution (*N.B. effervescence*) which was stirred at RT for a further 30 min. *tert*-Butyldimethylsilyl chloride (1.58 g, 10.5 mmol, 1.1 eq.) was then added and the reaction stirred at RT for a further 17 h. After this time, the reaction was poured carefully into ice water (100 mL) and extracted with DCM (3 × 75 mL). The combined organic layers were dried ($MgSO_4$) before the solvent was removed *in vacuo*, affording an off-white solid (ca. 4.9 g). The crude product was passed through a plug of silica (150 mL) in a fritted funnel, using 9:1 hexane/DCM (500 mL) as eluent. After removing the solvent *in vacuo*, **7** was obtained as a white solid (4.44 g, 87%).

m.p.: 183–184.5 °C

^{1}H NMR (400 MHz, $CDCl_3$) δ 8.30 (dd, *J* = 1.9, 0.5 Hz, 2H), 7.62 (dd, *J* = 8.8, 1.9 Hz, 2H), 7.36 (dd, *J* = 8.8, 0.5 Hz, 2H), 1.00 (s, 9H), 0.73 (s, 6H).

^{13}C NMR (101 MHz, $CDCl_3$) δ 144.4, 134.4, 129.0, 127.7, 116.2, 82.9, 26.6, 20.7, -1.2.

MS: ASAP: 532.9 $[M]^{+\cdot}$, 407.0 $[M-I+H]^{+\cdot}$

HRMS: m/z: $[M]^{+\cdot}$ calculated for $C_{18}H_{21}NSiI_2$, 532.9533; found, 532.9529

Characterisation is in agreement with that reported in the literature for a similar synthetic route.[5]

***N-tert*-butyldimethylsilyl-3,6-bis(methylthio)carbazole**, **6**

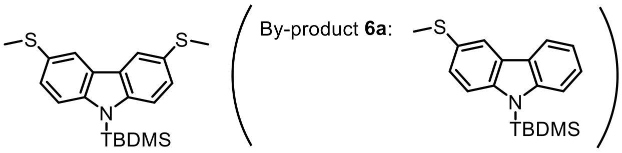

This synthesis was based on a reported general procedure.[6] Optimisation of the conditions was required due to extensive by-product formation when the reported method was used (see Section 1.7 below).

N.B. Unpleasant-smelling by-products, presumed to include methanethiol, form in this reaction. We recommend treating the aqueous layers and used glassware with bleach overnight to destroy these materials, and to ensure appropriate containment is used when handling the organic layers.

Diiodide **7** (1.90 g, 3.56 mmol) was dissolved in anhydrous THF (25 mL) in a dry flask under argon. The solution was cooled to −78 °C (dry ice/acetone bath) and degassed (argon, 10 min). *tert*-Butyllithium (0.99 M in heptane,[1] 9.00 mL, 8.91 mmol, 2.5 eq.) was then carefully added to the cooled reaction mixture over 10 min (solution changes from colourless to yellow). After this addition, the reaction was stirred at −78 °C for 10 min before dimethyl disulfide (1.28 mL, 14.3 mmol, 4.0 eq.) was added slowly (solution changes from yellow to colourless) and the reaction was allowed to warm slowly to RT over ca. 90 min. After this time TLC (5:1 hexane/DCM) indicated the reaction was complete, and the reaction was quenched by pouring into deionised water (75 mL). The mixture was extracted with DCM (75 mL, then 2 × 50 mL) and the combined organic layers were washed with brine (2 × 75 mL), then dried ($MgSO_4$) before the solvent was removed *in vacuo*, affording a thick, off-white oil (1.42 g). The crude product was purified by column chromatography (5 cm Ø, 250 mL SiO_2); the column was prepared and the crude material loaded using hexane, then 5:1 hexane/DCM was used to elute the by-product **6a** (204 mg white solid, 17%) before switching to 2:1 hexane/DCM to elute **6**, which, after removal of the solvent *in vacuo*, was obtained as a colourless oil which slowly solidified into a white solid (1.10 g, 83%).

m.p.: 81.5–83.5 °C

^{1}H NMR (400 MHz, $CDCl_3$) δ 8.00 (dd, *J* = 2.0, 0.6 Hz, 2H), 7.51 (dd, *J* = 8.7, 0.6 Hz, 2H), 7.37 (dd, *J* = 8.7, 2.0 Hz, 2H), 2.57 (s, 6H), 1.02 (s, 9H), 0.73 (s, 6H).

^{13}C NMR (101 MHz, $CDCl_3$) δ 144.1, 128.3, 127.2, 126.6, 120.4, 114.7, 26.7, 20.7, 18.6, -1.2.

MS: ASAP: 374.1 $[M+H]^+$, 373.1 $[M]^{+\cdot}$

HRMS: m/z: $[M]^{+\cdot}$ calculated for $C_{20}H_{27}NS_2Si$, 373.1354; found, 373.1357

elem.: Anal. Calcd for $C_{20}H_{27}NS_2Si$: C, 64.29; H, 7.28; N, 3.75. Found: C, 64.23; H, 7.21; N, 3.61.

3,6-bis(methylthio)carbazole, **2**

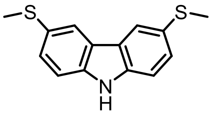

Dry conditions were not used for this reaction. *N*-Protected carbazole **6** (1.55 g, 4.14 mmol) was dissolved in THF (15 mL) and stirred at RT. TBAF (1.0 M in THF, 6.2 mL, 6.2 mmol, 1.5 eq.) was added and the reaction stirred at RT for 10 min, after which TLC (1:1 hexane/DCM) indicated complete conversion. 15 min after the addition of TBAF, the reaction was quenched with saturated $NH_4Cl_{(aq)}$ (50 mL) and extracted with toluene (3 × 50 mL). The combined organic layers were washed with deionised water (3 × 50 mL)* and brine (50 mL), then dried ($MgSO_4$) before the solvent was removed *in vacuo* to afford **2** as a white solid (1.02 g, 95%) with no further purification.

m.p.: 83–85.5 °C (lit.[7] 89–90 °C)

^{1}H NMR (400 MHz, $CDCl_3$) δ 8.06 – 8.03 (m, 2H), 8.02 (bs, 1H), 7.44 (dd, *J* = 8.4, 1.8 Hz, 2H), 7.35 (dd, *J* = 8.4, 0.7 Hz, 2H), 2.57 (s, 6H).

^{13}C NMR (101 MHz, $CDCl_3$) δ 138.6, 128.2, 123.7, 121.3, 111.4, 19.0†

MS: ASAP: 260.1 $[M+H]^+$, 259.0 $[M]^{+\cdot}$

HRMS: $[M+H]^+$ calculated for $C_{14}H_{14}NS_2$, 260.0568; found, 260.0555

elem.: Anal. Calcd for $C_{14}H_{13}NS_2$: C, 64.83; H, 5.05; N, 5.40. Found: C, 64.81; H, 5.13; N, 5.28.

We can find only one previous report of this compound,[7] in which m.p. and elemental analysis are the only reported characterisation techniques.

Benzene Base Precursor, **3**

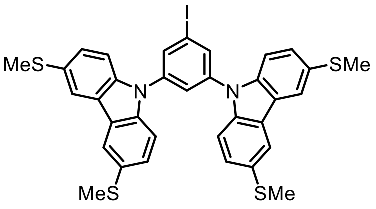

2 (477 mg, 1.84 mmol, 2.2 eq.) was dissolved in anhydrous DMF (30 mL) in a dry flask under argon at RT. Caesium carbonate (952 mg, 2.92 mmol, 3.5 eq.) was added (solution turns yellow) and the mixture was stirred for 15 min at RT. 3,5-Difluoroiodobenzene (0.1 mL, 0.835 mmol, 1.0 eq.) was then added and the mixture set to heat at 100 °C. After 17 h, TLC (2:1 DCM/hexane) indicated that the reaction was complete. The mixture was allowed to cool to RT then diluted with DCM (20 mL) and poured into brine (50 mL), washing the reaction flask with deionised water (10 mL) to dissolve residual solids. The mixture was extracted with DCM (3 × 50 mL) and the combined organic layers were washed with brine (50 mL) then dried ($MgSO_4$) before removing the solvent *in vacuo*‡, affording a yellow solid (610 mg). The crude product was dissolved in DCM and evaporated onto celite (15 mL) then purified by column chromatography (5 cm Ø, 300 mL SiO_2, eluent: 2:1 DCM/hexane with 1% NEt_3). After removal of the solvent *in vacuo*, **3** was obtained as a white solid (456 mg, 76%).

m.p.: 215–217 °C

^{1}H NMR (400 MHz, $CDCl_3$) δ 8.07 (dd, *J* = 1.8, 0.6 Hz, 4H), 8.00 (d, *J* = 1.9 Hz, 2H), 7.70 (t, *J* = 1.9 Hz, 1H), 7.46 (dd, *J* = 8.6, 1.7 Hz, 4H), 7.42 (dd, *J* = 8.6, 0.6 Hz, 7H), 2.59 (s, 12H).

---

* N.B. Slow layer separation.
† Five aromatic signals are seen rather than the expected six – two signals are presumed to overlap.
‡ Heptane (ca. 50 mL) was used to facilitate the removal of residual DMF as an azeotrope.

^{13}C NMR (101 MHz, $CDCl_3$) δ 140.1, 139.2, 134.5, 130.0, 128.1, 124.3, 124.0, 121.0, 110.3, 95.4, 18.6.

MS: (MALDI, 71) ASAP: 719.0 $[M+H]^+$, 718.0 $[M]^{+\cdot}$

HRMS: $[M]^{+\cdot}$ calculated for $C_{34}H_{27}IN_2S_4$, 718.0102; found, 718.0091

elem.: Anal. Calcd for $C_{34}H_{27}IN_2S_4$: C, 56.82; H, 3.79; N, 3.90. Found: C, 57.07; H, 3.75; N, 3.88.

Pyridine Base Precursor, **4**

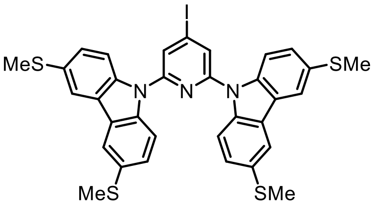

Attempts to use conditions comparable to those used in the preparation of **3** resulted in extensive side reactions. Milder, alternative conditions based on a literature study of S_NAr reactions of polyhalogenated pyridines were used instead.[8]

2 (497 mg, 1.92 mmol, 2.2 eq.) and potassium carbonate (265 mg, 1.92 mmol, 2.2 eq.) were dissolved/suspended in anhydrous DMSO (5 mL) in a dry flask under argon and stirred at RT for 15 min. 2,6-Difluoro-4-iodopyridine (210 mg, 0.871 mmol, 1.0 eq.) was then added and the mixture set to heat at 70 °C; by the time this temperature was reached all suspended material had dissolved. After 1 h the reaction had thickened to a paste, and further anhydrous DMSO (5 mL) was added to aid stirring. After a further 1 h TLC (2:1 DCM/hexane) indicated the reaction was complete and the reaction was cooled to RT, diluted with DCM (40 mL) and poured into brine (100 mL), washing the reaction flask with deionised water (10 mL) to dissolve residual solids. The layers were separated and the aqueous layer was washed with further DCM (2 × 50 mL). The organic layers were combined and washed with brine (50 mL) then dried ($MgSO_4$) before the solvent was removed *in vacuo* to afford a cream solid (665 mg). The crude product was purified by repeated column chromatography. The first two columns (5 cm Ø, 300 mL SiO_2, eluent: 2:1 DCM/hexane, then 3 cm Ø, 250 mL SiO_2, eluent: 5:1 DCM/hexane) each afforded some pure product but predominantly product contaminated with residual **2**. After evaporating the contaminated product onto celite (10 mL) from DCM, a third column (3 cm Ø, 200 mL SiO_2, eluent: 5:1 DCM/hexane with 1% NEt_3) separated the two materials. The pure product from each column was combined to afford **4** as a yellow solid (523 mg, 83%).

m.p.: 241.5–244 °C

^{1}H NMR (400 MHz, $CDCl_3$) δ 8.02 (dd, J = 1.9, 0.6 Hz, 4H), 7.94 (dd, J = 8.7, 0.6 Hz, 4H), 7.90 (s, 2H), 7.40 (dd, J = 8.7, 1.9 Hz, 4H), 2.59 (s, 12H).

^{13}C NMR (101 MHz, $CDCl_3$) δ 151.3, 137.9, 131.2, 127.7, 125.2, 122.7, 120.0, 112.9, 107.8, 18.1.

MS: ASAP: 720.0 $[M+H]^+$, 719.0 $[M]^{+\cdot}$

HRMS: $[M]^{+\cdot}$ calculated for $C_{33}H_{26}IN_3S_4$, 719.0054; found, 719.0051

elem.: Anal. Calcd for $C_{33}H_{26}IN_3S_4$: C, 55.07; H, 3.64; N, 5.84. Found: C, 55.01; H, 3.64; N, 5.72.

General Procedure for Sonogashira couplings

The appropriate aryl iodide (1 eq., typically ca. 0.2 mmol – see below) and alkyne (1.1 eq., typically ca. 0.22 mmol – see below) were dissolved in a mixture of anhydrous THF (8 mL) and anhydrous DIPEA (2 mL) in a dry flask under argon and the mixture was degassed (argon, 30 min). CuI (5 mol %) and $Pd(PPh_3)_2Cl_2$ (5 mol %) were added and the mixture was degassed for a further 5 min then left to stir at RT until TLC indicated the reaction was complete (typically 80–120 min – see below). The reaction was then diluted with DCM (20 mL) and extracted with either brine (3 × 25 mL), for pyridine-containing species, or otherwise sat. $NH_4Cl_{(aq)}$ (3 × 25 mL). The organic phase was dried ($MgSO_4$) and the solvent removed *in vacuo** to afford crude product which was purified by column chromatography (see below), and further purified by recrystallisation if necessary.

* Where large quantities of DIPEA remained in the organic layer, toluene was added to facilitate removal as an azeotrope.

BB

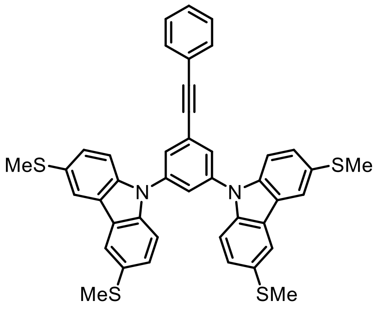

Prepared according to the general procedure for Sonogashira couplings using the following quantities of reagents: iodide **3** (150 mg, 0.209 mmol), phenylacetylene (25 µL, 0.23 mmol), CuI (2 mg, 0.01 mmol) and $Pd(PPh_3)_2Cl_2$ (7 mg, 0.01 mmol). Reaction time: 80 min. The crude product was a brown solid (189 mg) which was purified by column chromatography (3 cm Ø, 100 mL SiO_2) eluting with 2:1 hexane/DCM then 1:1 hexane/DCM to remove, respectively, two yellow bands (both impurities) then 1:2 hexane/DCM to elute **BB**. Removal of the solvent *in vacuo* afforded a yellow solid (130 mg), which was not fully pure.* This material was recrystallised twice, firstly by dissolving in a minimum of DCM (ca. 4 mL) upon which hexane (ca. 8 mL) was layered in a closed vial; a fluffy cream solid precipitated as the two layers mixed overnight. The precipitate was isolated by filtration, washing with pentane†, then recrystallised a second time in a similar fashion, this time layering pentane (ca. 6 mL) onto a DCM (ca. 4 mL) solution of product. Analytically pure **BB** was obtained as a fluffy cream solid (98 mg, 68%).

m.p.: 234.5–236 °C

^{1}H NMR (400 MHz, $CDCl_3$) δ 8.09 (apparent t, *J* = 1.2 Hz, 4H), 7.81 (d, *J* = 2.0 Hz, 2H), 7.68 (t, *J* = 2.0 Hz, 1H), 7.59 – 7.54 (m, 2H), 7.47 (apparent d, *J* = 1.2 Hz, 8H), 7.41 – 7.36 (m, 3H), 2.59 (s, 12H).

^{13}C NMR (101 MHz, $CDCl_3$) δ 139.4, 131.9, 129.7, 129.2, 128.7, 128.6, 128.2, 127.3, 124.7, 124.0, 122.4, 121.1, 110.5, 92.5, 87.5, 18.7.‡

MS: ASAP: 693.1 $[M+H]^+$, 692.1 $[M]^{+\cdot}$

HRMS: $[M+H]^+$ calculated for $C_{42}H_{33}N_2S_4$, 693.1527; found, 693.1547

elem.: Anal. Calcd for $C_{42}H_{32}N_2S_4$: C, 72.80; H, 4.65; N, 4.04. Found: C, 72.81; H, 4.54; N, 4.03.

PB

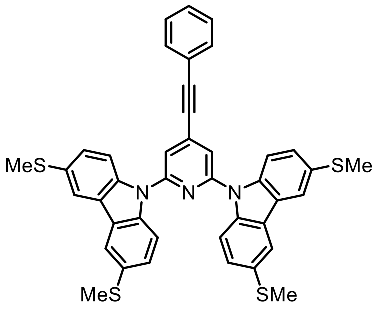

Prepared according to the general procedure for Sonogashira couplings using the following quantities of reagents: iodide **4** (149 mg, 0.207 mmol), phenylacetylene (25 µL, 0.23 mmol), CuI (2 mg, 0.01 mmol) and $Pd(PPh_3)_2Cl_2$ (7 mg, 0.01 mmol). Reaction time: 90 min. The crude product was a yellow solid (204 mg) which was purified by column chromatography (2 cm Ø, 50 mL SiO_2, eluent: 1:1 hexane/DCM with 1% NEt_3). Removal of the solvent *in vacuo* afforded slightly impure§ **PB** as a yellow solid (134 mg). This material was recrystallised by dissolving in a minimum of DCM (ca. 2 mL) upon which pentane (ca. 3 mL) was layered in a closed vial. No precipitate formed after the layers mixed overnight. Layering additional

* ^{1}H NMR spectroscopy showed small additional signals in the aromatic region.
† Again, a satisfactory ^{1}H NMR spectrum was not obtained, although the impurity signals were now smaller.
‡ Thirteen aromatic signals are seen rather than the expected fourteen – two signals are presumed to overlap.
§ ^{1}H NMR spectroscopy showed small additional signals in the aromatic region.

pentane (ca. 3 mL) onto the solution and standing overnight afforded a precipitate, which was isolated by filtration and washed with pentane to give analytically pure **PB** as a yellow solid (94 mg, 65%).

m.p.: 231–233.5 °C

^{1}H NMR (400 MHz, $CDCl_3$) δ 8.05 (d*, *J* = 1.9 Hz, 4H), 7.98 (d, *J* = 8.7 Hz, 4H), 7.64 (s, 2H), 7.63 – 7.60 (m, 2H), 7.46 – 7.39 (m†, 7H), 2.60 (s, 12H).

^{13}C NMR (101 MHz, $CDCl_3$) δ 151.6, 138.1, 136.2, 132.2, 130.8, 129.9, 128.8, 127.8, 125.0, 121.7, 120.2, 116.1, 113.0, 95.7, 86.4, 18.3.

MS: ASAP: 694.1 $[M+H]^+$, 693.1 $[M]^{+\cdot}$

HRMS: $[M]^{+\cdot}$ calculated for $C_{41}H_{31}N_3S_4$, 693.1401; found, 693.1425

elem.: Anal. Calcd for $C_{41}H_{31}N_3S_4$: C, 70.96; H, 4.50; N, 6.06. Found: C, 70.67; H, 4.42; N, 6.00.

B*p*P

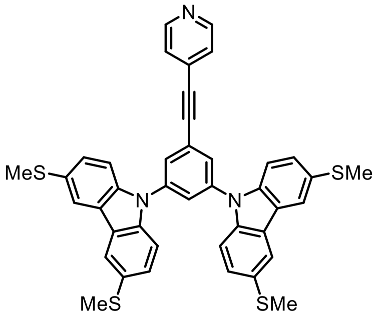

Prepared according to the general procedure for Sonogashira couplings using the following quantities of reagents: iodide **3** (145 mg, 0.202 mmol), 4-ethynylpyridine (23 mg, 0.22 mmol), CuI (2 mg, 0.01 mmol) and $Pd(PPh_3)_2Cl_2$ (7 mg, 0.01 mmol). Reaction time: 80 min. The crude product was a yellow solid (164 mg) which was purified by column chromatography (2 cm Ø, 50 mL SiO_2) eluting with DCM to remove a yellow band (impurity) then 10:1 DCM/acetone to elute **B*p*P** (yellow band). Removal of the solvent *in vacuo* afforded analytically pure **B*p*P** as a yellow solid (112 mg, 80%).

m.p.: 216–218 °C

^{1}H NMR (400 MHz, $CDCl_3$) δ 8.65 (apparent dd‡, *J* = 4.5, 1.6 Hz, 2H), 8.09 (apparent t, *J* = 1.3 Hz, 4H), 7.83 (d, *J* = 2.0 Hz, 2H), 7.75 (t, *J* = 2.0 Hz, 1H), 7.49 – 7.44 (m, 8H), 7.41 (apparent dd, *J* = 4.5, 1.6 Hz, 2H), 2.59 (s, 12H).

^{13}C NMR (101 MHz, $CDCl_3$) δ 150.1, 139.7, 139.3, 130.5, 129.9, 128.7, 128.1, 126.0, 125.7, 125.6, 124.1, 121.0, 110.4, 91.6, 89.4, 18.7.

MS: ASAP: 694.1 $[M+H]^+$, 693.1 $[M]^{+\cdot}$

HRMS: $[M+H]^+$ calculated for $C_{41}H_{32}N_3S_4$, 694.1479; found, 694.1470

elem.: Anal. Calcd for $C_{41}H_{31}N_3S_4$: C, 70.96; H, 4.50; N, 6.06. Found: C, 70.97; H, 4.42; N, 6.07.

* This signal and that at 7.98 ppm may also couple to one another with a J value < 0.8 ppm, but this is not fully resolved at this frequency.
† This comprises two overlapping signals, a multiplet across the whole shift range (3H) and a signal which appears to be δ ≈ 7.42 (dd, J ≈ 8.7, 1.9 Hz, 4H).
‡ This signal, and that at 7.41 ppm, are each AA'XX' type but resemble dd signals with the stated *J*-couplings.

P*p*P

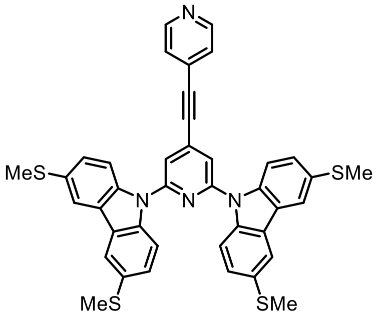

Prepared according to the general procedure for Sonogashira couplings using the following quantities of reagents: iodide **4** (152 mg, 0.211 mmol), 4-ethynylpyridine (24 mg, 0.23 mmol), CuI (2 mg, 0.01 mmol) and $Pd(PPh_3)_2Cl_2$ (7.5 mg, 0.01 mmol). Reaction time: 90 min. The crude product was a yellow solid (204 mg) which was purified by column chromatography (2 cm Ø, 50 mL SiO_2) eluting with DCM to remove two yellow bands (impurities) then 10:1 DCM/acetone to elute **P*p*P** (yellow band). Removal of the solvent *in vacuo* afforded **P*p*P** as a yellow solid (101 mg). Recrystallisation was required to achieve satisfactory elemental analysis results. The compound was dissolved in DCM (ca. 2 mL) upon which pentane (ca. 3 mL) was layered in a closed vial; a yellow precipitate formed as the two layers mixed overnight. After isolation by filtration and washing with pentane, analytically pure **P*p*P** was obtained as a yellow solid (83 mg, 57%).

m.p.: 228.5–230 °C

^{1}H NMR (400 MHz, $CDCl_3$) δ 8.69 (apparent dd*, *J* = 4.6, 1.5 Hz, 2H), 8.04 (d†, *J* = 1.9 Hz, 4H), 7.98 (d, *J* = 8.7 Hz, 4H), 7.65 (s, 2H), 7.47 (dd, *J* = 4.6, 1.5 Hz, 2H), 7.41 (dd, *J* = 8.7, 1.9 Hz, 4H), 2.60 (s, 12H).

^{13}C NMR (101 MHz, $CDCl_3$) δ 151.7, 150.2, 138.0, 134.8, 131.1, 129.8, 127.7, 125.8, 125.1, 120.1, 115.8, 112.9, 92.1, 90.1, 18.2.

MS: ASAP: 695.1 $[M+H]^+$, 694.1 $[M]^{+\cdot}$

HRMS: $[M+H]^+$ calculated for $C_{40}H_{31}N_4S_4$, 695.1432; found, 695.1437

elem.: Anal. Calcd for $C_{40}H_{30}N_4S_4$: C, 69.13; H, 4.35; N, 8.06. Found: C, 68.75; H, 4.35; N, 7.97.

B*m*P

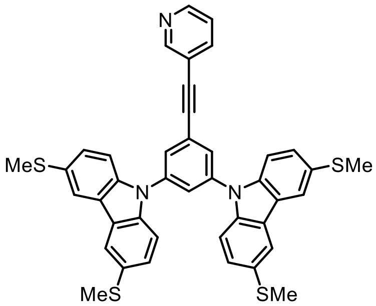

Prepared according to the general procedure for Sonogashira couplings using the following quantities of reagents: iodide **3** (152 mg, 0.211 mmol), 3-ethynylpyridine (24 mg, 0.23 mmol), CuI (2 mg, 0.01 mmol) and $Pd(PPh_3)_2Cl_2$ (7.5 mg, 0.01 mmol). Reaction time: 120 min (additional 3-ethynylpyridine (4 mg, 0.04 mmol, 0.2 eq.) was added after 90 min as the reaction was incomplete but this material had been consumed). The crude product was a yellow solid (208 mg) which was purified by column chromatography (2 cm Ø, 50 mL SiO_2) eluting with DCM to remove a yellow band (impurity) then 10:1 DCM/acetone to elute **B*m*P** (pale yellow band). Removal of the solvent *in vacuo* afforded analytically pure **B*m*P** as a cream solid (131 mg, 89%). Crystals for X-ray crystallography were grown by dissolving a small portion of material in a minimum of chloroform and layering with approximately double the volume of pentane in a sealed vial; single crystals grew after slow diffusion of the layers.

* This signal, and that at 7.47 ppm, are each AA'XX' type but resemble dd signals with the stated *J*-couplings.
† This signal and that at 7.98 ppm may also couple to one another with a J value < 0.8 ppm, but this is not fully resolved at this frequency.

m.p.: 258–260 °C

^{1}H NMR (400 MHz, $CDCl_3$) δ 8.81 (dd, *J* = 2.1, 0.8 Hz, 1H), 8.60 (dd, *J* = 4.9, 1.6 Hz, 1H), 8.09 (apparent t, *J* = 1.2 Hz, 4H), 7.85 (ddd[*], *J* = 7.9, 2.1, 1.6 Hz, 1H), 7.82 (d, *J* = 2.0 Hz, 2H), 7.73 (t, *J* = 2.0 Hz, 1H), 7.47 (apparent d, *J* = 1.2 Hz, 8H), 7.32 (ddd, *J* = 7.9, 4.9, 0.8 Hz, 1H), 2.59 (s, 12H).

^{13}C NMR (101 MHz, $CDCl_3$) δ 152.5, 149.5, 139.6, 139.4, 138.8, 129.8, 128.6, 128.2, 126.5, 125.3, 124.0, 123.3, 121.0, 119.7, 110.4, 90.6, 88.9, 18.7.

MS: ASAP: 694.1 $[M+H]^+$, 693.1 $[M]^{+\cdot}$

HRMS: $[M]^{+\cdot}$ calculated for $C_{41}H_{31}N_3S_4$, 693.1401; found, 693.1384

elem.: Anal. Calcd for $C_{41}H_{31}N_3S_4$: C, 70.96; H, 4.50; N, 6.06. Found: C, 70.97; H, 4.47; N, 5.98.

P*m*P

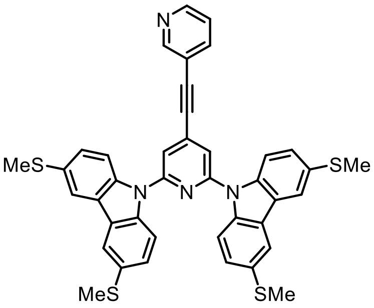

Prepared according to the general procedure for Sonogashira couplings using the following quantities of reagents: iodide **4** (152 mg, 0.211 mmol), 3-ethynylpyridine (24 mg, 0.23 mmol), CuI (2 mg, 0.01 mmol) and $Pd(PPh_3)_2Cl_2$ (7.5 mg, 0.01 mmol). Reaction time: 120 min (additional 3-ethynylpyridine (4 mg, 0.04 mmol, 0.2 eq.) was added after 90 min as the reaction was incomplete but this material had been consumed). The crude product was a yellow solid (186 mg) which was purified by column chromatography (2 cm Ø, 50 mL SiO_2) eluting with DCM to remove a yellow band (impurity) then 10:1 DCM/acetone to elute **P*m*P** (yellow band). Removal of the solvent *in vacuo* afforded analytically pure **P*m*P** as a yellow solid (133 mg, 91%).

m.p.: decomp. ca. 145–150 °C

^{1}H NMR (400 MHz, $CDCl_3$) δ 8.87 (dd, *J* = 2.2, 0.9 Hz, 1H), 8.66 (dd, *J* = 4.9, 1.7 Hz, 1H), 8.05 (d[†], *J* = 1.9 Hz, 4H), 7.99 (d, *J* = 8.7 Hz, 4H), 7.91 (ddd, *J* = 7.9, 2.2, 1.7 Hz, 1H), 7.65 (s, 2H), 7.42 (dd, *J* = 8.7, 1.9 Hz, 4H), 7.37 (ddd, *J* = 7.9, 4.9, 0.9 Hz, 1H), 2.60 (s, 12H).

^{13}C NMR (101 MHz, $CDCl_3$) δ 152.8, 151.7, 150.0, 139.1, 138.1, 135.3, 131.0, 127.8, 125.1, 123.4, 120.1, 119.0, 115.9, 113.0, 91.9, 89.4, 18.2.

MS: ASAP: 695.1 $[M+H]^+$, 694.1 $[M]^{+\cdot}$

HRMS: $[M+H]^+$ calculated for $C_{40}H_{31}N_4S_4$, 695.1432; found, 695.1416

elem.: Anal. Calcd for $C_{40}H_{30}N_4S_4$: C, 69.13; H, 4.35; N, 8.06. Found: C, 68.80; H, 4.39; N, 7.90.

[*] Overlap of peaks causes this signal to resemble a dt, but the reported ddd correctly accounts for the other observed couplings.
[†] This signal and that at 7.99 ppm may also couple to one another with a J value < 0.8 ppm, but this is not fully resolved at this frequency.

B(OPE1)*p*P

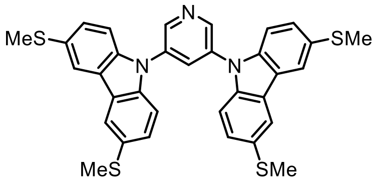

2 (187 mg, 0.721 mmol, 2.2 eq.) was dissolved in anhydrous DMF (15 mL) in a dry flask under argon at RT. Caesium carbonate (235 mg, 0.721 mmol, 2.2 eq.) was added (solution turns yellow) and the mixture was stirred for 15 min at RT. 3,5-difluoropyridine (0.03 mL, 0.33 mmol, 1.0 eq.) was then added and the mixture set to heat at 100 °C. After 17 h, TLC (2:1 DCM/hexane) indicated that the reaction was complete. The mixture was allowed to cool to RT then diluted with DCM (10 mL) and poured into brine (75 mL), washing the reaction flask with deionised water (10 mL) to dissolve residual solids. The mixture was extracted with DCM (2 × 25 mL) and the combined organic layers were washed with brine (50 mL) then dried ($MgSO_4$) before removing the solvent *in vacuo**, affording a light brown solid (221 mg). The crude product was dissolved in DCM and evaporated onto celite (10 mL) then purified by column chromatography (2 cm Ø, 80 mL SiO_2, eluent: 2:1 DCM/hexane with 1% NEt_3). After removal of solvent *in vacuo*, a mixture of **B(OPE1)*p*P** and a monosubstituted by-product was obtained. The majority of the more volatile by-product was removed by sublimation in a kugelrohr (250 °C, ca. 35 mbar), then the residue was recrystallised by dissolving in DCM and layering with pentane, affording a beige solid (76 mg). Similarly to **B(OPE3)*p*P** (below), some pyridyl environments appeared broad in the ^{1}H NMR spectrum. This was resolved by dissolving this material in DCM (20 mL) and washing with 2M $NaOH_{(aq)}$ (3 × 20 mL), drying ($MgSO_4$), and removing the solvent *in vacuo*, after which **B(OPE1)*p*P** was obtained as a cream solid (68 mg, 35%).

m.p.: decomp. ca. 255 °C

^{1}H NMR (400 MHz, $CDCl_3$) δ 8.97 (d, J = 2.2 Hz, 2H), 8.10 – 8.07 (m†, 5H), 7.46 (dd, J = 8.6, 1.8 Hz, 4H), 7.42 (dd, J = 8.6, 0.7 Hz, 4H), 2.60 (s, 12H).

^{13}C NMR (101 MHz, $CDCl_3$) δ 146.5, 139.2, 135.5, 131.5, 130.4, 128.1, 124.3, 120.9, 110.0, 18.5.

MS: ASAP: 594.1 $[M+H]^+$, 593.1 $[M]^{+\cdot}$

HRMS: $[M+H]^+$ calculated for $C_{33}H_{28}N_3S_4$, 594.1166; found, 594.1171

elem.: Anal. Calcd for $C_{33}H_{27}N_3S_4$: C, 66.75; H, 4.58; N, 7.08. Found: C, 66.23‡; H, 4.35; N, 6.97.

4-((4-ethynylphenyl)ethynyl)pyridine, **9**

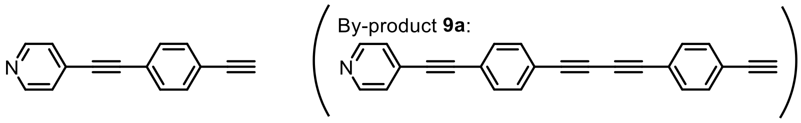

Literature reports of this compound typically cite a longer route involving a TMS-protected alkyne.[9] We have found one report that uses analogous conditions (although at 1:1 stoichiometry) and achieves a comparable yield.[10]

Prepared based on the general procedure for Sonogashira couplings on a larger scale and with adjusted stoichiometry to favour monosubstitution of the diyne. The following quantities of reagents were used: 4-iodopyridine (750 mg, 3.66 mmol), 1,4-diethynylbenzene (1.15 g, 9.12 mmol, 2.5 eq.), CuI (35 mg, 0.18 mmol), $Pd(PPh_3)_2Cl_2$ (128 mg, 0.18 mmol), THF (16 mL) and DIPEA (4 mL). Reaction time: 120 min. The crude product was a yellow solid (1.68 g), which was purified by column chromatography (5 cm Ø, 250 mL SiO_2) eluting with DCM to remove less-polar impurities (unreacted 1,4-diethynylbenzene and homocoupled derivatives) then 10:1 DCM/acetone to elute **9** contaminated with **9a**§ (459 mg). Attempts to remove the contaminant by recrystallisation and further chromatography (eluting with a gradient from toluene to 8:1 toluene/EtOAc) were unsuccessful. Sublimation of the mixture in a kugelrohr (ca. 180 °C, 0.2 mbar) afforded pure **9** as a white solid (299 mg, 40%).

* Heptane (ca. 50 mL) was used to facilitate the removal of residual DMF as an azeotrope.
† This signal is not clearly resolved, but should be 2 overlapping signals: a dd (based on other couplings, expect J = 1.8, 0.7 Hz, 4H) and a t (based on other couplings, expect J = 2.2 Hz, 1H).
‡ The %C value differs from the calculated value by ~0.5%, rather than the usual tolerance of ±0.4%, but due to the small quantity of material isolated measurements were conducted without further purification. The NMR spectra of this material are comparable to those obtained for its analogues which did provide satisfactory elemental analysis data.
§ Identified by its presence in an ASAP mass spectrum (m/z = 328.1 $[M+H]^+$). ^{1}H NMR integrals suggest ca. 10 mol % of this by-product was present.

m.p.: 179–181 °C (lit 178−180 °C[10])

^{1}H NMR (400 MHz, $CDCl_3$) δ 8.63 – 8.60 (m, 2H), 7.50 (s, 4H), 7.39 – 7.36 (m, 2H), 3.20 (s, 1H).

^{13}C NMR (101 MHz, $CDCl_3$) δ 150.0, 132.3, 131.9, 131.2, 125.6, 123.1, 122.6, 93.3, 88.6, 83.1, 79.6.

MS: ASAP: 407.2 $[2M+H]^+$, 204.1 $[M+H]^+$, 203.1 $[M]^{+\cdot}$

HRMS: $[M]^{+\cdot}$ calculated for $C_{15}H_9N$, 203.0735; found, 203.0734

Characterisation is in agreement with that reported in the literature.[10]

B(OPE3)*p*P

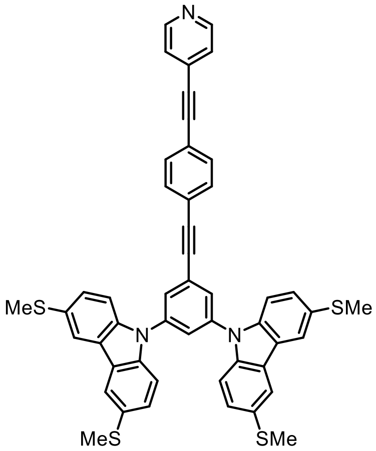

Prepared according to the general procedure for Sonogashira couplings using the following quantities of reagents: iodide **3** (160 mg, 0.223 mmol), alkyne **9** (50 mg, 0.25 mmol), CuI (2 mg, 0.01 mmol) and $Pd(PPh_3)_2Cl_2$ (8 mg, 0.01 mmol). Reaction time: 90 min. The crude product was a yellow solid (224 mg) which was purified by column chromatography (2 cm Ø, 50 mL SiO_2) eluting with DCM to remove a yellow band (impurity) then 20:1 DCM/acetone (ca. 210 mL), then 10:1 DCM/acetone to elute the near-colourless product band. After removing the solvent, a pale yellow solid (155 mg) was obtained. NMR spectra of this material were in agreement with what would be expected for **B(OPE3)*p*P**, although some pyridyl signals in the ^{1}H NMR spectrum were broader than analogous compounds. Elemental analysis showed significant deviation in the carbon content of the material (ca. 3.5 % below the expected value). These results led us to believe that the isolated material was at least partially a pyridinium salt. Attempts to purify the material by washing with sat. Na_2CO_3 then recrystallising from DCM/pentane were unsuccessful, affording similar spectra and elemental analysis values. However, by dissolving the material in DCM (50 mL) and washing with 2M $NaOH_{(aq)}$ (3 × 50 mL), then recrystallising the resulting material by dissolving it in minimal DCM and layering with pentane, it proved possible to isolate analytically pure **B(OPE3)*p*P** as a yellow solid (86 mg, 49%).

m.p.: 178–181 °C

^{1}H NMR (400 MHz, $CDCl_3$) δ 8.62 (d, *J* = 5.1 Hz, 2H), 8.09 (apparent t, *J* = 1.2 Hz, 4H), 7.81 (d, *J* = 2.0 Hz, 2H), 7.71 (t, *J* = 2.0 Hz, 1H), 7.56 (s, 4H), 7.47 (apparent d, *J* = 1.2 Hz, 8H), 7.42 – 7.36 (m, 2H), 2.59 (s, 12H).

^{13}C NMR (101 MHz, $CDCl_3$) δ 149.9, 139.5, 139.4, 132.1, 132.0, 131.2, 129.8, 128.6, 128.2, 126.8, 125.7, 125.0, 124.0, 123.2, 122.8, 121.0, 110.4, 93.4, 91.8, 89.7, 88.9, 18.7.

MS: ASAP: 794.2 $[M+H]^+$, 793.2 $[M]^{+\cdot}$

HRMS: $[M]^{+\cdot}$ calculated for $C_{49}H_{35}N_3S_4$, 793.1714; found, 793.1699

elem.: Anal. Calcd for $C_{49}H_{35}N_3S_4$: C, 74.12; H, 4.44 ; N, 5.29. Found: C, 74.01; H, 4.36; N, 5.22.

3,5-bis(*N*-Carbazolyl)iodobenzene, **5**

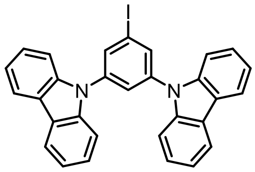

Carbazole (349 mg, 2.09 mmol, 2.5 eq.) was dissolved in anhydrous DMF (10 mL) in a dry flask under argon at RT. Caesium carbonate (952 mg, 2.92 mmol, 3.5 eq.) was added (solution turns yellow) and the mixture was stirred for 15 min at RT. 3,5-Difluoroiodobenzene (0.1 mL, 0.835 mmol, 1.0 eq.) was then added and the mixture set to heat at 150 °C. After 17 h, TLC (2:1 hexane/DCM) indicated that the reaction was complete. The mixture was allowed to cool to RT then treated with deionised water (50 mL), affording a yellow precipitate which was isolated by filtration and washed thoroughly with deionised water. The precipitate was then dissolved in DCM (100 mL) and washed with deionised water (2 × 75 mL) and brine (75 mL). The organic layer was dried ($MgSO_4$) before removing the solvent *in vacuo*, affording a fluffy yellow solid (512 mg). The crude product was purified by column chromatography (5 cm Ø, 250 mL SiO_2, eluent: 2:1 hexane/DCM). After removal of solvent *in vacuo*, **5** was obtained as a white solid of sufficient purity for the subsequent reaction (312 mg, 70%).

m.p.: 193–194.5 °C

^{1}H NMR (400 MHz, $CDCl_3$) δ 8.15 (ddd, *J* = 7.6, 1.2, 0.9 Hz, 4H), 8.06 (d, *J* = 1.9 Hz, 2H), 7.82 (t, *J* = 1.9 Hz, 1H), 7.54 (ddd, *J* = 8.3, 1.2, 0.9 Hz, 4H), 7.46 (ddd, *J* = 8.3, 7.1, 1.2 Hz, 4H), 7.33 (td, *J* = 7.6, 7.1, 1.2 Hz, 4H).

^{13}C NMR (101 MHz, $CDCl_3$) δ 140.4, 134.7, 126.5, 124.9, 123.9, 120.9, 120.7, 109.7, 95.2.*

MS: ASAP: 535.0 $[M+H]^+$, 534.1 $[M]^{+\cdot}$, 409.2 $[M\text{-}I+2H]^+$

HRMS: $[M+H]^+$ calculated for $C_{30}H_{20}IN_2$, 535.0671; found, 535.0653

OPE2 Model System, **1**

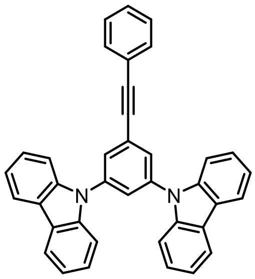

Prepared according to the general procedure for Sonogashira couplings using the following quantities of reagents: iodide **5** (175 mg, 0.327 mmol), phenylacetylene (40 µL, 0.364 mmol), CuI (3 mg, 0.015 mmol) and $Pd(PPh_3)_2Cl_2$ (11 mg, 0.016 mmol). Reaction time: 120 min. The crude product was a yellow solid (243 mg) which was purified by column chromatography (3 cm Ø, 150 mL SiO_2) eluent: 5:1 hexane/DCM). Two yellow bands (both impurities) eluted before **1**, which was isolated as an off-white solid (160 mg), which was recrystallised from EtOH to afford **1** as shiny white needles (117 mg, 70%). These crystals were suitable for X-ray crystallography.

m.p.: 175–176 °C

^{1}H NMR (400 MHz, $CDCl_3$) δ 8.17 (dd, *J* = 7.7, 1.2, 0.7 Hz, 4H), 7.87 (d, *J* = 2.0 Hz, 2H), 7.80 (t, *J* = 2.0 Hz, 1H), 7.61 – 7.55 (m, 6H), 7.48 (ddd, *J* = 8.2, 7.2, 1.2 Hz, 4H), 7.41 – 7.36 (m, 3H), 7.34 (ddd, *J* = 7.7, 7.2, 1.0 Hz, 5H).

^{13}C NMR (101 MHz, $CDCl_3$) δ 140.6, 139.7, 131.9, 129.1, 128.8, 128.6, 127.0, 126.4, 125.4, 123.8, 122.6, 120.7, 120.6, 109.8, 92.1, 87.7.

MS: ASAP: 509.2 $[M+H]^+$, 508.2 $[M]^{+\cdot}$

HRMS: $[M]^{+\cdot}$ calculated for $C_{38}H_{24}N_2$, 508.1939; found, 508.1937

elem.: Anal. Calcd for $C_{38}H_{24}N_2$: C, 89.74; H, 4.76; N, 5.51. Found: C, 89.42; H, 4.65; N, 5.59.

* Nine aromatic signals are seen rather than the expected ten – two signals are presumed to overlap.

4-(Acetylthio)iodobenzene, **8**

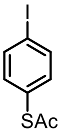

1,4-Diiodobenzene (18.05 g, 54.7 mmol, 2.5 eq.) was dissolved in toluene (250 mL) in a dry flask under argon and degassed (argon, 45 min) before CuI (417 mg, 2.19 mmol, 0.1 eq.), 1,10-phenanthroline (789 mg, 4.38 mmol, 0.2 eq.) and KSAc (2.50 g, 21.9 mmol, 1 eq.) were added and the mixture was heated at reflux for 2.5 days. The reaction was cooled to RT then diluted with diethyl ether (200 mL) and extracted with deionised water (250 mL) then saturated $NH_4Cl_{(aq)}$ (3 × 100 mL). The organic phase was dried ($MgSO_4$) and the solvent was removed *in vacuo* to give a yellow solid (ca. 16.4 g), which was purified by column chromatography (7 cm Ø, 550 mL SiO_2, eluent: 2:1 hexane/DCM (ca. 600 mL, then 1:1 (ca. 600 mL), then 1:2). Excess 1,4-diiodobenzene (11.6 g, 35.2 mmol) eluted first* followed by **8**, which was isolated as an off-white solid (4.35 g, 71%).

m.p.: 55–56 °C (lit.[11] 56–57 °C)

^{1}H NMR (400 MHz, $CDCl_3$) δ 7.77 – 7.71 (m†, 2H), 7.16 – 7.10 (m‡, 2H), 2.43 (s, 3H).

^{13}C NMR (101 MHz, $CDCl_3$) δ 193.4, 138.5, 136.1, 127.9, 96.1, 30.4.

MS: ASAP: 320.0 $[M+Ac]^+$, 278.9 $[M+H]^+$

HRMS: $[M]^{+\cdot}$ calculated for C_8H_8IOS, 278.9341; found, 278.9345

SB

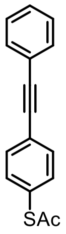

Prepared according to the general procedure for Sonogashira couplings using the following quantities of reagents: iodide **8** (345 mg, 1.24 mmol), phenylacetylene (0.15 mL, 1.37 mmol), CuI (12 mg, 0.063 mmol) and $Pd(PPh_3)_2Cl_2$ (44 mg, 0.063 mmol). Reaction time: 18 h. The crude product was a brown solid (412 mg) which was dissolved in DCM and evaporated onto celite (20 mL) then purified by column chromatography (4 cm Ø, 150 mL SiO_2, eluent: 2:1 hexane/DCM). Removal of the solvent *in vacuo* afforded **SB** as a white solid (228 mg, 73%).

m.p.: 102–103 °C (lit.[10] 99–101 °C)

^{1}H NMR (400 MHz, $CDCl_3$) δ 7.58 – 7.51 (m, 4H), 7.42 – 7.38 (m, 2H), 7.38 – 7.33 (m, 3H), 2.44 (s, 3H).

^{13}C NMR (101 MHz, $CDCl_3$) δ 193.6, 134.4, 132.3, 131.8, 128.7, 128.5, 128.2, 124.7, 123.1, 91.2, 88.8, 30.4.

MS: ASAP: 253.1 $[M+H]^+$, 252.1 $[M]^{+\cdot}$, 211.1 $[M-Ac+2H]^+$

HRMS: $[M+H]^+$ calculated for $C_{16}H_{13}OS$, 253.0687; found, 253.0685

elem.: Anal. Calcd for $C_{16}H_{12}OS$: C, 76.16; H, 4.79. Found: C, 76.02; H, 4.78.

Characterisation is in agreement with that reported in the literature.[10]

* 64% of the starting material used. This material was of good purity and suitable for re-use.
† This signal resembles a doublet with *J* = 8.5, but additional higher-order features are present
‡ This signal resembles a doublet with *J* = 8.5, but additional higher-order features are present

S*p*P

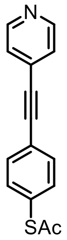

Prepared according to the general procedure for Sonogashira couplings using the following quantities of reagents: iodide **8** (345 mg, 1.24 mmol), 4-ethynylpyridine (141 mg, 1.37 mmol), CuI (12 mg, 0.063 mmol) and $Pd(PPh_3)_2Cl_2$ (44 mg, 0.063 mmol). Reaction time: 18 h. The crude product was a yellow-brown solid (403 mg) which was purified by column chromatography (4 cm Ø, 150 mL SiO_2, eluent: DCM, then 10:1 DCM/acetone). Removal of the solvent *in vacuo* afforded **S*p*P** as a cream-coloured solid which darkened to brown over a few weeks even if stored in a refrigerator (210 mg, 67%).

m.p.: 86.5–87.5 °C (lit.[12] 85–86 °C)

^{1}H NMR (400 MHz, $CDCl_3$) δ 8.62 (d, *J* = 5.1 Hz, 2H), 7.60 – 7.56 (m*, 2H), 7.45 – 7.41 (m†, 2H), 7.40 – 7.37 (m‡, 2H), 2.45 (s, 3H).

^{13}C NMR (101 MHz, $CDCl_3$) δ 193.3, 150.0, 134.4, 132.5, 131.2, 129.4, 125.7, 123.4, 93.2, 88.2, 30.5.

MS: ASAP: 254.1 $[M+H]^+$, 212.1 $[M-Ac+2H]^+$

HRMS: $[M+H]^+$ calculated for $C_{15}H_{12}NOS$, 254.0640; found, 254.0652

elem.: Anal. Calcd for $C_{15}H_{11}NOS$: C, 71.12; H, 4.38; N, 5.53. Found: C, 70.71; H, 4.36; N, 5.43.

Characterisation is in agreement with that reported in the literature.[12]

S*m*P

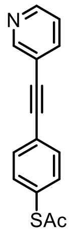

Prepared according to the general procedure for Sonogashira couplings using the following quantities of reagents: iodide **8** (345 mg, 1.24 mmol), 3-ethynylpyridine (141 mg, 1.37 mmol), CuI (12 mg, 0.063 mmol) and $Pd(PPh_3)_2Cl_2$ (44 mg, 0.063 mmol). Reaction time: 18 h. The crude product was a yellow-brown solid (409 mg) which was purified by column chromatography (4 cm Ø, 150 mL SiO_2, eluent: DCM, then 10:1 DCM/acetone). Removal of the solvent *in vacuo* afforded **S*m*P** as a cream-coloured solid (238 mg, 76%).

m.p.: 92.5–94 °C (lit.[13] 95–98 °C)

^{1}H NMR (400 MHz, $CDCl_3$) δ 8.77 (bs§, 1H), 8.57 (dd, *J* = 4.9, 1.6 Hz, 1H), 7.81 (ddd, *J* = 7.9, 2.1, 1.6 Hz, 1H), 7.59 – 7.55 (m, 2H), 7.44 – 7.39 (m, 2H), 7.30 (ddd, *J* = 7.9, 4.9, 0.9 Hz, 2H), 2.44 (s, 3H).

^{13}C NMR (101 MHz, $CDCl_3$) δ 193.4, 152.4, 149.0, 138.7, 134.4, 132.4, 128.9, 123.9, 123.2, 120.3, 92.0, 87.7, 30.5.

MS: ASAP: 254.1 $[M+H]^+$, 212.1 $[M-Ac+2H]^+$

HRMS: $[M+H]^+$ calculated for $C_{15}H_{12}NOS$, 254.0640; found, 254.0642

elem.: Anal. Calcd for $C_{15}H_{11}NOS$: C, 71.12; H, 4.38; N, 5.53. Found: C, 71.07; H, 4.39; N, 5.46.

---

* This signal resembles a doublet with *J* = 8.5, but additional higher-order features are present
† This signal resembles a doublet with *J* = 8.5, but additional higher-order features are present
‡ This signal resembles a doublet with *J* = 5.1, but additional higher-order features are present
§ Expected couplings of *J* = 2.1 and 0.9 (based on analysis of other peaks) are not clearly resolved at this frequency.

Characterisation is in agreement with that reported in the literature.[13]

1.3 ¹H NMR Spectra

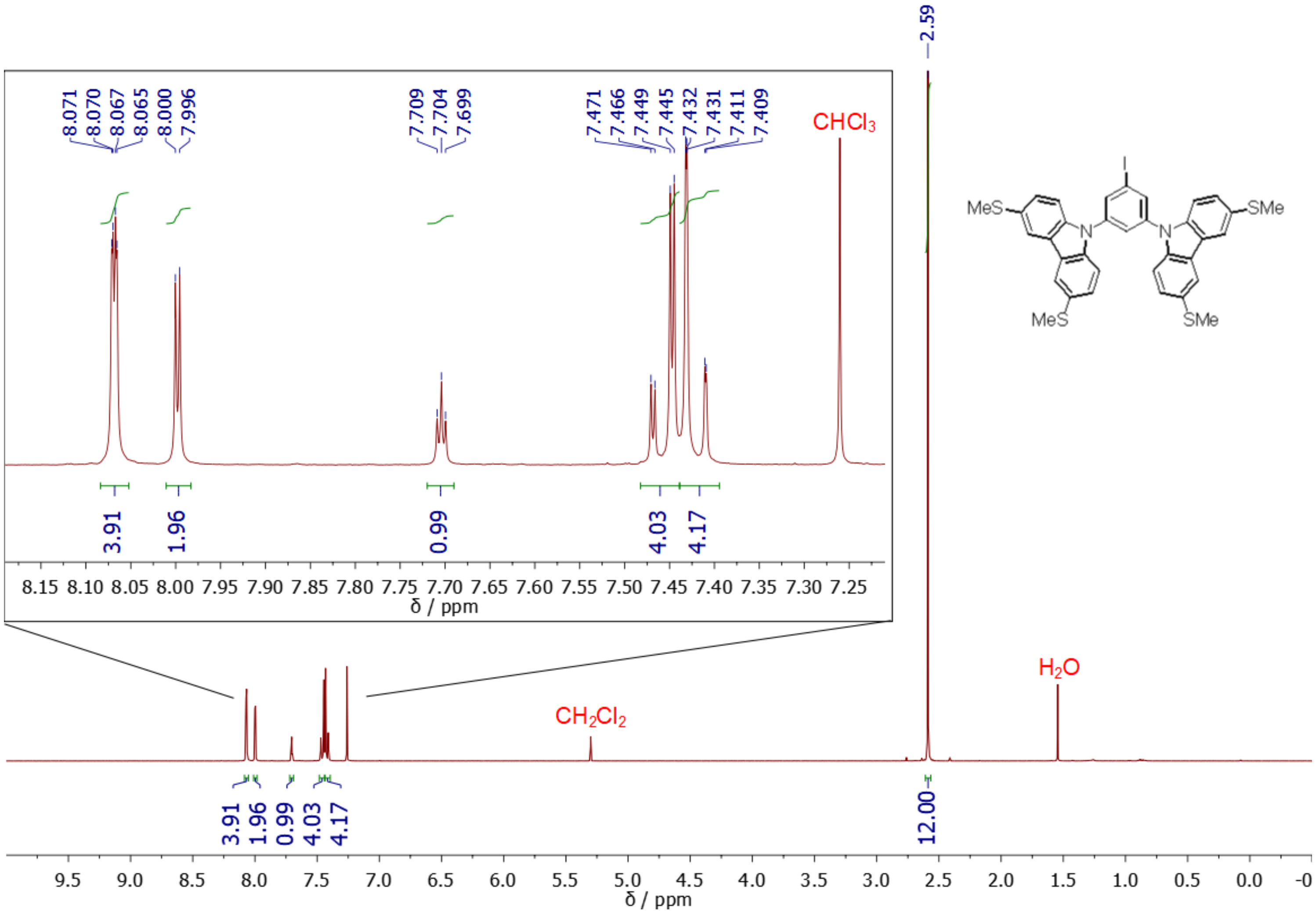

Figure S1.01. ¹H NMR spectrum of **3** in $CDCl_3$.

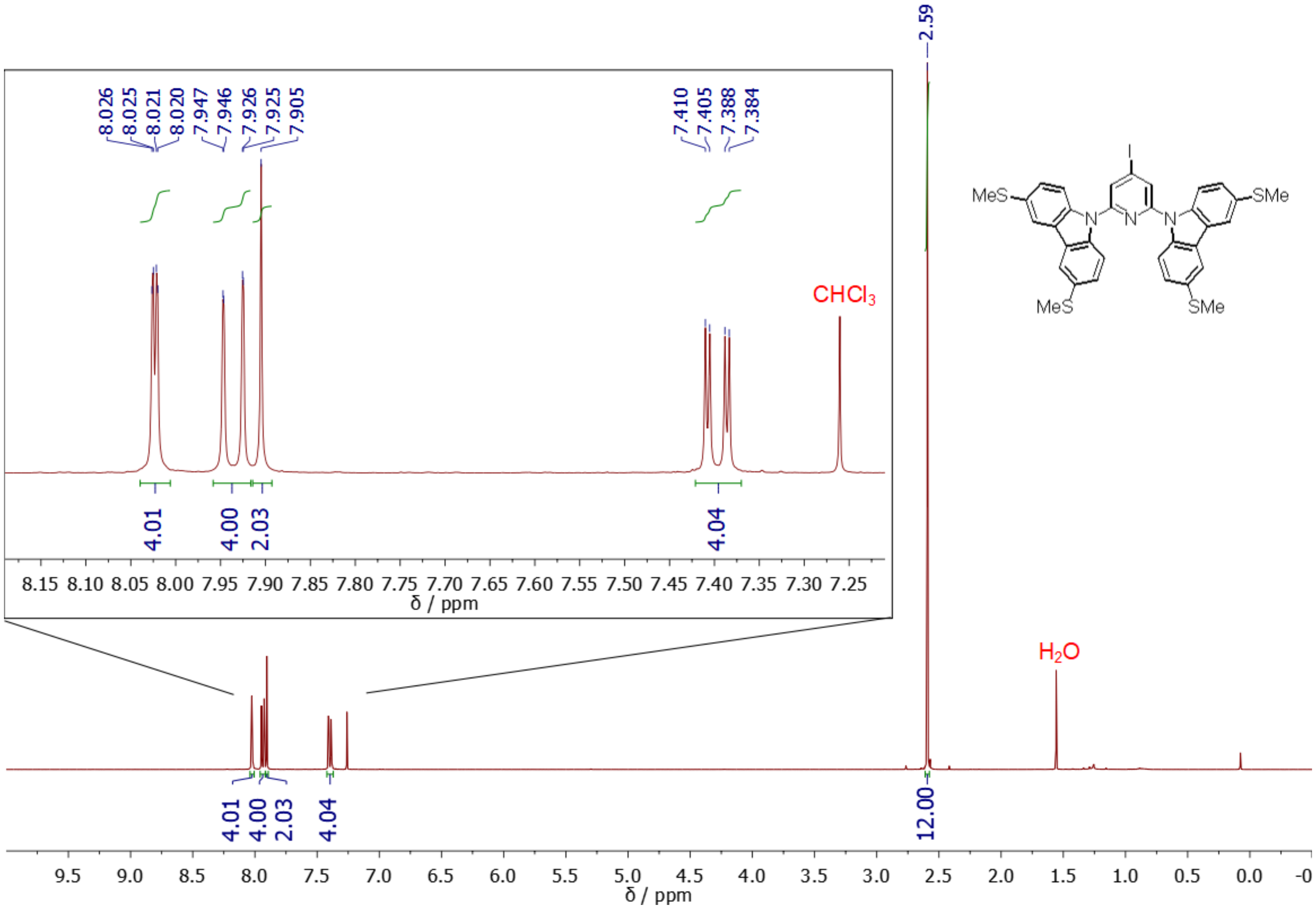

Figure S1.02. ^{1}H NMR spectrum of **4** in $CDCl_3$.

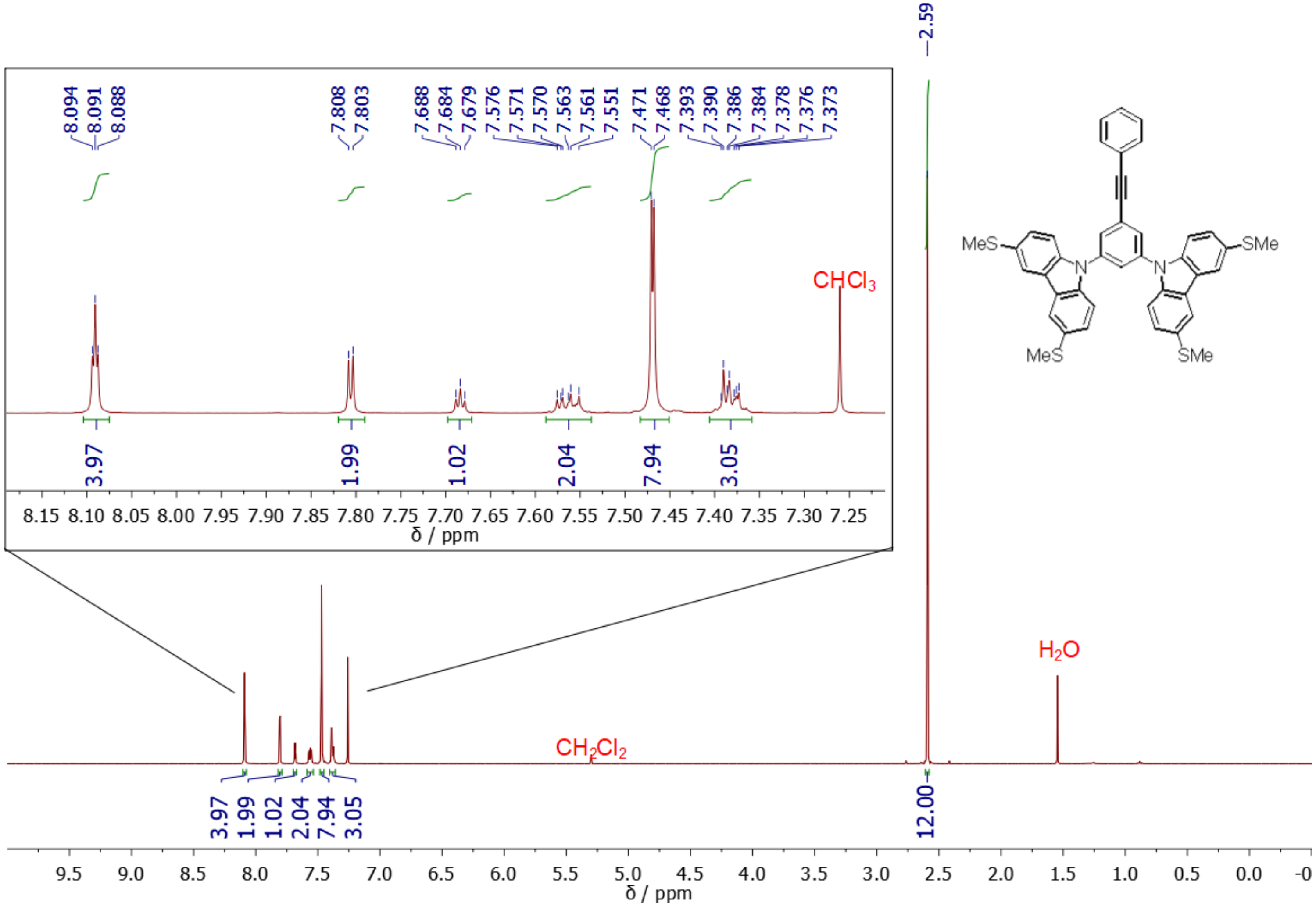

Figure S1.03. ^{1}H NMR spectrum of **BB** in $CDCl_3$.

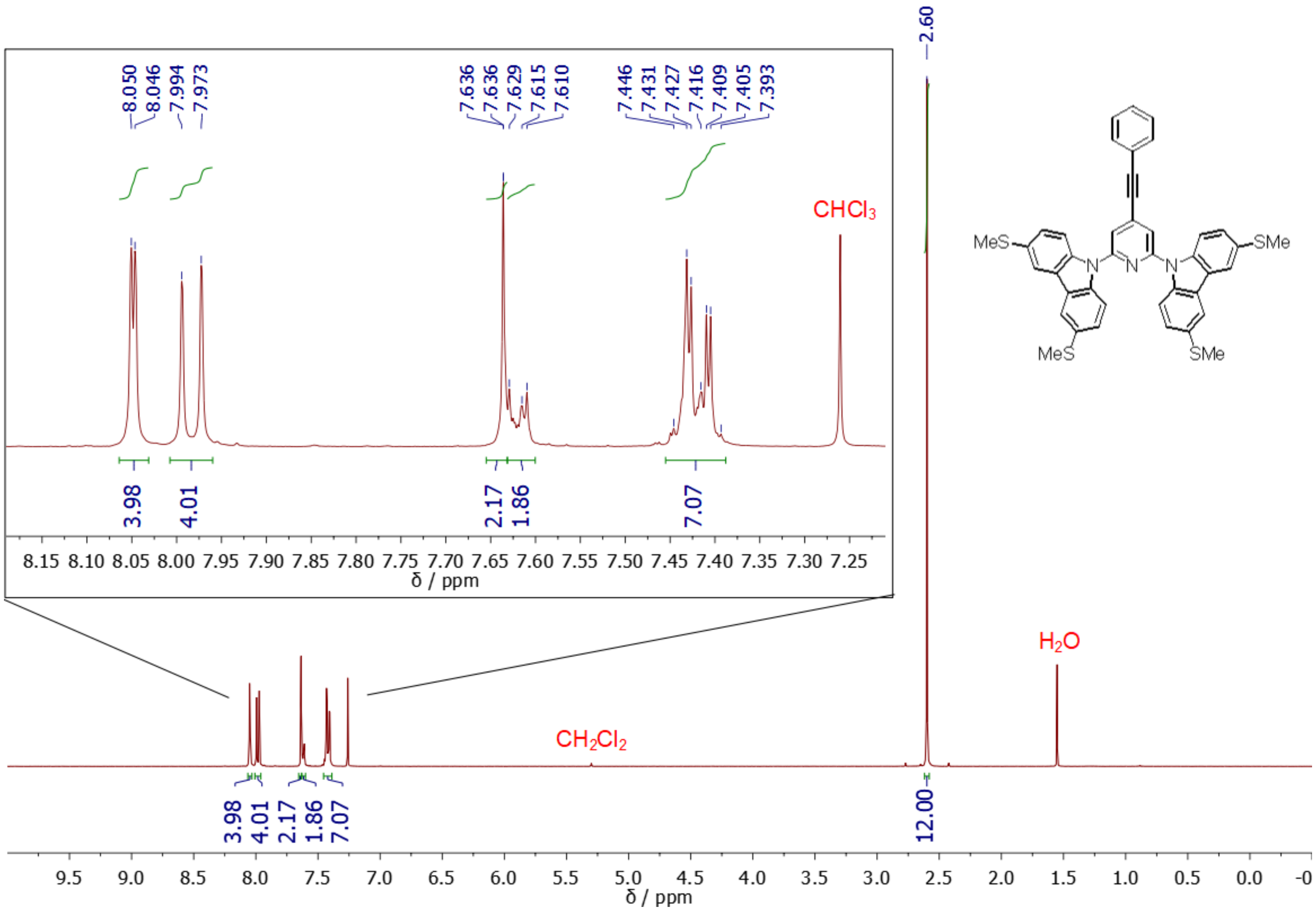

Figure S1.04. ^{1}H NMR spectrum of **PB** in $CDCl_3$.

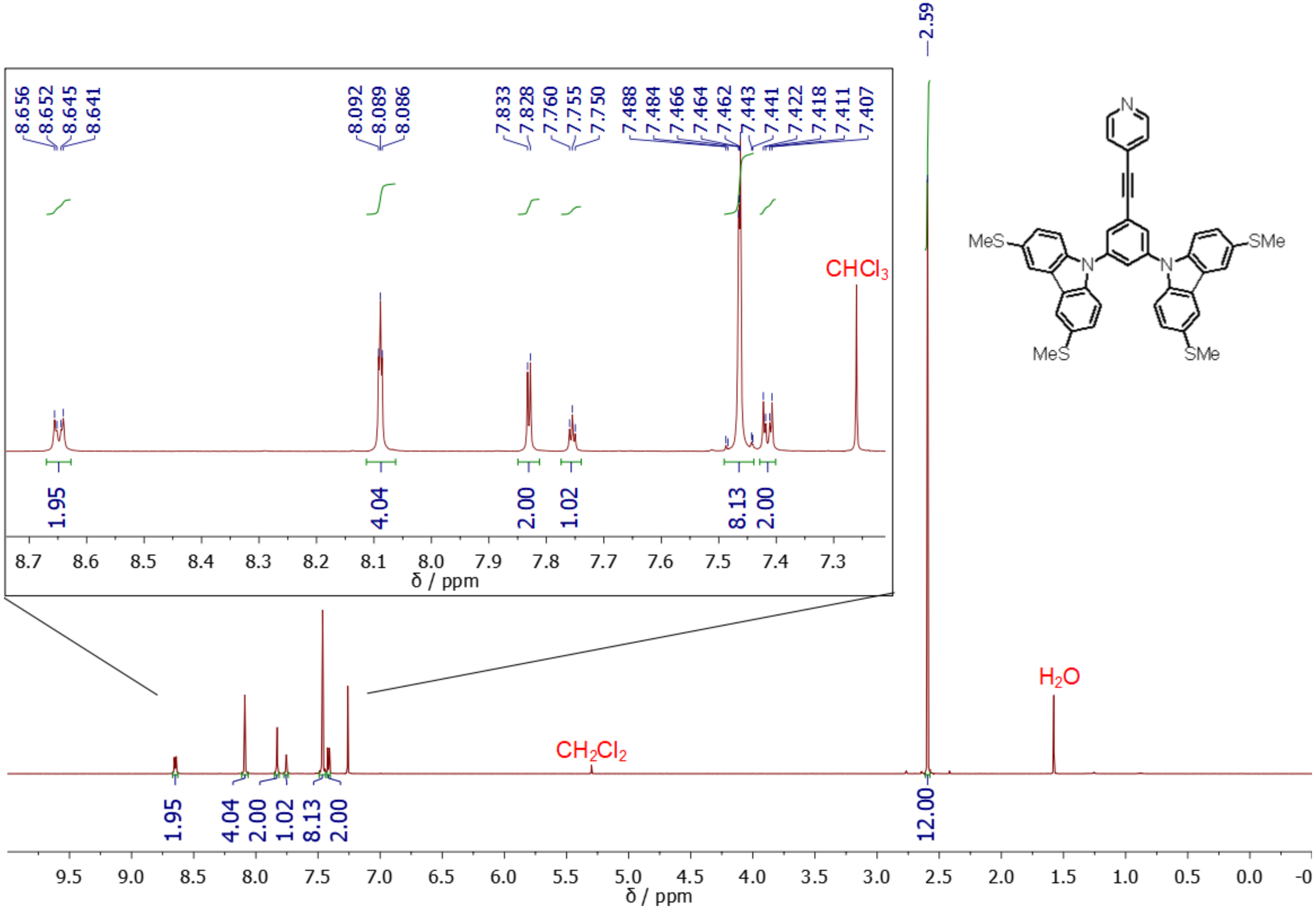

Figure S1.05. ^{1}H NMR spectrum of **B*p*P** in $CDCl_3$.

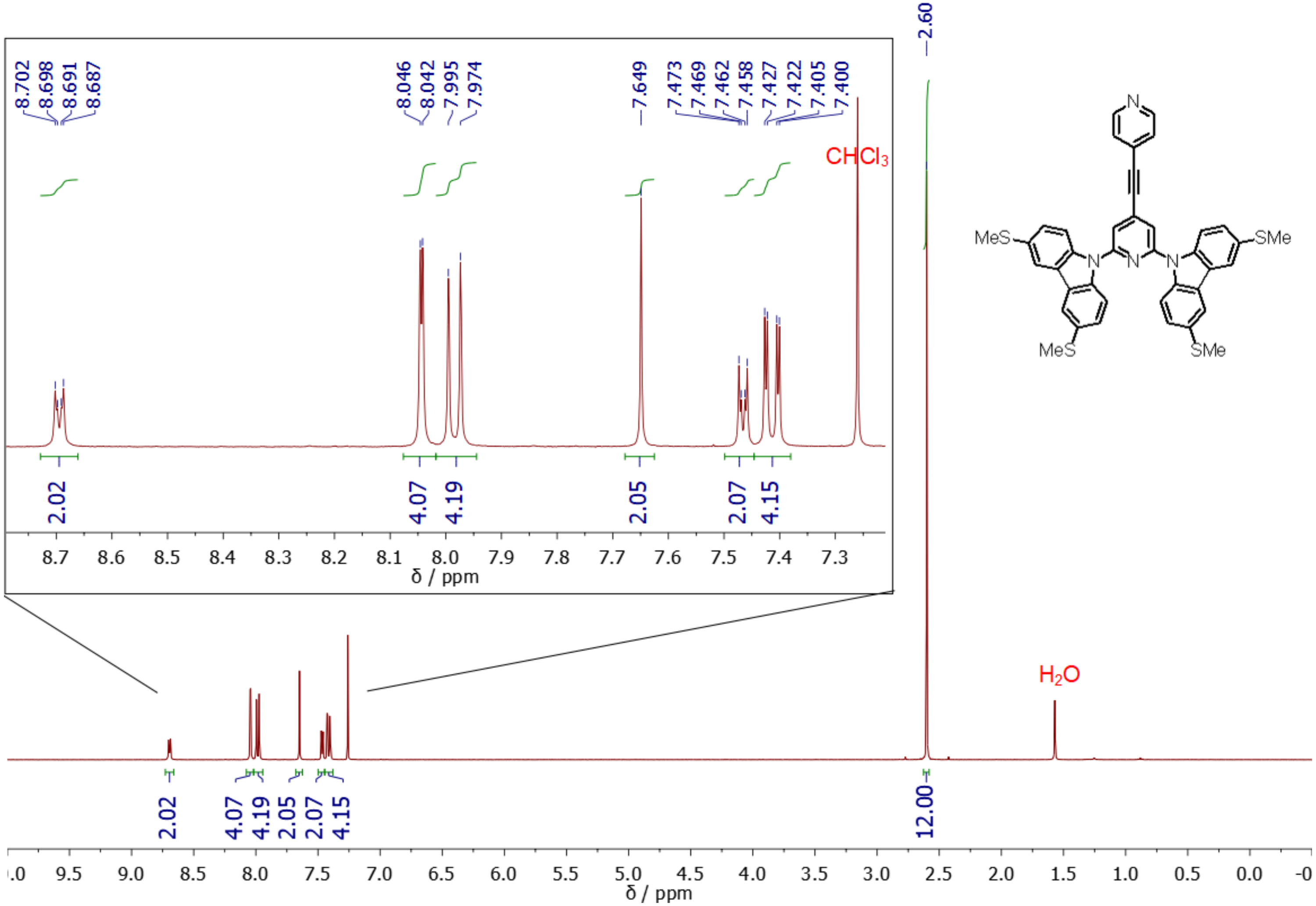

Figure S1.06. ^{1}H NMR spectrum of **P*p*P** in $CDCl_3$.

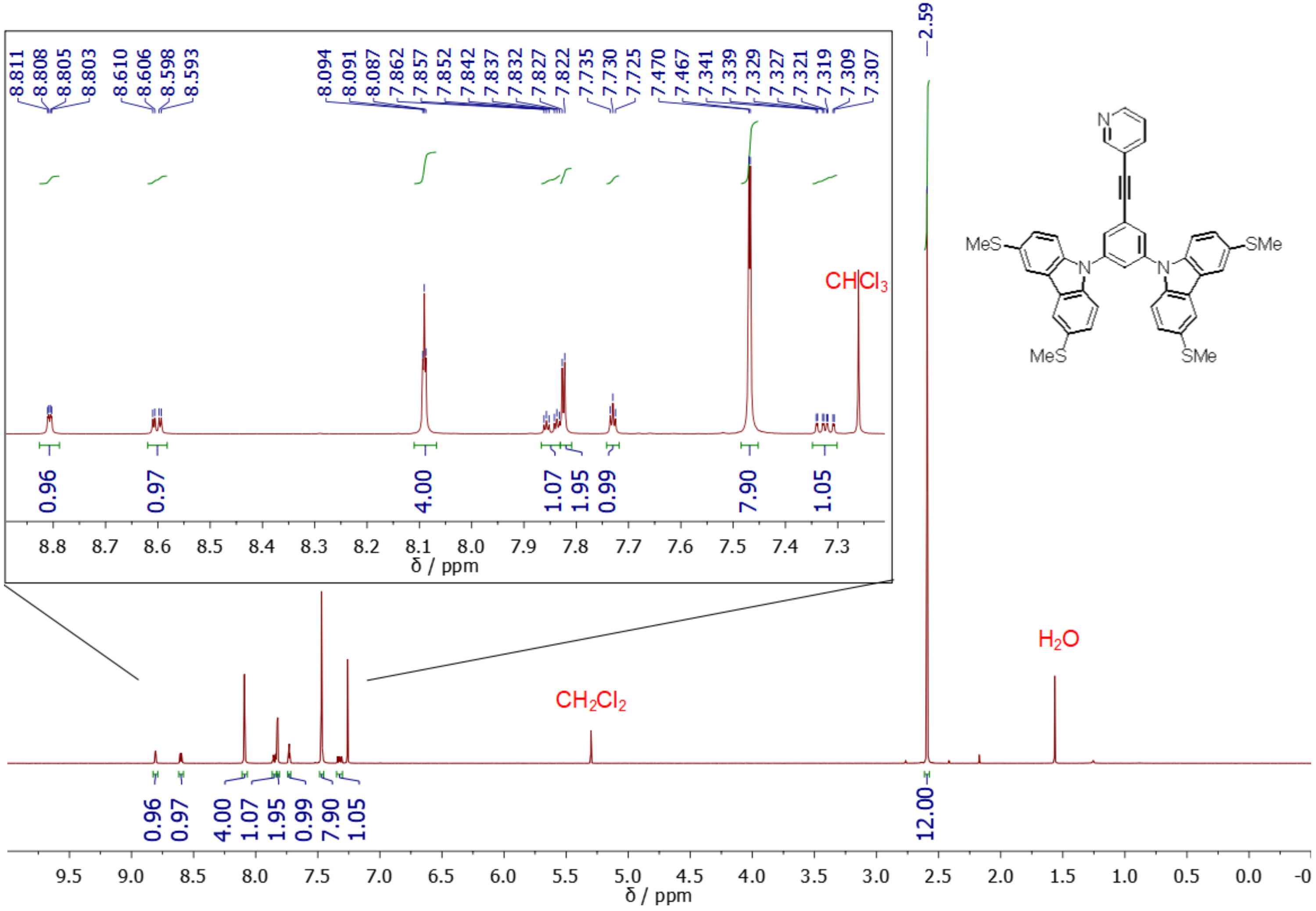

Figure S1.07. ^{1}H NMR spectrum of **B*m*P** in $CDCl_3$.

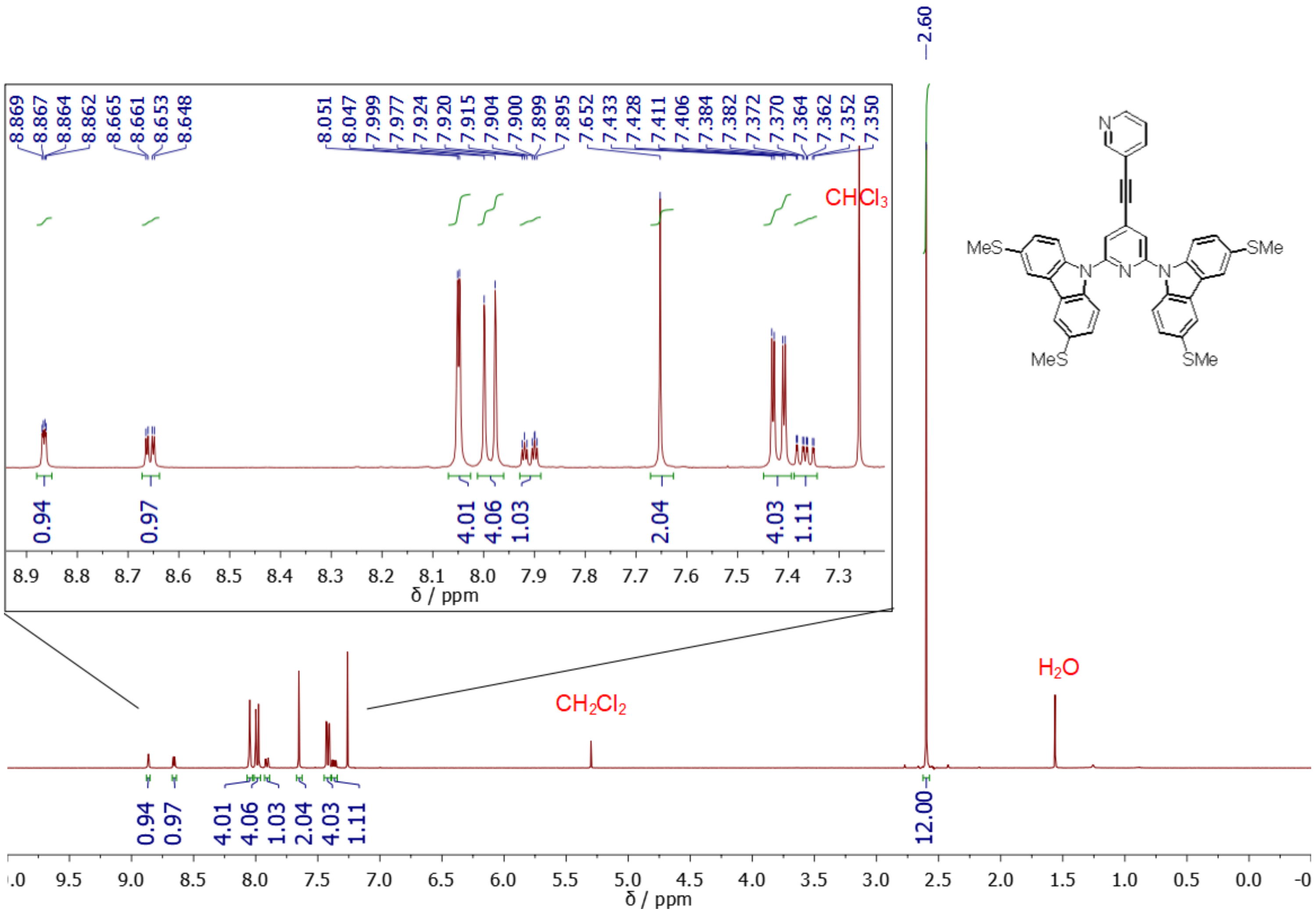

Figure S1.08. ^{1}H NMR spectrum of ***PmP*** in $CDCl_3$.

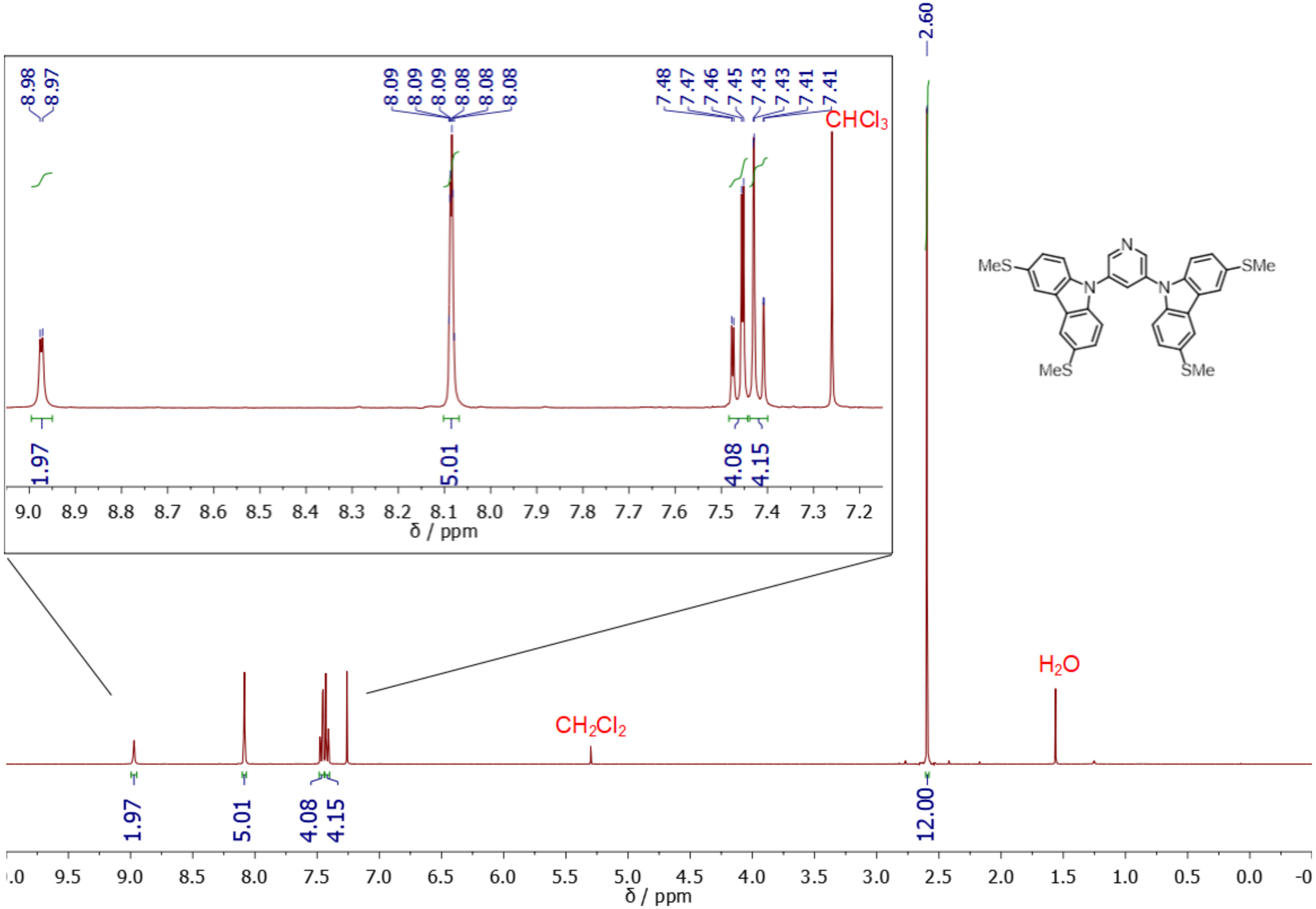

Figure S1.09. ^{1}H NMR spectrum of **B(OPE1)*p*P** in $CDCl_3$.

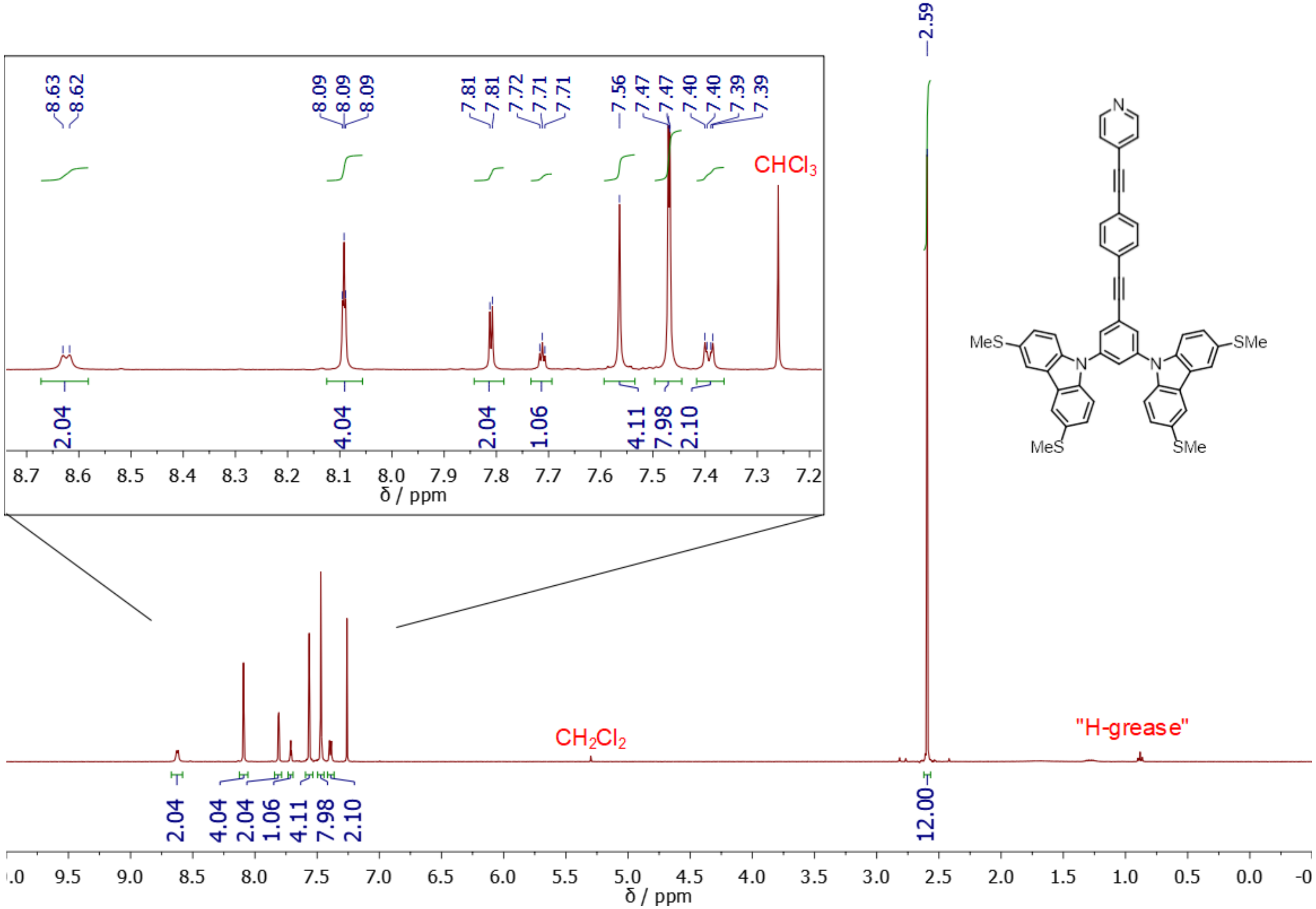

Figure S1.10. ^{1}H NMR spectrum of **B(OPE3)*p*P** in $CDCl_3$.

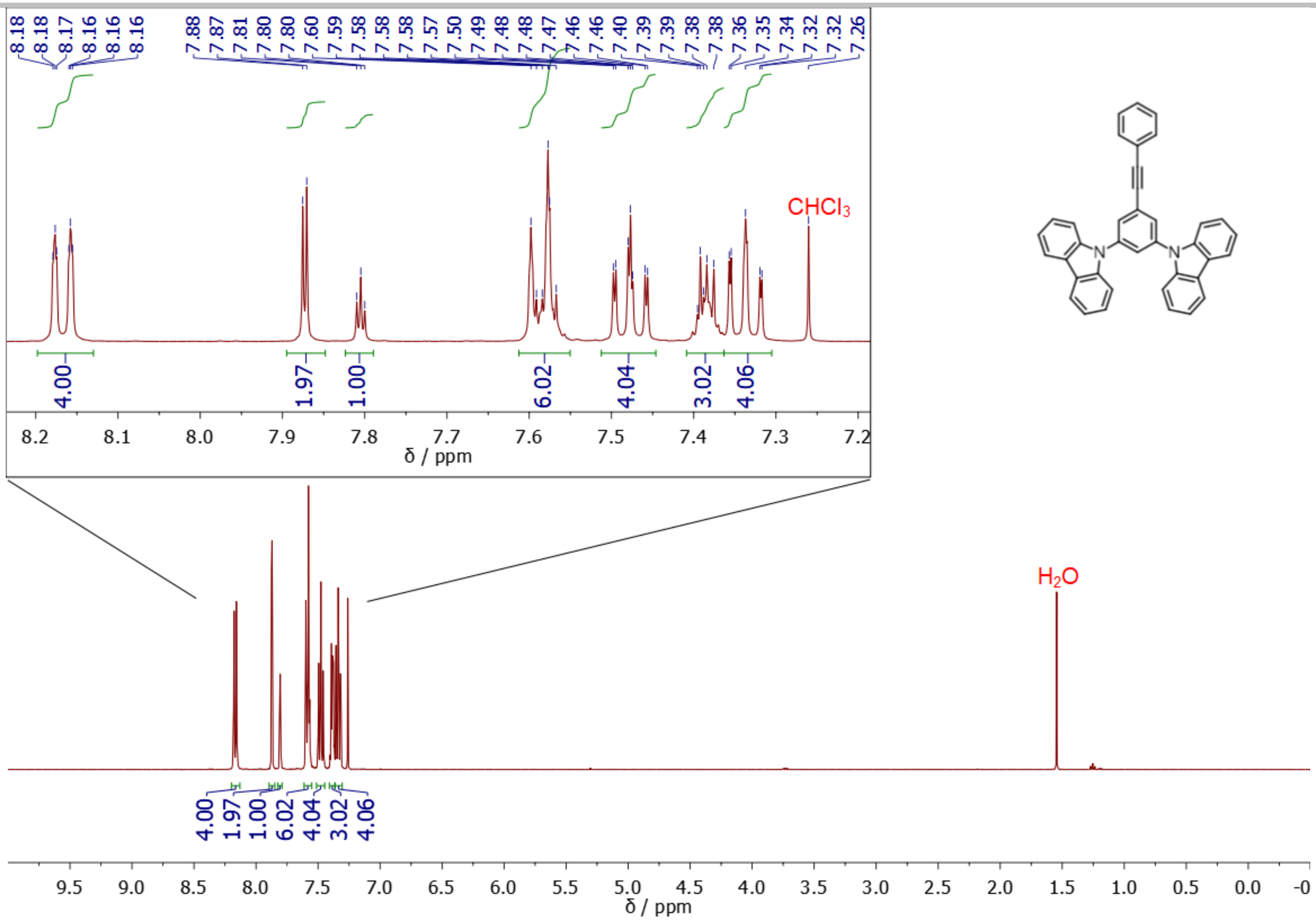

Figure S1.11. ^{1}H NMR spectrum of **1** in $CDCl_3$.

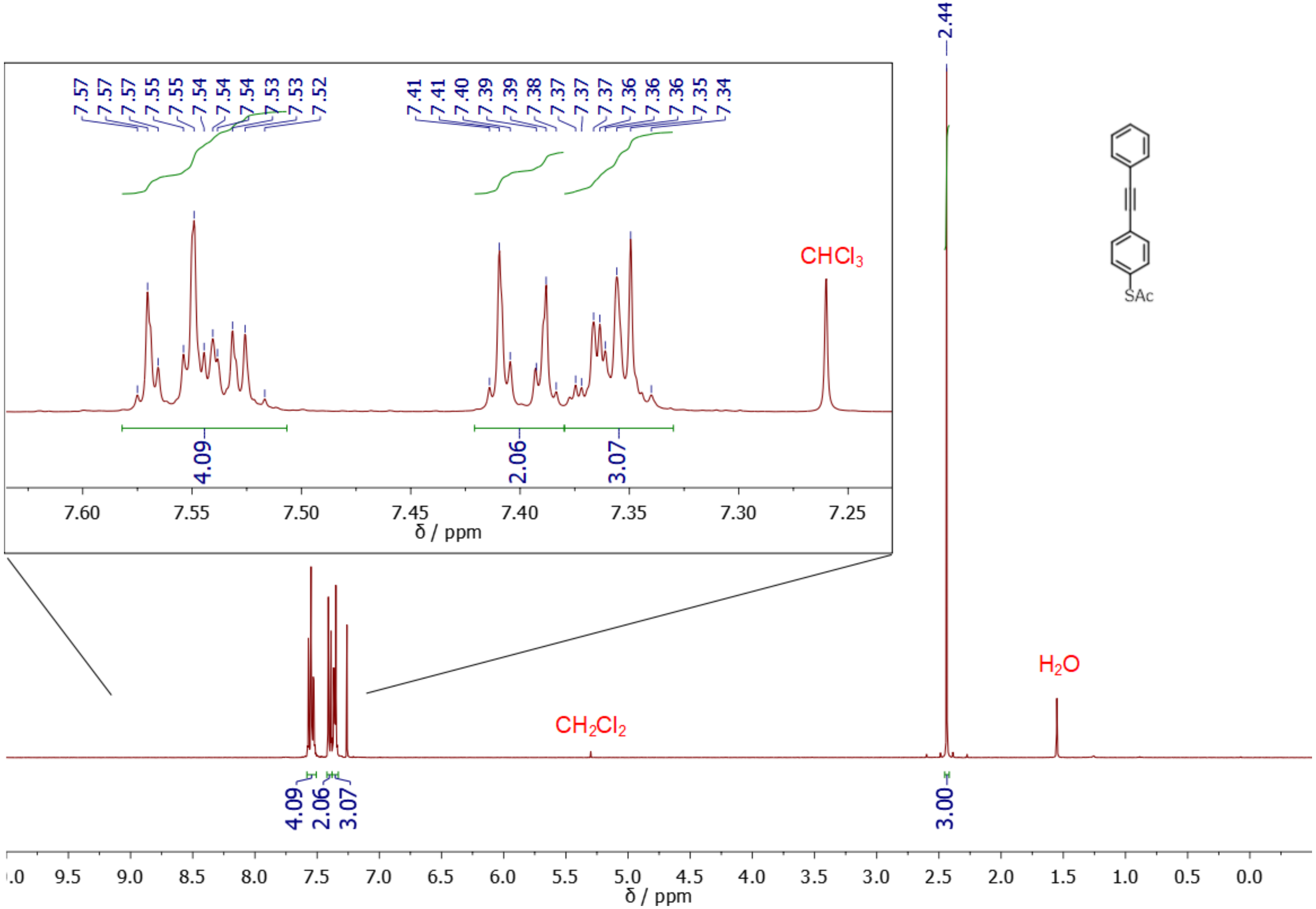

Figure S1.12. ^1H NMR spectrum of **SB** in $CDCl_3$.

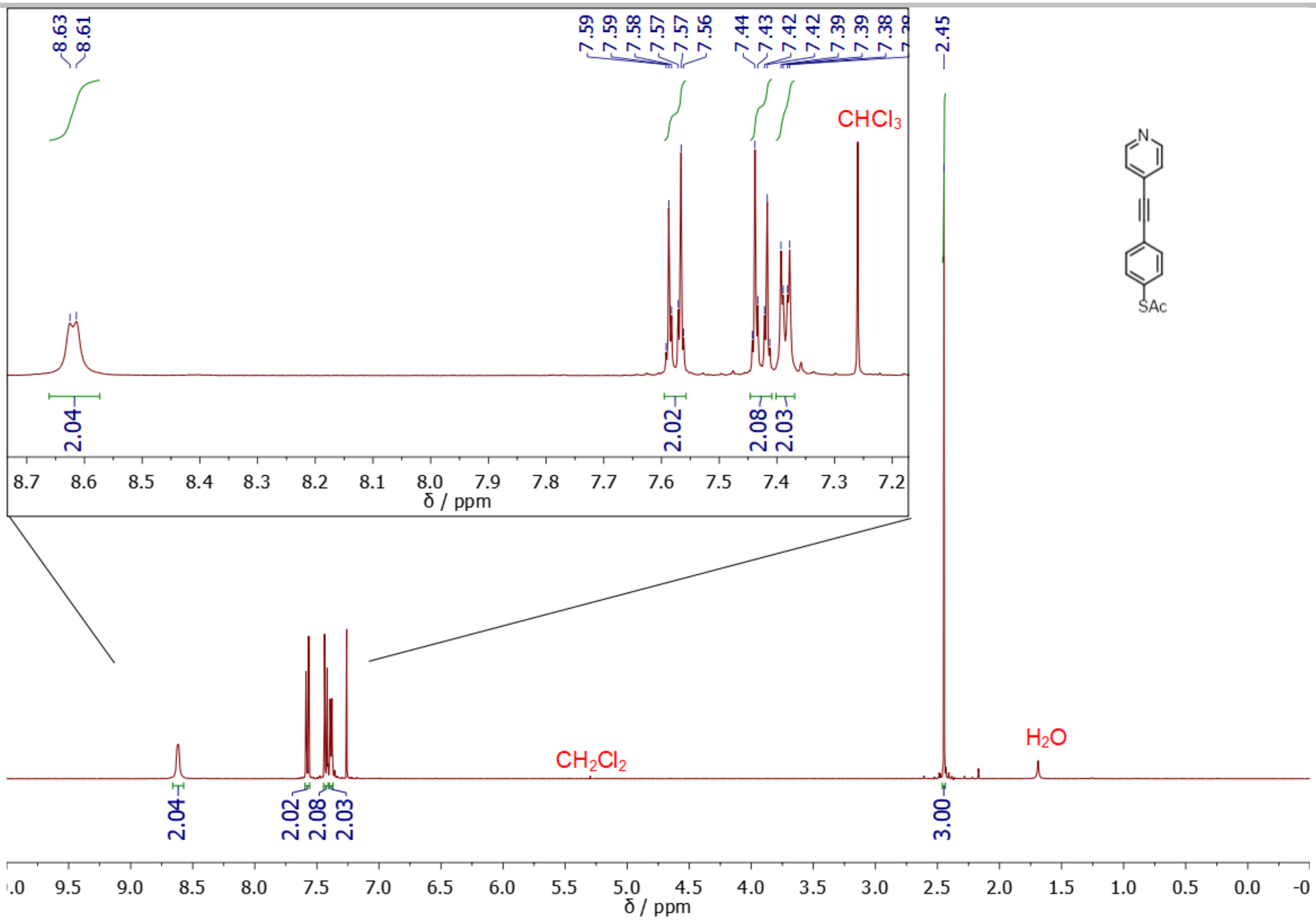

Figure S1.13. ^{1}H NMR spectrum of **S*p*P** in $CDCl_3$.

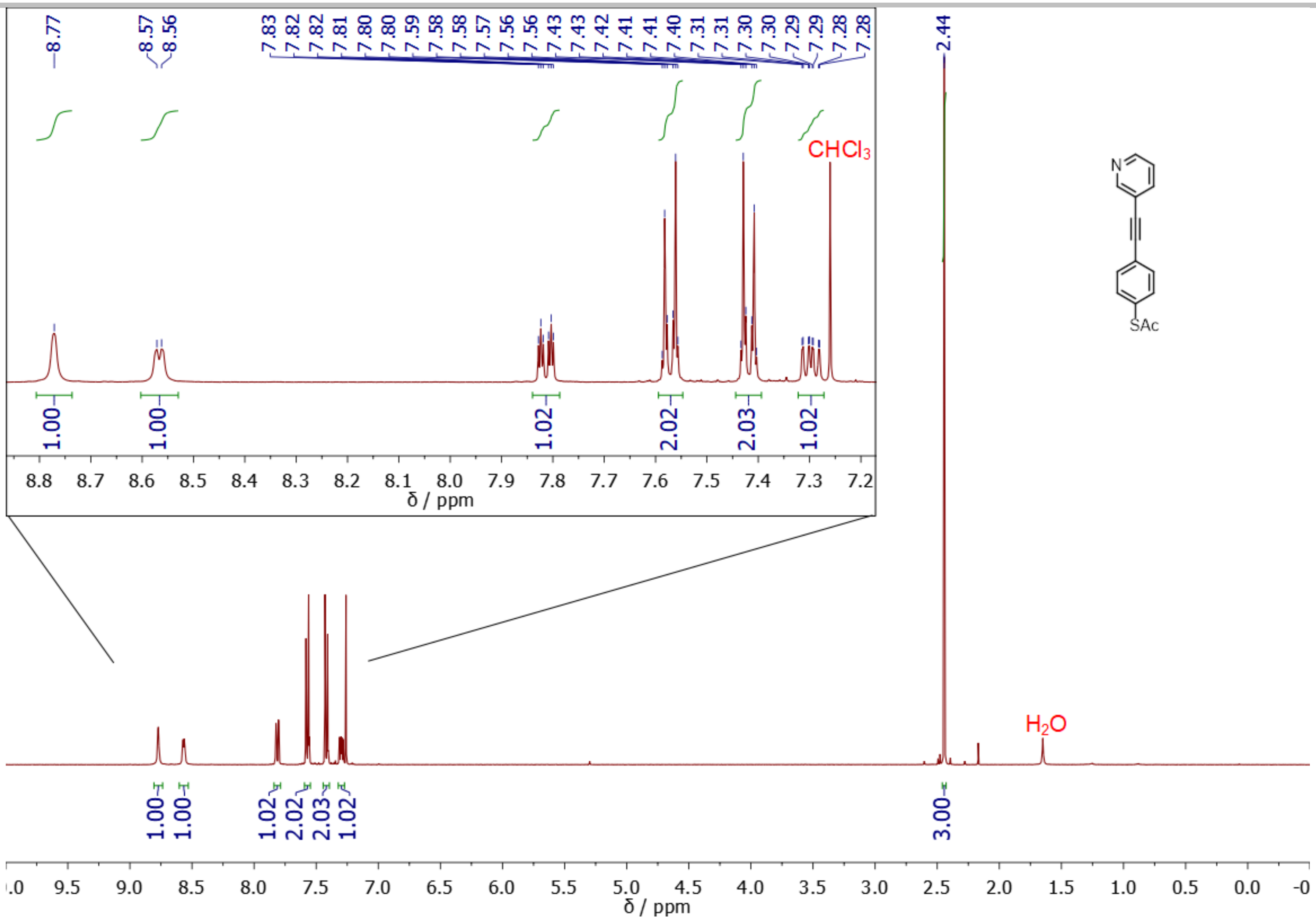

Figure S1.14. ^{1}H NMR spectrum of ***SmP*** in $CDCl_3$.

1.4 ^{13}C NMR Spectra

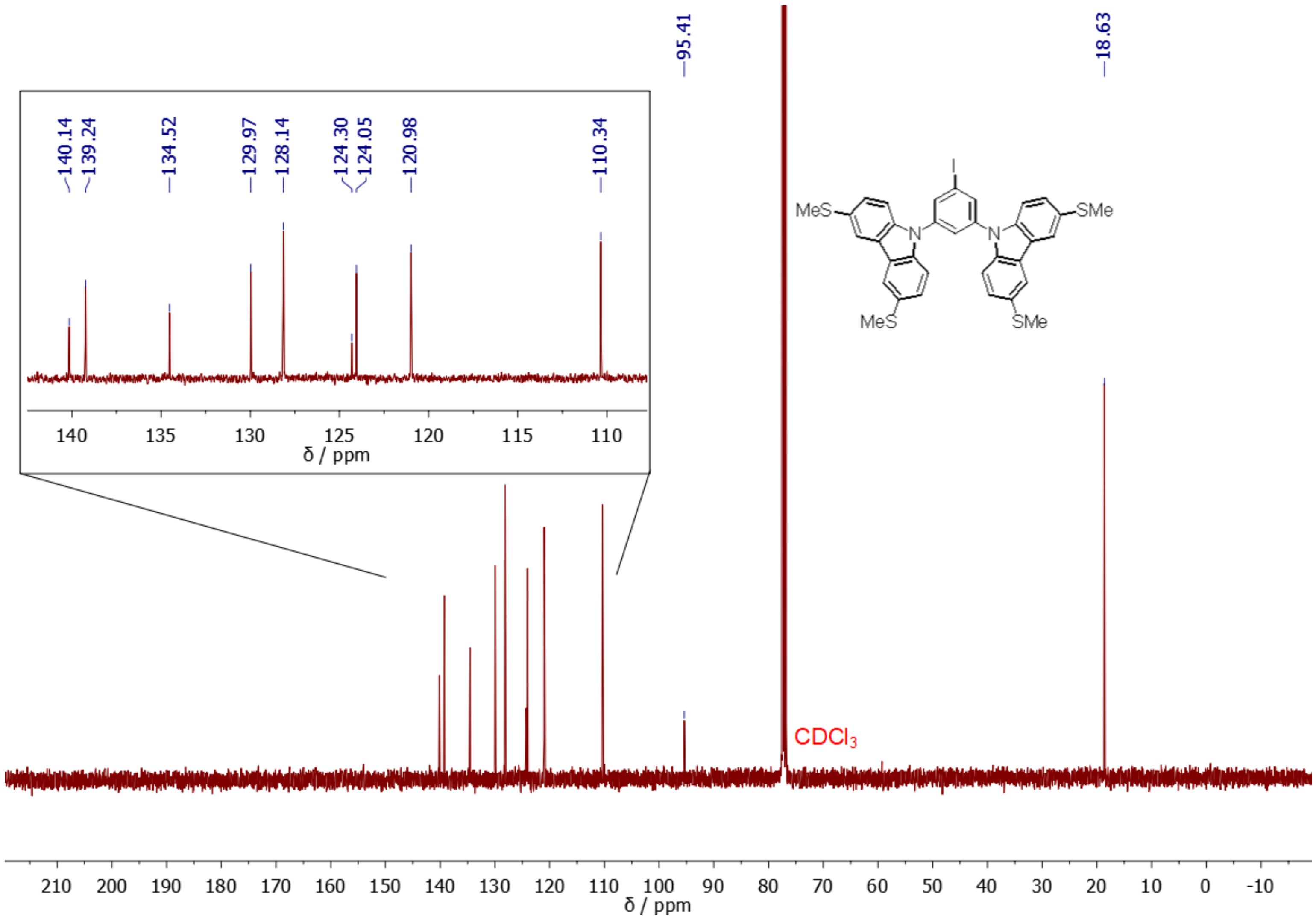

Figure S1.15. ^{13}C NMR spectrum of **3** in $CDCl_3$.

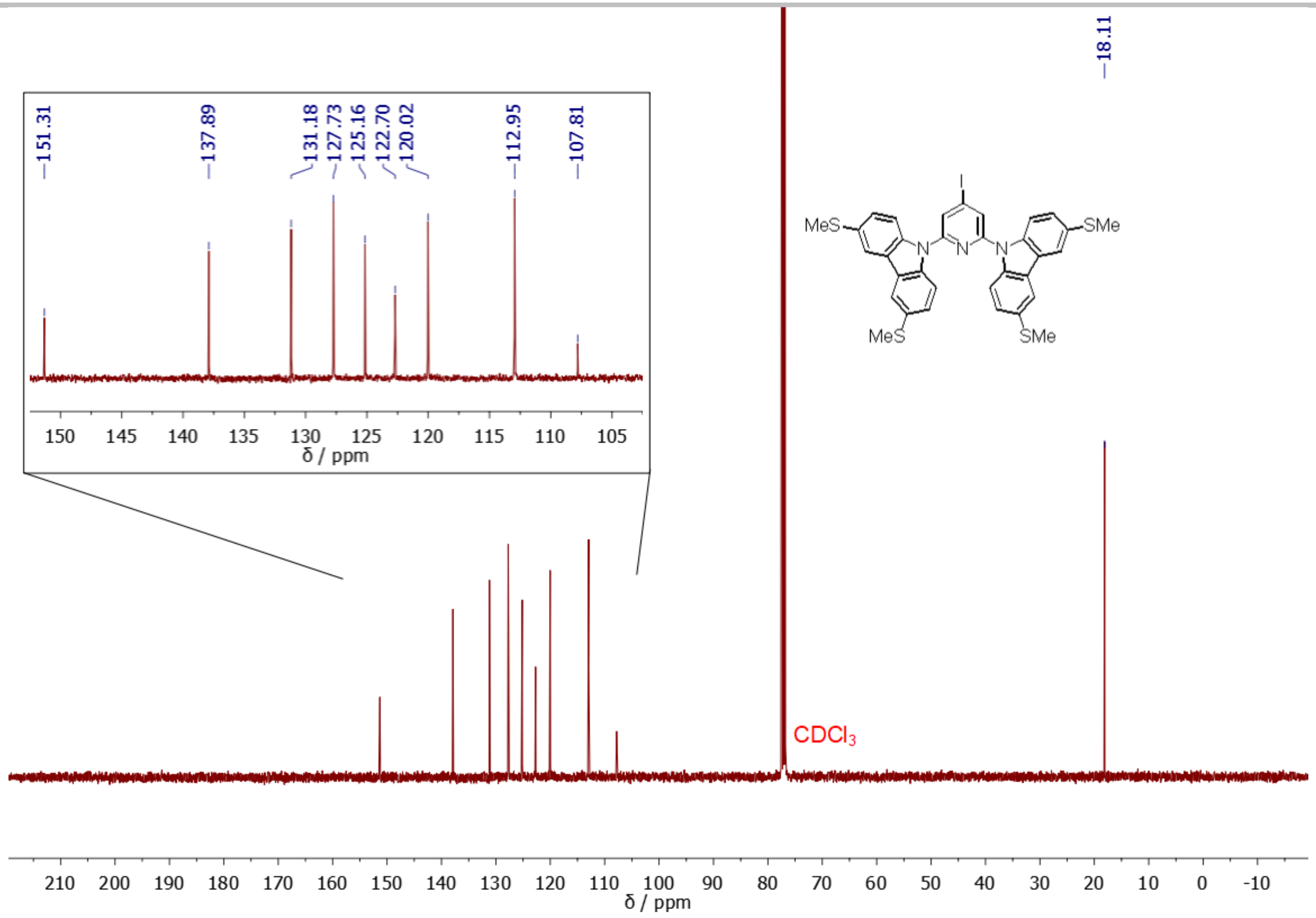

Figure S1.16. ^{13}C NMR spectrum of **4** in $CDCl_3$.

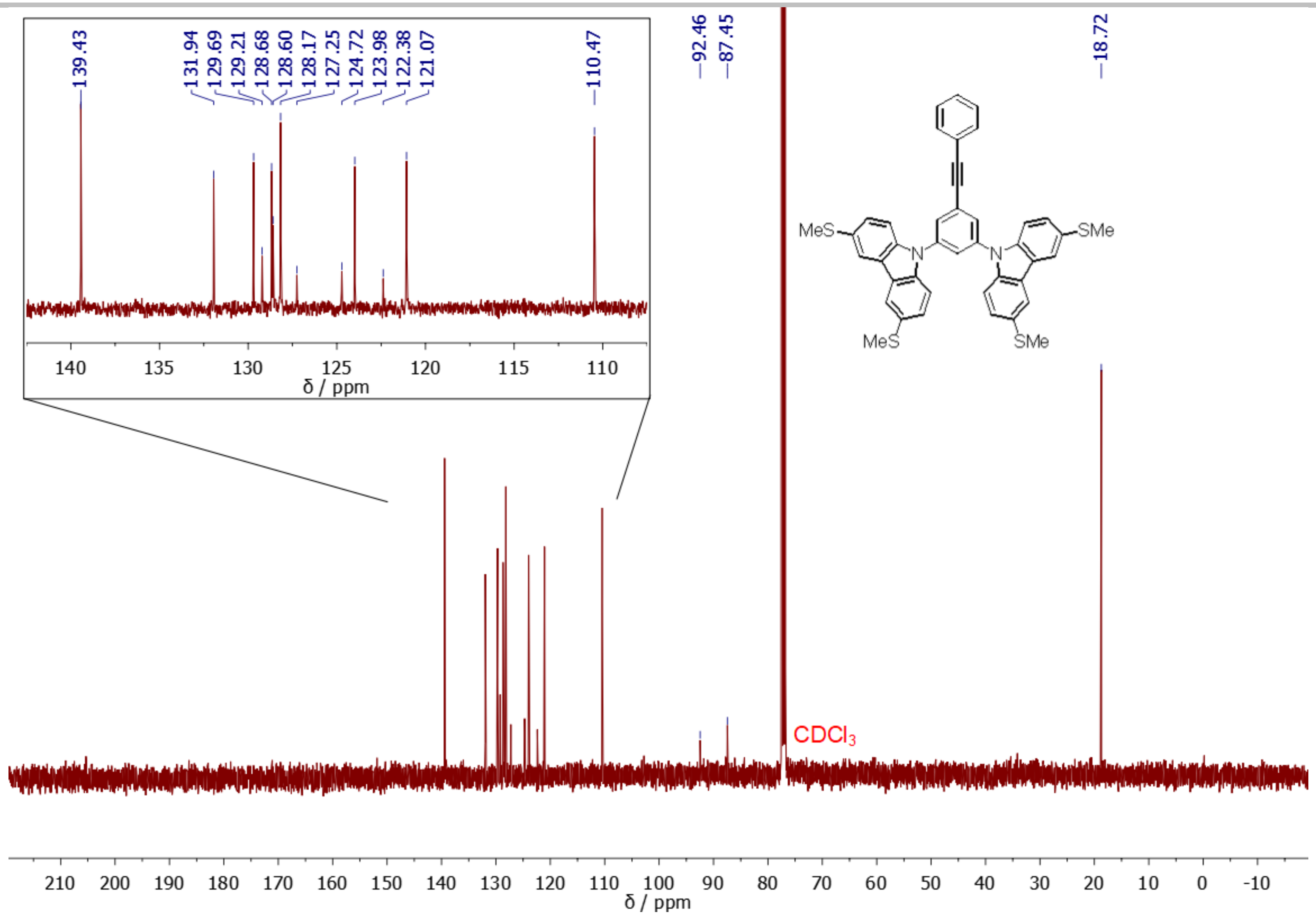

Figure S1.17. ^{13}C NMR spectrum of **BB** in $CDCl_3$.

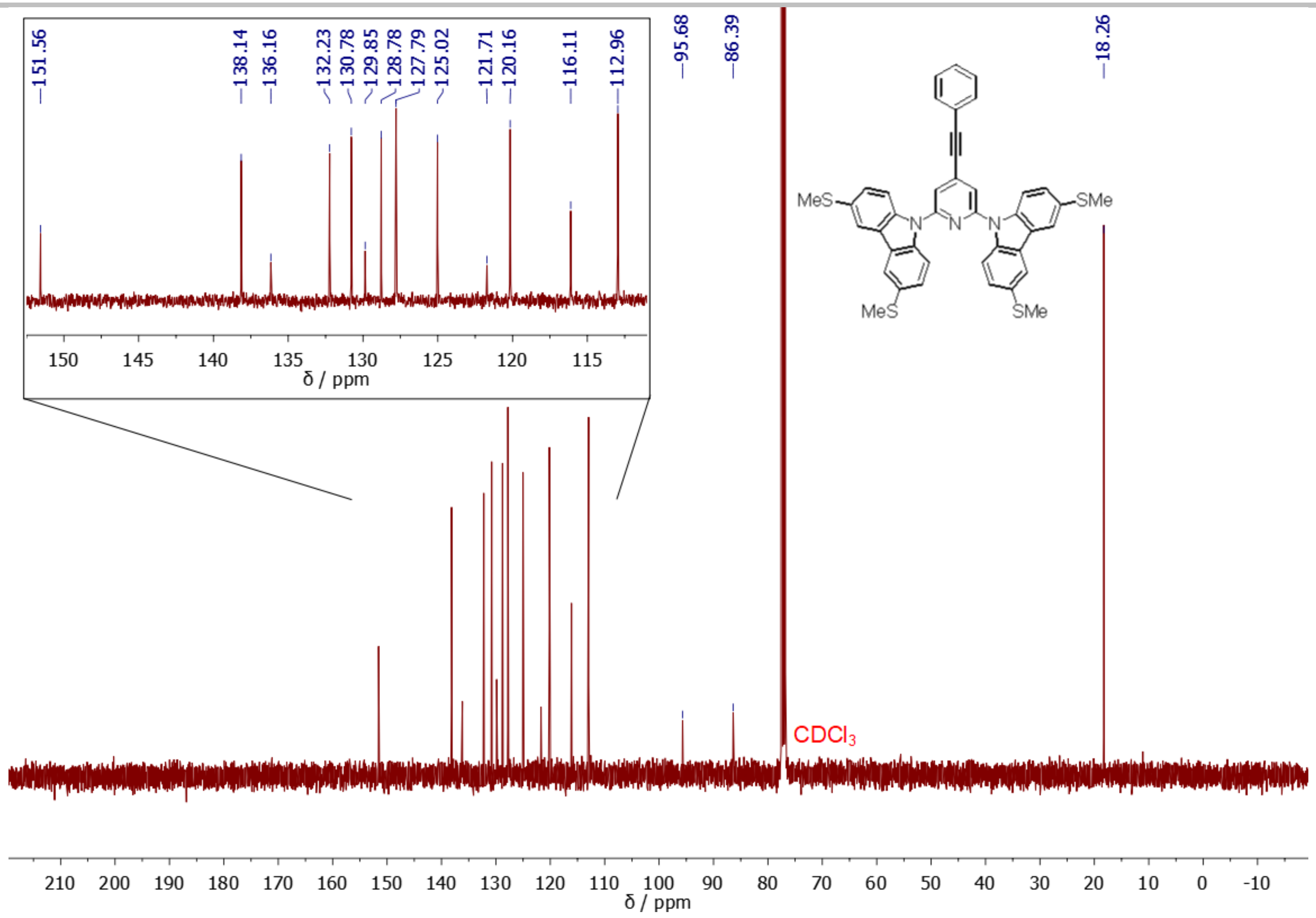

Figure S1.18. ^{13}C NMR spectrum of **PB** in $CDCl_3$.

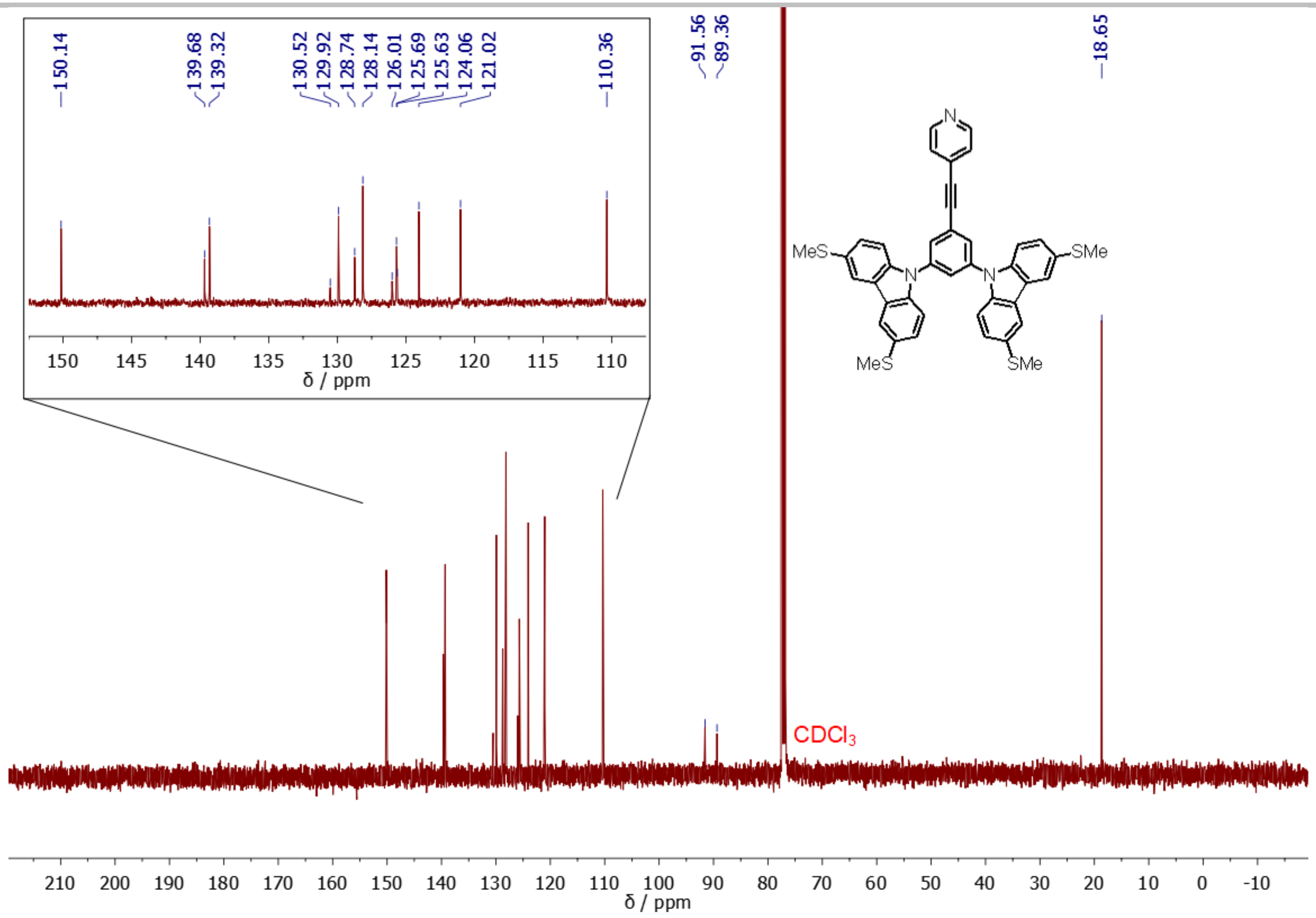

Figure S1.19. ^{13}C NMR spectrum of **B*p*P** in $CDCl_3$.

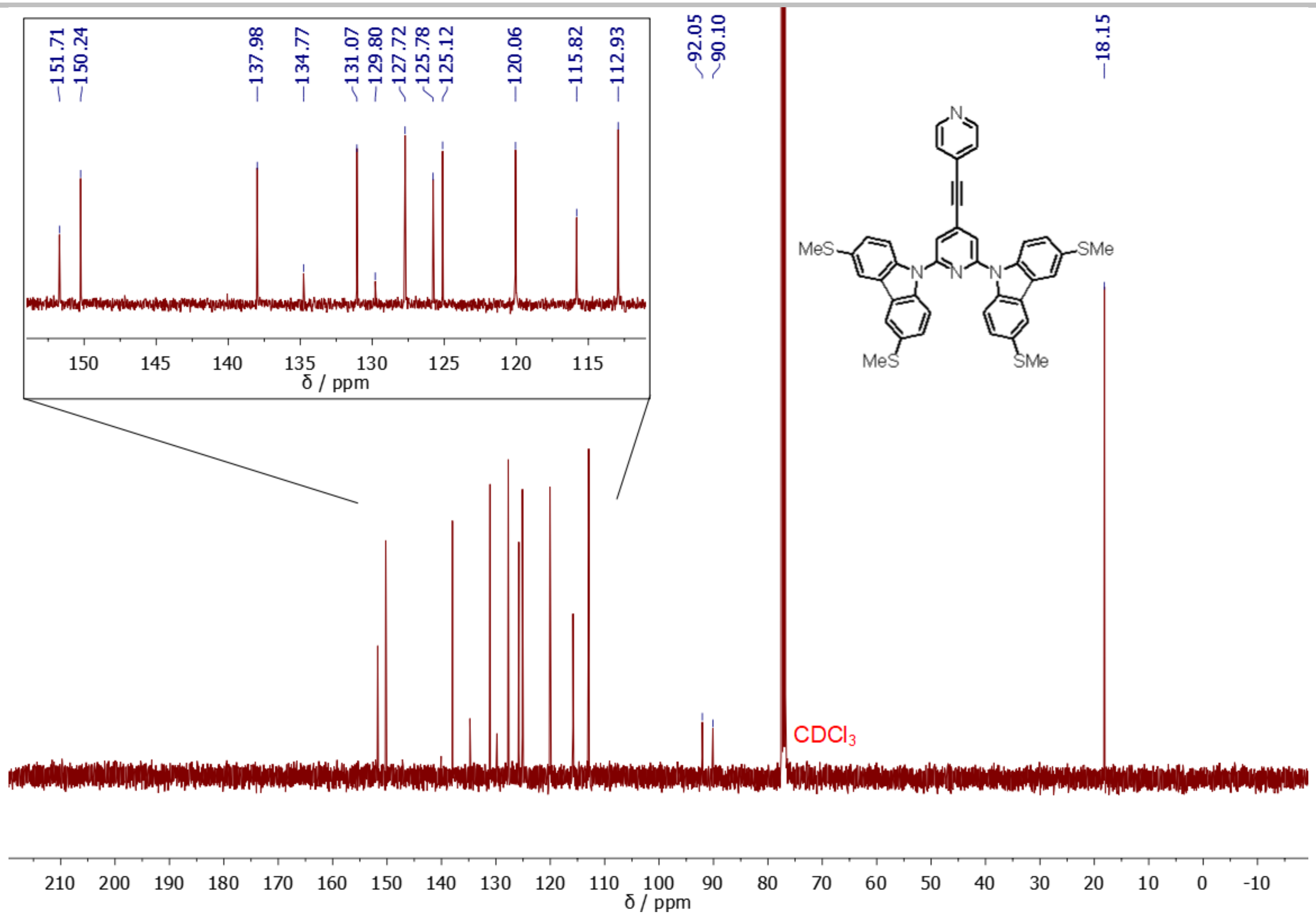

Figure S1.20. ^{13}C NMR spectrum of **P*p*P** in $CDCl_3$.

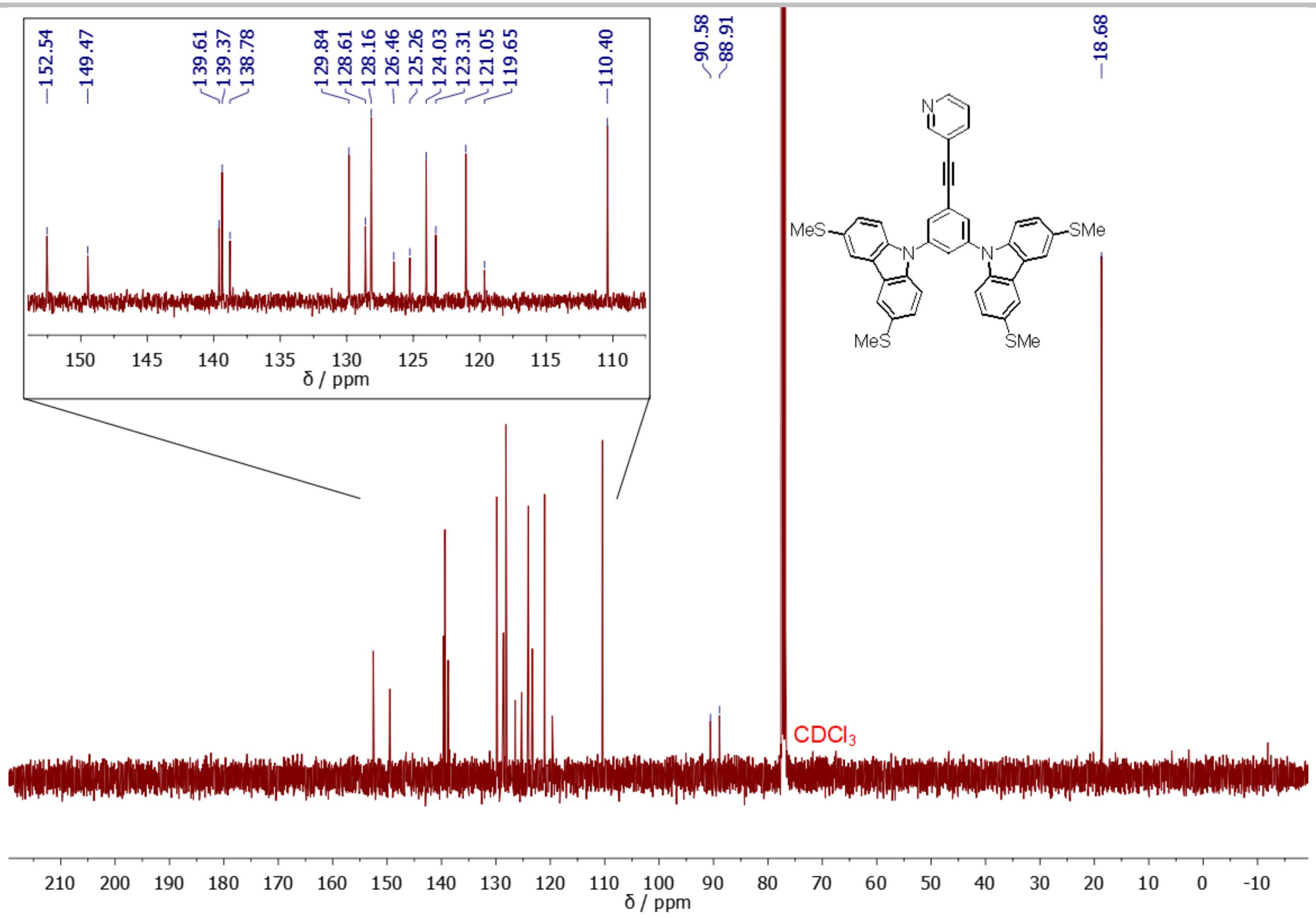

Figure S1.21. ^{13}C NMR spectrum of **B*m*P** in $CDCl_3$.

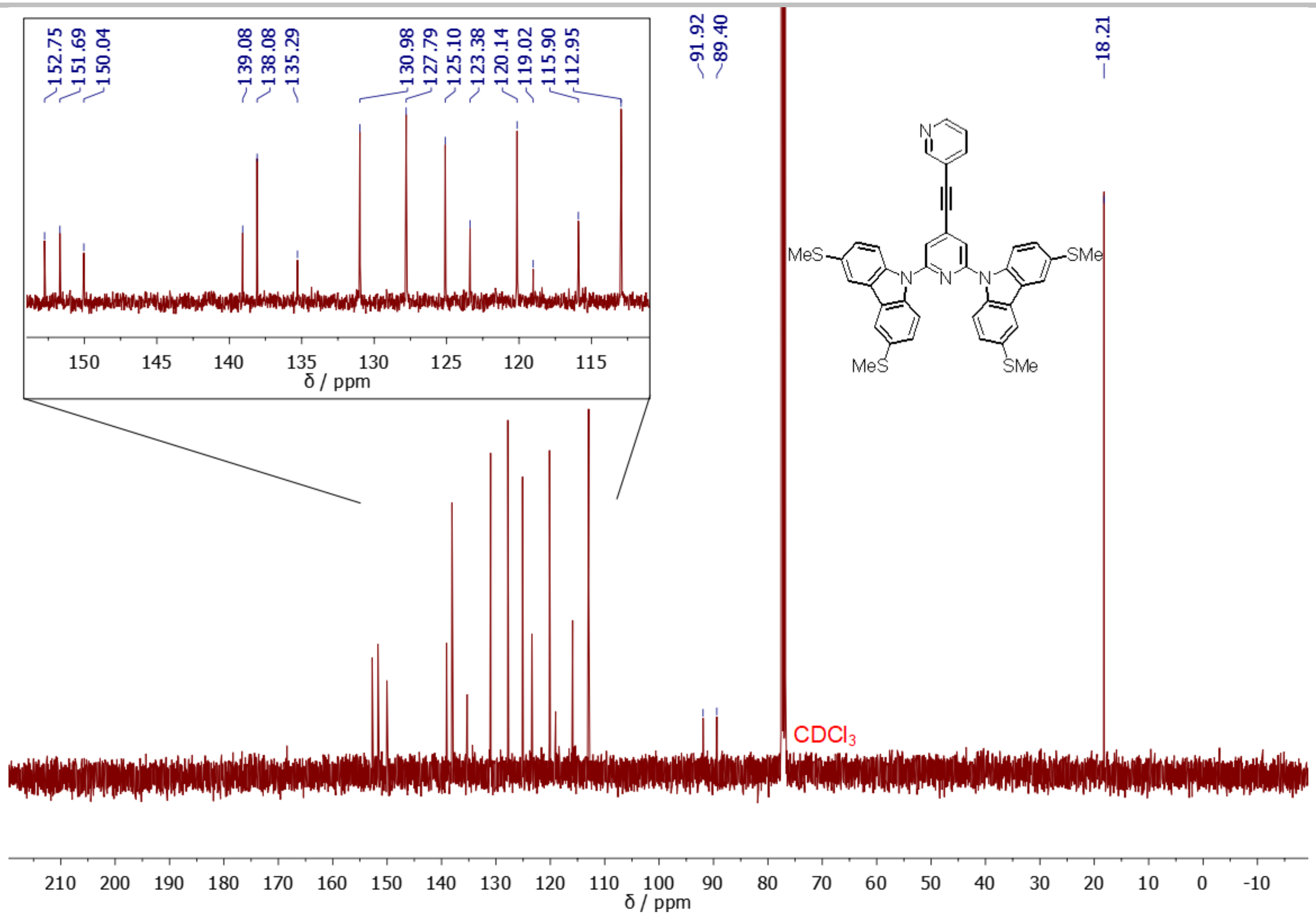

Figure S1.22. ^{13}C NMR spectrum of **P*m*P** in $CDCl_3$.

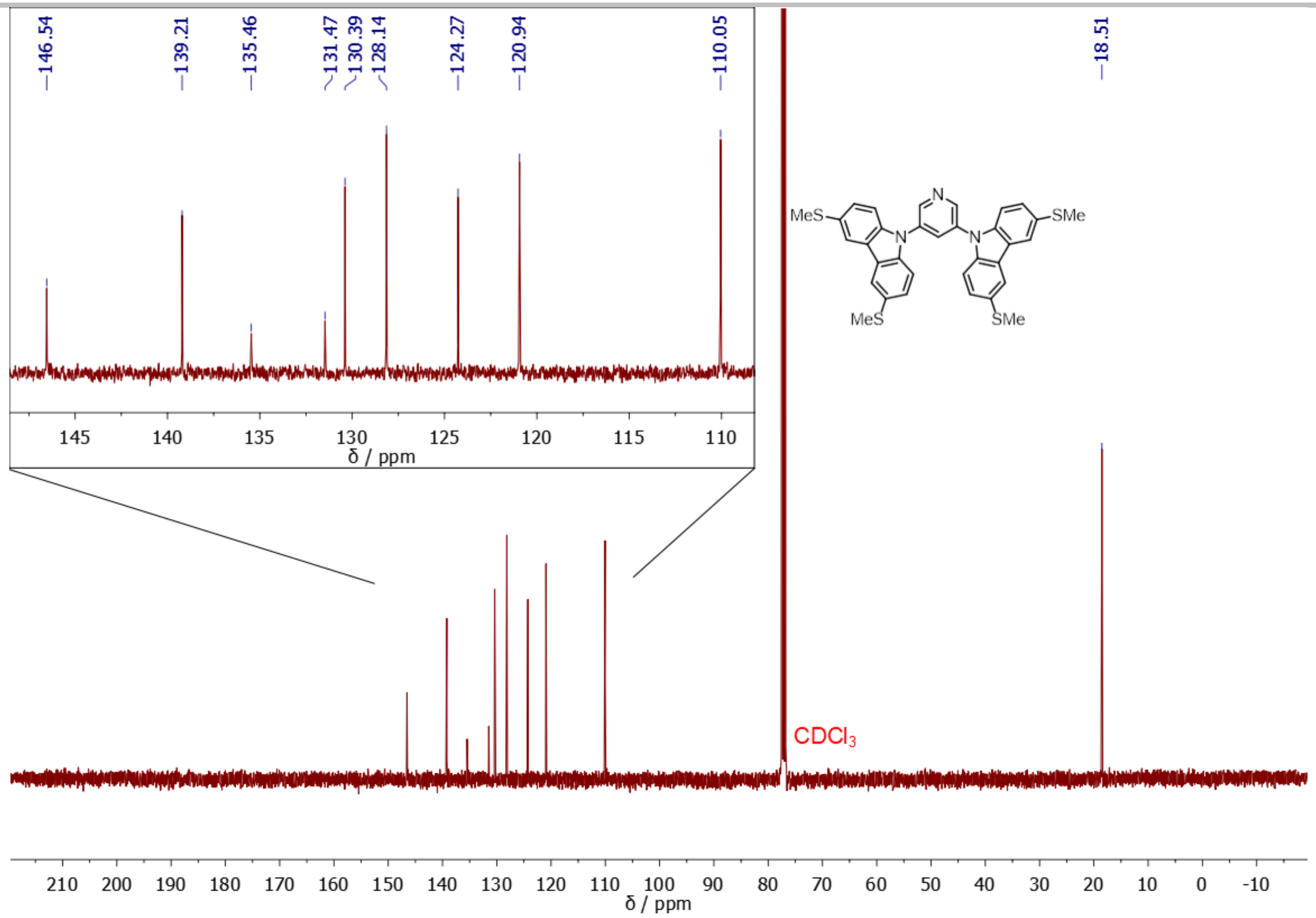

Figure S1.23. ^{13}C NMR spectrum of **B(OPE1)*pP*** in $CDCl_3$.

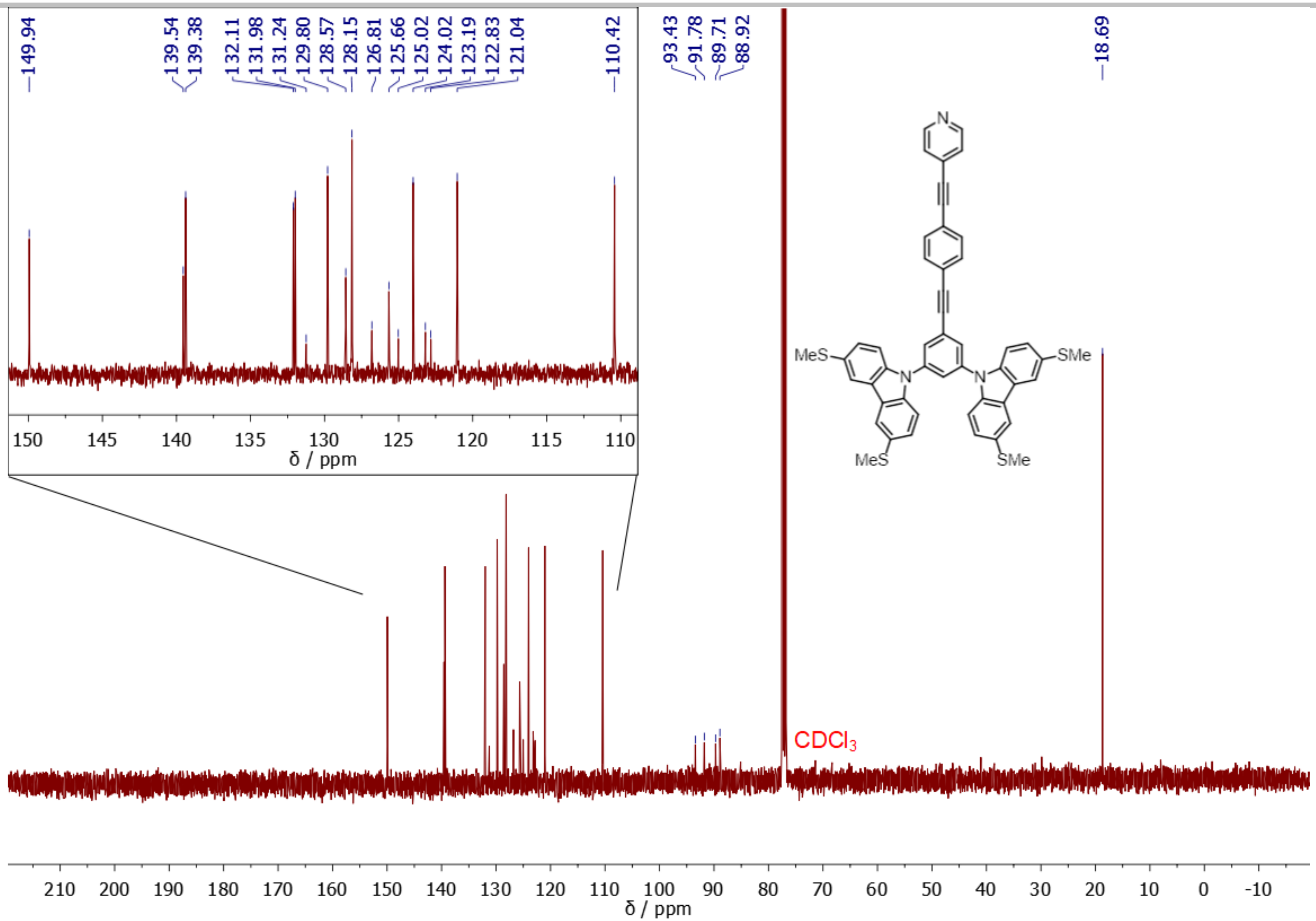

Figure S1.24. ^{13}C NMR spectrum of **B(OPE3)*p*P** in $CDCl_3$.

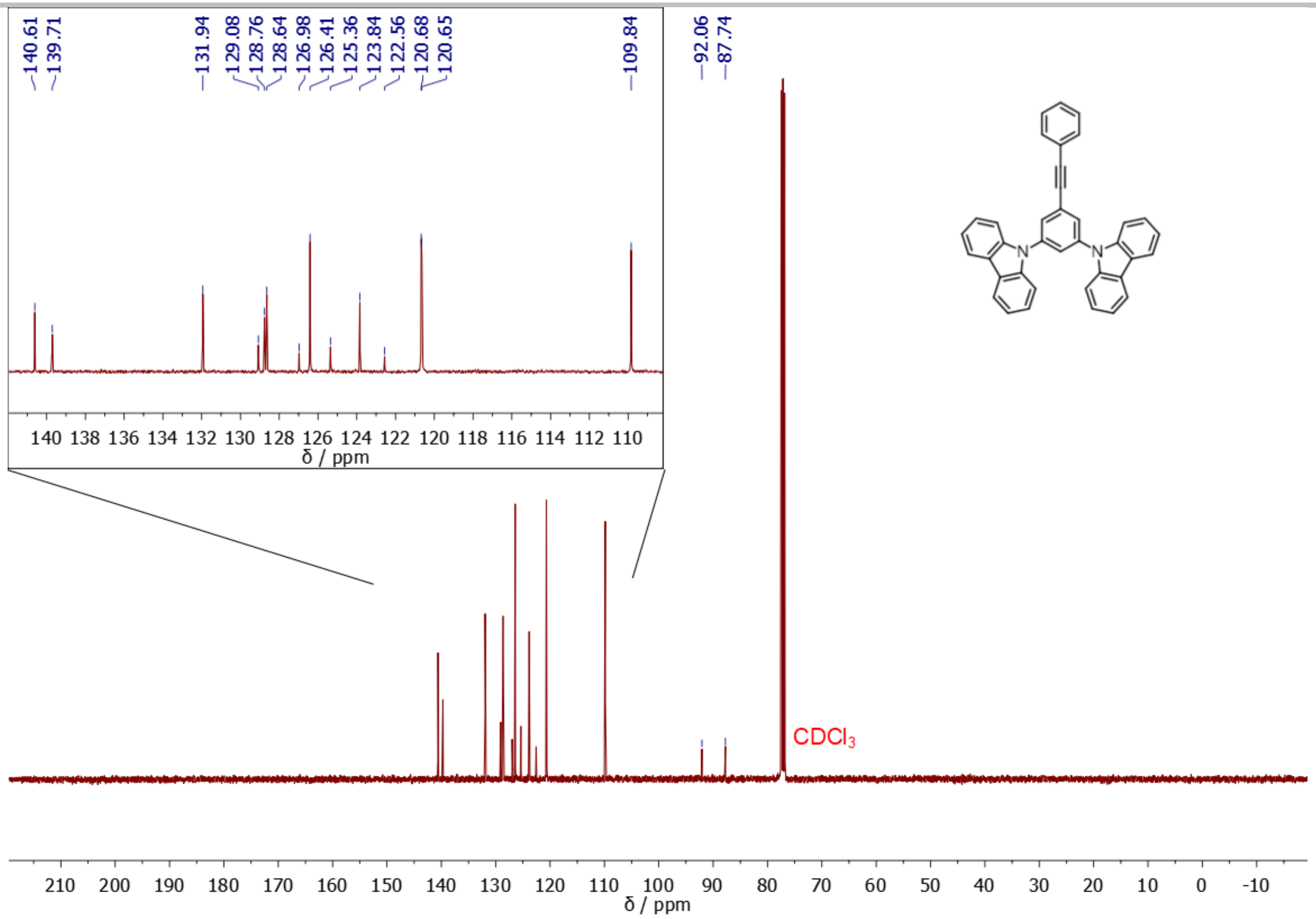

Figure S1.25. ^{13}C NMR spectrum of **1** in $CDCl_3$.

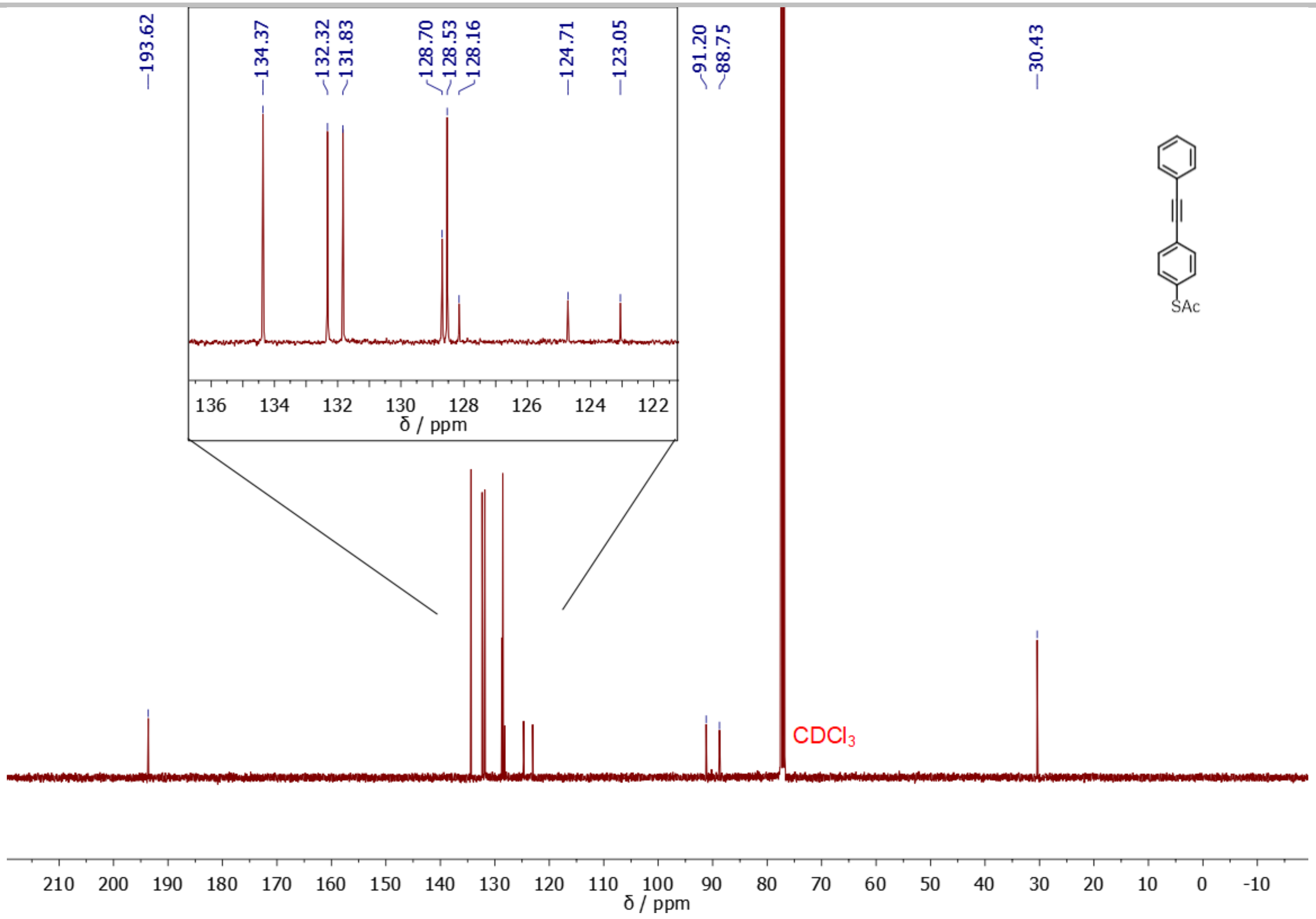

Figure S1.26. ^{13}C NMR spectrum of **SB** in $CDCl_3$.

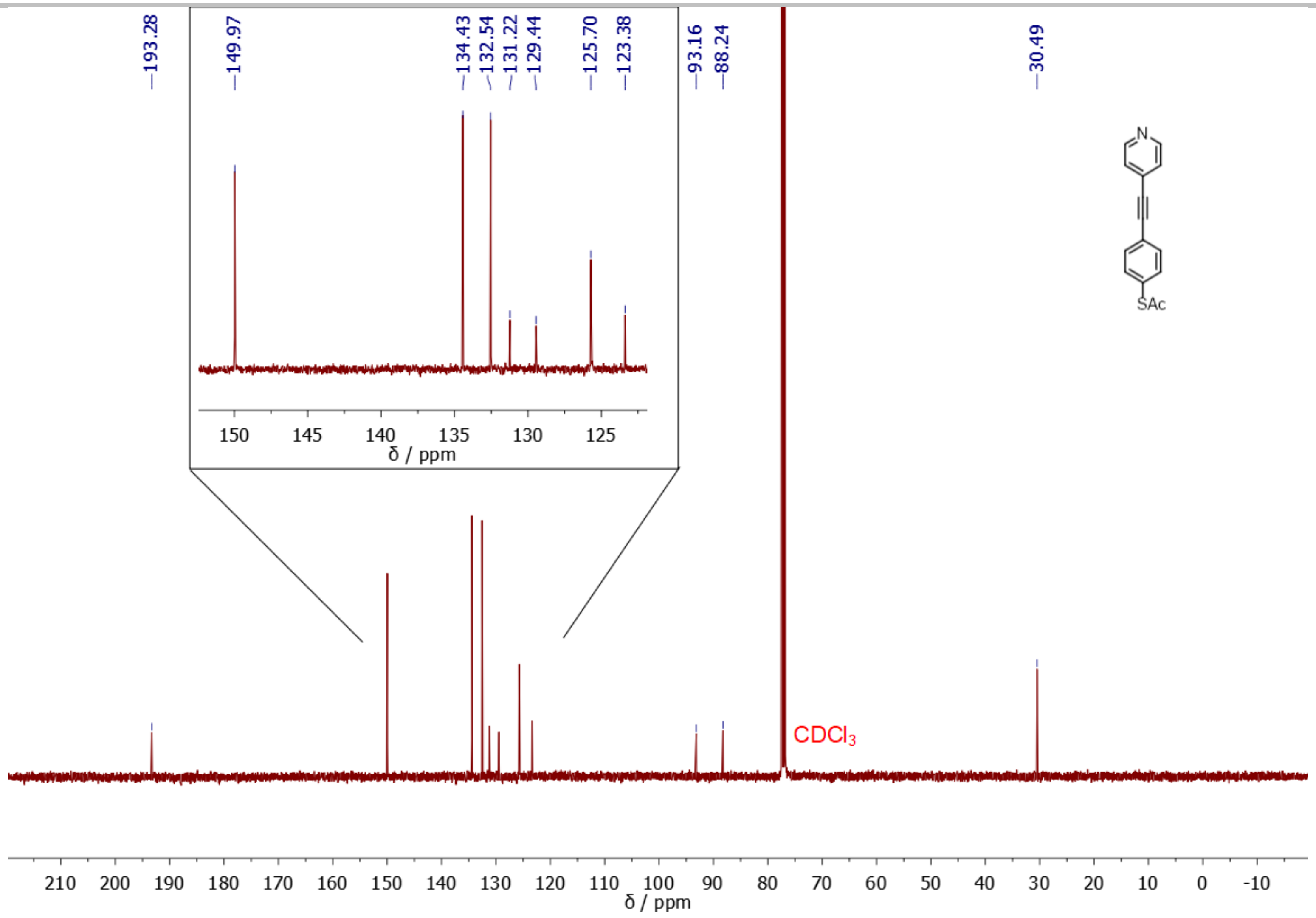

Figure S1.27. ^{13}C NMR spectrum of **S*p*P** in $CDCl_3$.

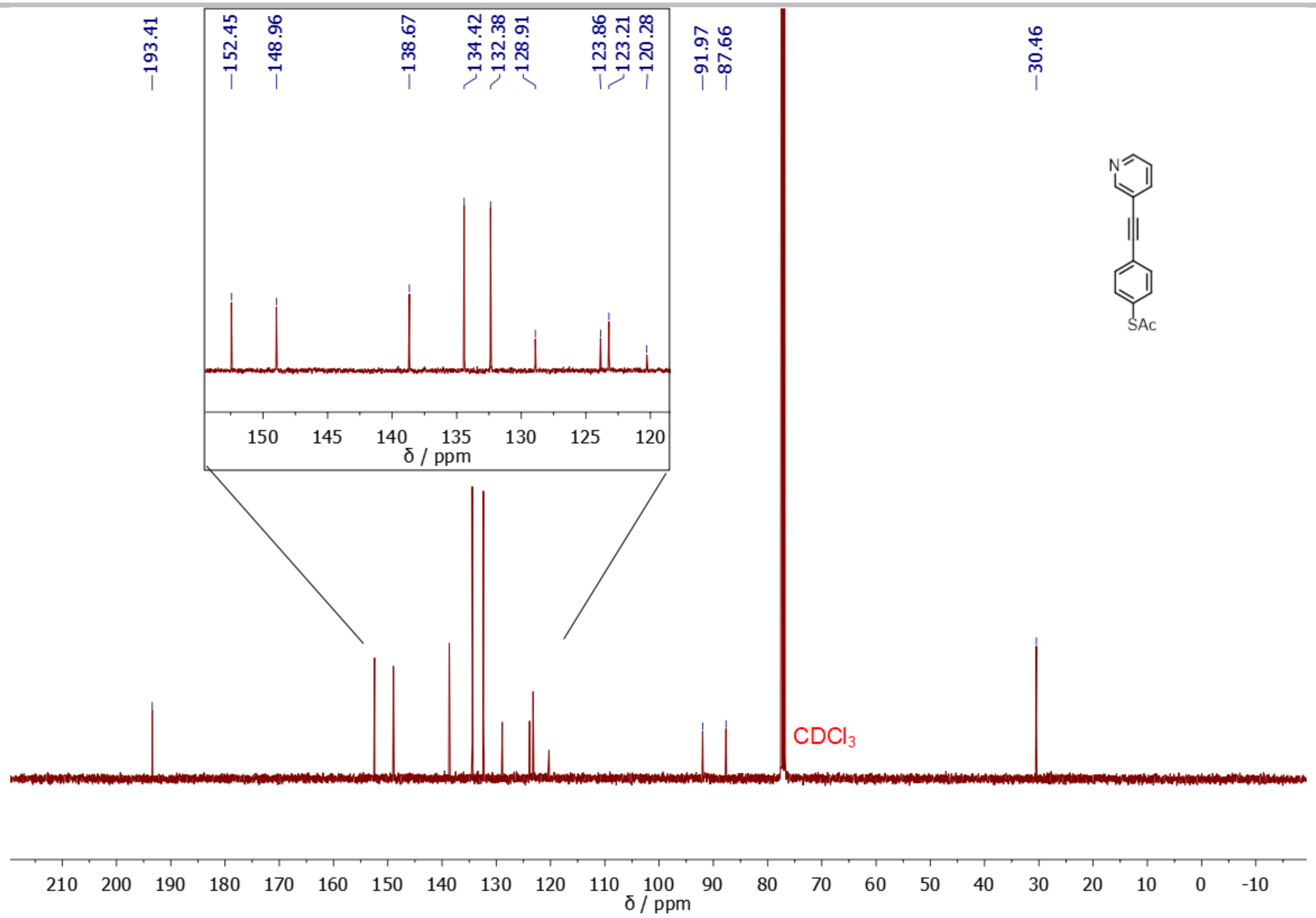

Figure S1.28. ^{13}C NMR spectrum of ***SmP*** in $CDCl_3$.

1.5 Recently Developed Anchoring Groups for Gold Surfaces

Selected examples, relevant to molecular electronics, of recently reported anchoring groups designed for enhanced binding strength on gold surfaces are summarised in Figure S1.29.

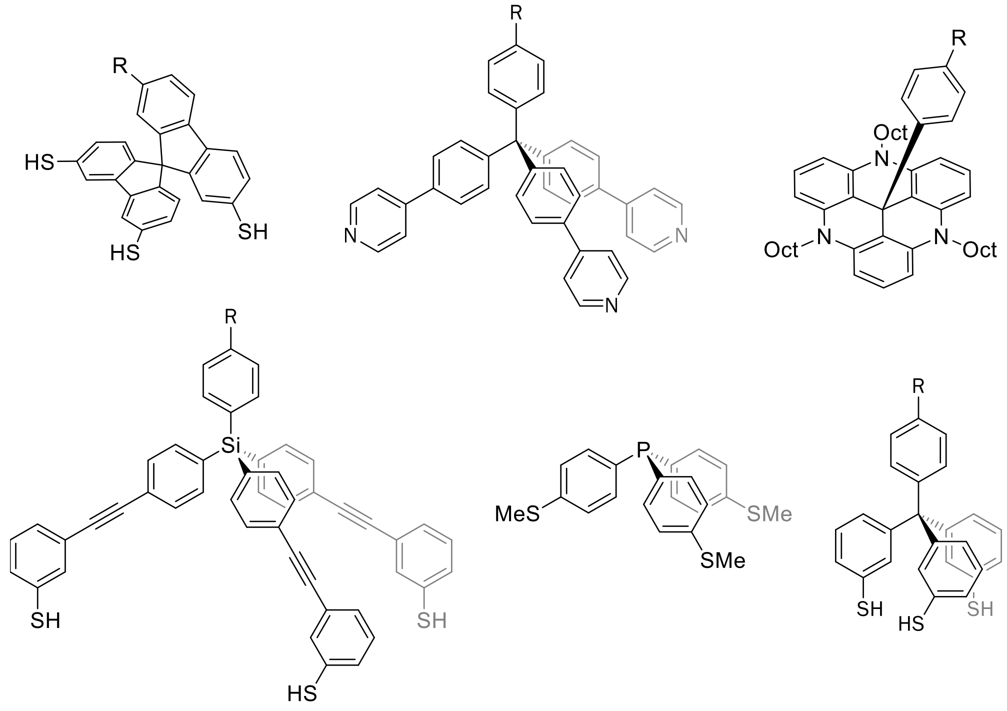

Figure S1.29. Some recently developed anchoring groups for gold surfaces, which are relevant to molecular electronics applications.[14]

*1.6 Synthesis of Model Compound **1***

To test our molecular design and proposed synthetic route, the model compound **1** was prepared in two steps as shown in Scheme S1. The route is comparable to that described in the main text for the synthesis of the OPE2 tetrapod series: the intermediate iodide **5** was prepared from carbazole and 3,5-difluoroiodobenzene *via* S_NAr then subjected to Sonogashira coupling with phenylacetylene to afford **1**.

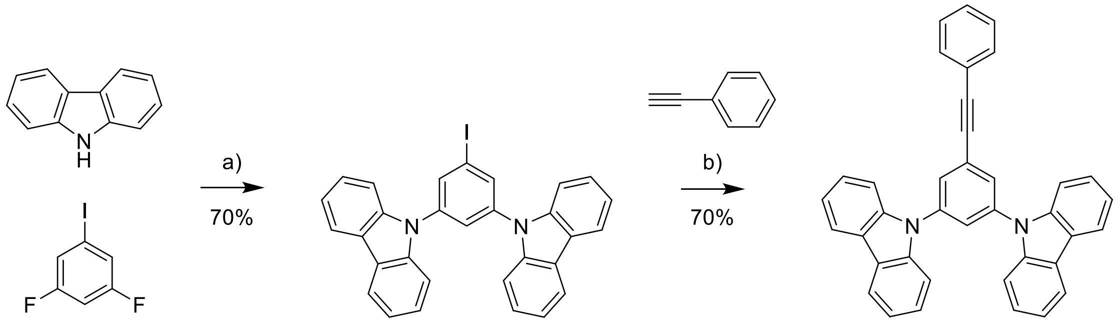

5

1

Scheme S1. Synthesis of model OPE2 **1** functionalised with two carbazole units. Reagents and conditions: a) Cs_2CO_3, DMF, 150 °C, 17 h; b) CuI, $Pd(PPh_3)_2Cl_2$, THF, DIPEA, RT, 120 min.

*1.7 Optimisation of the Synthesis of N-tert-Butyldimethylsilyl-3,6-bis(methylthio)carbazole, **6***

Our initial synthesis of **6** was based on a general literature procedure for the conversion of aryl dihalides to bis(methylthio)aryl species *via* lithiation with *n*-BuLi and treatment with dimethyl disulfide.[6] Applying these conditions to TBDMS-protected diiodocarbazole **7** afforded **6** in only 27% yield, with extensive by-product formation (see Scheme S2 and Table S1). Notably, significant quantities of *n*-

butylated carbazoles were isolated. The yield of **6** was improved slightly by increasing the excess of dimethyl disulfide used, but this coincided with a reduction in hydrogen-substitution rather than in butylation. Switching to ^{t}BuLi prevented butylation of carbazole but, when using 4 eq., resulted in a lower yield of **6**, with considerable hydrogen-substitution and partial *N*-deprotection. Using only 2 eq. of ^{t}BuLi gave an improved yield of 38%, with hydrogen substitution still evident, as well as evidence of incomplete lithiation. By slightly increasing the stoichiometry of ^{t}BuLi to 2.5 eq., around 80% yield of **6** was reproducibly achieved, with the only significant by-product being that with a single thiomethyl substituent, the other reactive site being hydrogen-substituted (**6a**). The TBDMS protecting group of **6** was readily cleaved using TBAF to afford the key building block 3,6-bis(methylthio)carbazole, **2**.

Table S1. Summary of syntheses of *N-tert*-butyldimethylsilyl-3,6-bis(methylthio)carbazole, **6**. Structures are shown in Scheme S2.

Conditions[a]	% Yield of **6**[b]	% Yield of **6a**[b]	% Yield of **6b**[b]	% Yield of **6c**[b]	% Yield of **2**[b]	% Yield of **6d**[b]	% Yield of **6e** [b]	Other by-products
2.2 eq. ^{n}BuLi, 2.2 eq. MeSSMe, 0.53 g scale	27	17	15	*[c]	-	-	-	Mix of **6c**; **6f** and **6g** (not separated, 32-44%)[d]
2.1 eq. ^{n}BuLi, 4.0 eq. MeSSMe, 2.2 g scale	32	8	34	3	-	-	-	Mix of **6f** and **6g** (not separated, 25-29%)[d]
4.0 eq. ^{t}BuLi, 4.0 eq. MeSSMe, 2.0 g scale	14	21	-	17	21	-	-	ca. 100 mg unidentified white solid
2.0 eq. ^{t}BuLi, 4.0 eq. MeSSMe, 1.3 g scale	38	21	-	ca. 24	-	9	ca. 8	-
2.5 eq. ^{t}BuLi, 4.0 eq. MeSSMe, 1.9 g scale	83	17	-	-	-	-	-	-
2.5 eq. ^{t}BuLi, 4.0 eq. MeSSMe, 3.0 g scale	79	17	-	trace	-	-	-	-

[a] Timings, temperatures, etc. are as described in the experimental section above; [b] isolated yields after column chromatography, by-product yields may be overestimates as attempts were not made to ensure these materials were analytically pure; [c] see other by-products; [d] Components of mixture identified using ^{1}H NMR spectroscopy, with yield range based on highest and lowest molecular weights of by-products.

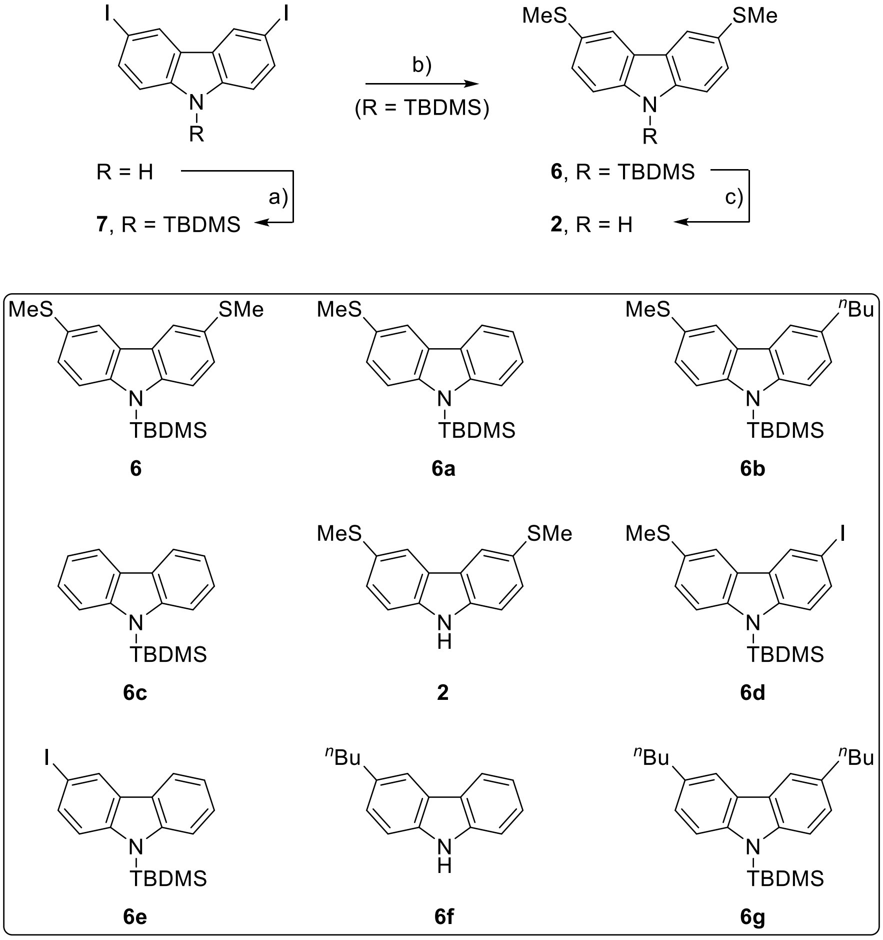

Scheme S2. Synthesis of *N-tert*-butyldimethylsilyl-3,6-bis(methylthio)carbazole, **6**. In box: products and by-products obtained when optimising the synthesis of **6.** Reagents and conditions: a) i. NaH, THF, RT, 30 min; ii, TBDMSCl, RT, 17 h; b) i. ^{t}BuLi, THF, −78 °C, 10 min; ii. MeSSMe, −78 °C – RT, 90 min; c) TBAF, THF, RT, 15 min.

1.8 Synthesis of Thioacetate Functionalised Series

For comparison with our new anchoring motif, an analogous series of molecular wires were prepared with simple protected thiol anchors, which are widely used in molecular conductance studies. The synthesis of this series is summarised in Scheme S3. 1-(Acetylthio)-4-iodobenzene **8** was prepared from 1,4-diiodobenzene using a method based on a thioacetylation procedure reported by Soria-Castro and Peñéñory.[15] In a statistical reaction using an excess of 1,4-diiodobenzene, which can be easily re-isolated from the reaction, **8**, a key building block in the field of molecular electronics, can be conveniently prepared from readily available reagents in a single step. Further optimisation may be possible if a larger excess of diiodobenzene is used. We favoured this approach over existing methods for the preparation of **8** which require multiple steps[16] or the use of more costly or more hazardous reagents.[11, 17] The synthesis of the three targeted wires was completed by Sonogashira couplings with the relevant alkyne.

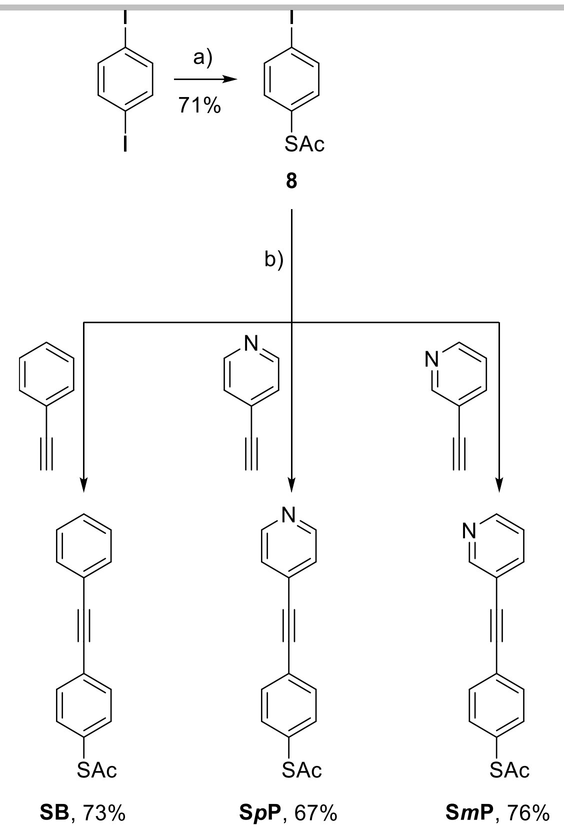

Scheme S3. Synthesis of thioacetate-functionalised OPE2 wires. Reagents and conditions: a) KSAc, CuI, 1,10-phenanthroline, PhMe, reflux, 2.5 d; b) CuI, $Pd(PPh_3)_2Cl_2$, THF, DIPEA, RT, 18 h.

2. Materials Characterisation and Properties

2.1 X-Ray Crystallography

X-ray single crystal data were collected on a Bruker 3-circle D8 Venture diffractometer with a Photon100 CMOS detector, using Mo-*Kα* radiation (λ = 0.71073 Å) from a IμS-microsource with focusing mirrors. The crystals were cooled with a Cryostream (Oxford Cryosystems) open-flow N_2 cryostat. Crystals of **1** undergo a phase transition on cooling below 170 K, whereupon reflections become diffuse. The structure of **1** was solved by direct methods using the SHELXS 2013/1 program.[18] The structure of **B*m*P** was solved by dual-space intrinsic phasing using the SHELXT 2018/2 program.[19] Both structures were refined by full-matrix least squares using SHELXL software[20] on OLEX2 platform.[21] Selected crystal data and experimental details are listed in Table S2. Full crystallographic information (including structure factors) in CIF format has been deposited with Cambridge Crystallographic Data Centre.

In molecule **1** (Figure S2.01) the two planar carbazole moieties are inclined with respect to the central arene ring in a propeller-like fashion, with interplanar angles of 60.2° and 56.4°, while the terminal phenyl ring is inclined by 9.1°. Unlike **1**, **B*m*P** (Figure S2.02) shows extensive disorder in the crystal, viz. the pyridyl ring is disordered by a 180° rotation (i.e. the N atom is distributed between two *meta*-positions), one entire bis(methylthio)carbazole moiety is disordered between two positions differing by a ca. 11.5° libration within its own plane (further complicated by opposite orientations of one SMe groups), while the other bis(methylthio)carbazole moiety has one disordered SMe group. In each case, the disorder is in a 1:1 ratio. The disordered carbazole plane is inclined to the central arene ring by 54.6° or 54.4°, the ordered carbazole by 38.6°, and the terminal pyridyl ring by 9.6°.

Table S2. Crystal data and experimental details

Compound	**1**	**B*m*P**
CCDC	1950944	1950945
Formula	$C_{38}H_{24}N_2$	$C_{41}H_{31}N_3S_4$
$D_{calc.}$/ g cm^{-3}	1.264	1.378
μ/mm^{-1}	0.074	0.320
Formula Weight	508.59	693.93
T/K	190	120
Crystal System	monoclinic	monoclinic
Flack Parameter	.	0.07(5)
Space Group	$P2_1/c$ (no.14)	*Ia* (no. 9)
a/Å	18.2601(12)	8.1891(12)
b/Å	8.2084(6)	22.548(3)
c/Å	19.6494(13)	18.328(3)
β/°	114.834(3)	98.643(5)
V/Å^3	2672.8(3)	3345.8(8)
Z	4	4
$2\theta_{max}$/°	50	50
Reflections total	36618	25881
unique	4721	5890
with $I > 2\sigma(I)$	2966	5406
R_{int}	0.098	0.072
Parameters	362	468
Restraints	0	311
Δρ min,max/eÅ^{-3}	0.21, -0.20	0.38, -0.47
Goodness of fit	1.114	1.142
wR_2 (all data)	0.161	0.151
wR_2 [$I > 2\sigma(I)$]	0.144	0.148
R_1 (all data)	0.119	0.069
R_1 [$I > 2\sigma(I)$]	0.068	0.062

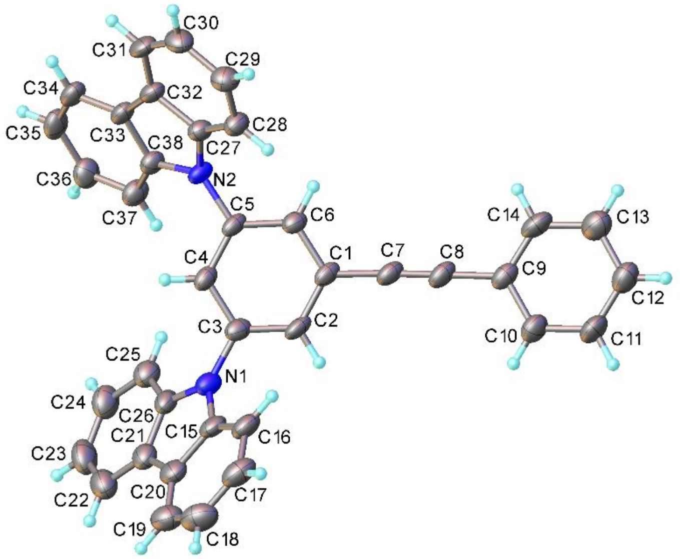

Figure S2.01. X-ray molecular structure of **1**. Thermal ellipsoids are drawn at the 50% probability level.

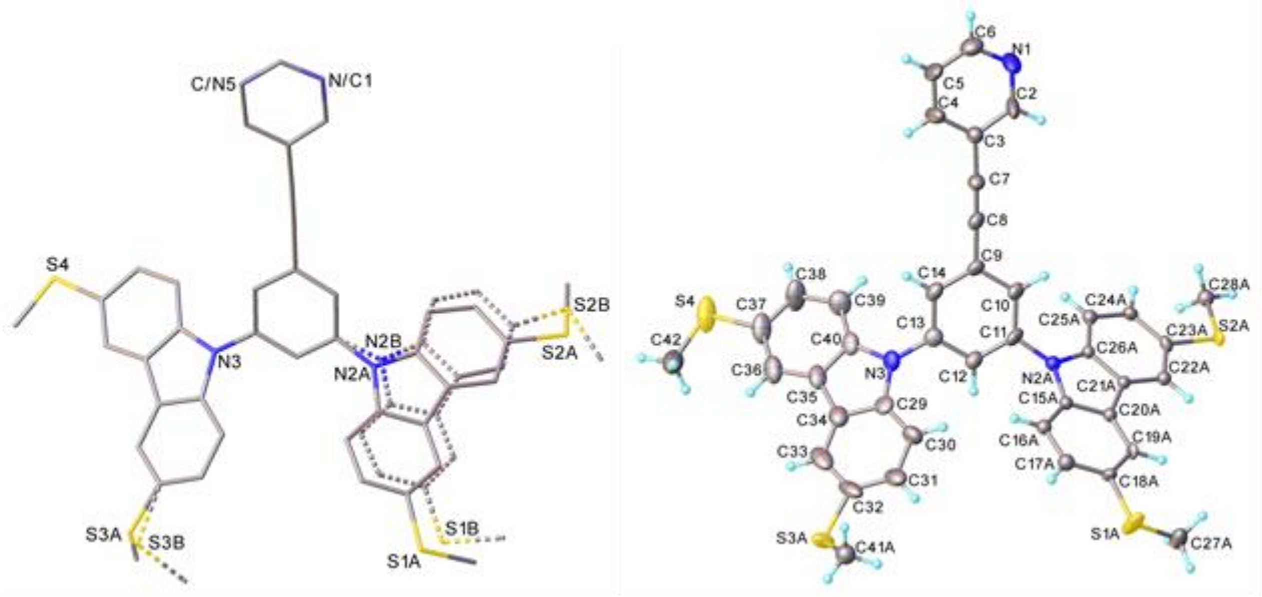

Figure S2.02. Left: The disorder of the **B*m*P** molecule in crystal (H atoms are omitted). Right: One of the possible conformations of **B*m*P** (thermal ellipsoids are drawn at the 50% probability level).

2.2 UV-Visible Absorption Spectroscopy

UV-visible absorption spectroscopy was conducted using a Thermo Evolution 220 UV-visible spectrometer and the supplied Thermo Insight Software. Figure S2.03 shows normalised UV-vis spectra for the six tetrapodal OPE2 wires and Figure S2.04 shows corresponding spectra for the series of tetrapodal wires with increasing length (from shortest to longest: **B(OPE1)*p*P**, **B*p*P** and **B(OPE3)*p*P**). Spectra were recorded for 10 µM solutions of each material in DCM and normalised relative to the maximum absorbance (254 nm in all cases) for ease of comparison. It can be seen that the spectra of the three benzene-based (X = CH) OPE2 compounds are very similar, suggesting that changes to the head group have little effect on the absorbance of the materials. This is also the case for the pyridine-based (X = N) OPE2 materials, however, these species show a shoulder peak extending out ca. 430 nm compared to only ca. 385 nm for the benzene-based series. This is ascribed to a combination of: i) weak charge transfer (CT) interactions between the electron-rich carbazole units and the electron-withdrawing central pyridine ring, and ii) a hydrogen bonding interaction between the pyridine nitrogen atom and the hydrogen atoms in the carbazole 1- and 8-positions. The latter reduces the twist angle between the carbazole and OPE π-systems, increasing conjugation and red-shifting absorbance, and facilitating any CT interaction. The proposed hydrogen bonding interaction is supported by the dramatically different chemical shift (ca. 0.5 ppm) of the signals associated with

protons in the carbazole 1- and 8-positions in the ^{1}H NMR spectra of otherwise equivalent benzene-based and pyridine-based species. The spectra of the iodide-precursors in Figures S1.01 and S1.02 (above) allow the easiest comparison of this effect: the 4H dd at ca. 7.42 ppm in Figure S1.01 shifts to ca. 7.94 ppm in Figure S1.02 when the base is changed from benzene to pyridine. The reduced twist angle in the case of pyridine species is also observed in DFT geometry relaxations (see Figure 1 in the main text). The absence of any significant spectral variation as the head group is changed leads us to conclude that there is no significant charge transfer between the electron-rich carbazole units and the head group. As would be expected based on increased conjugation, lengthening the conductive backbone results in increased absorbance at higher wavelengths. The larger shoulder of **B(OPE1)*p*P** at ca. 350 nm relative to the longer **B*p*P** molecule presumably relates to the central ring of the shorter species being a pyridine rather than a benzene. This could permit some weak CT interactions which are not observed in the longer analogues with a benzene ring in this position.

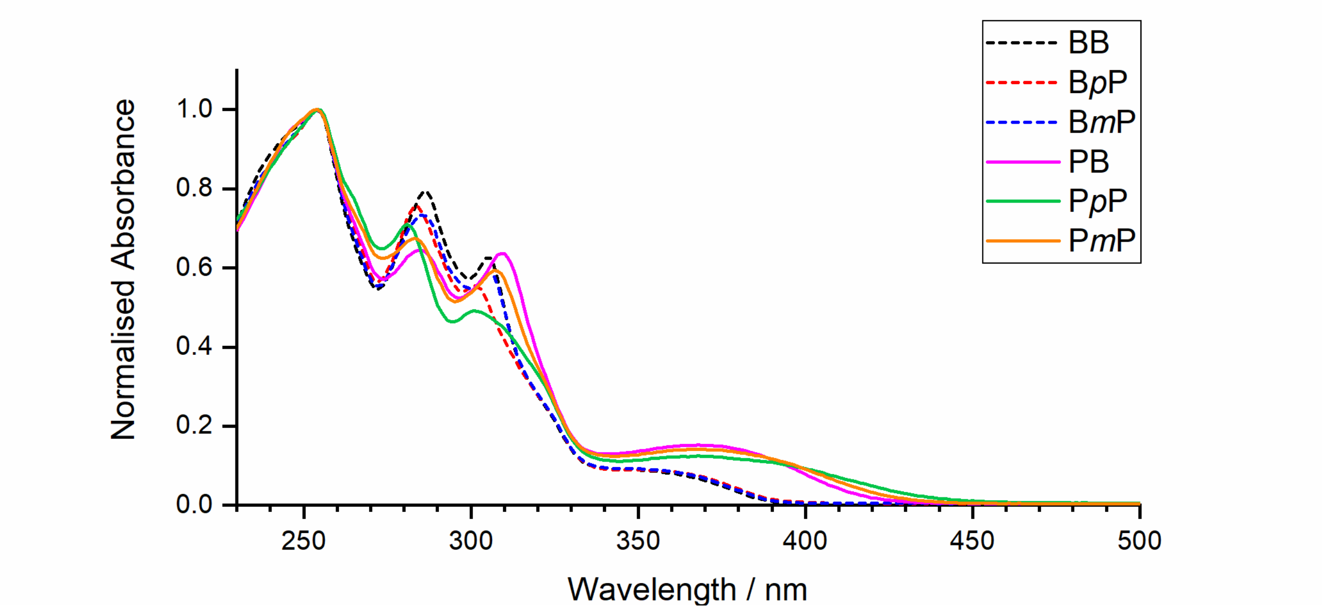

Figure S2.03. Normalised UV-visible spectra of 10 µM solutions of tetrapodal OPE2 wires in DCM. In all cases, no absorbance was detected between 500 and 1100 nm (not shown). Dashed lines indicate benzene-based (X = CH) molecules, and solid lines pyridine-based molecules (X = N).

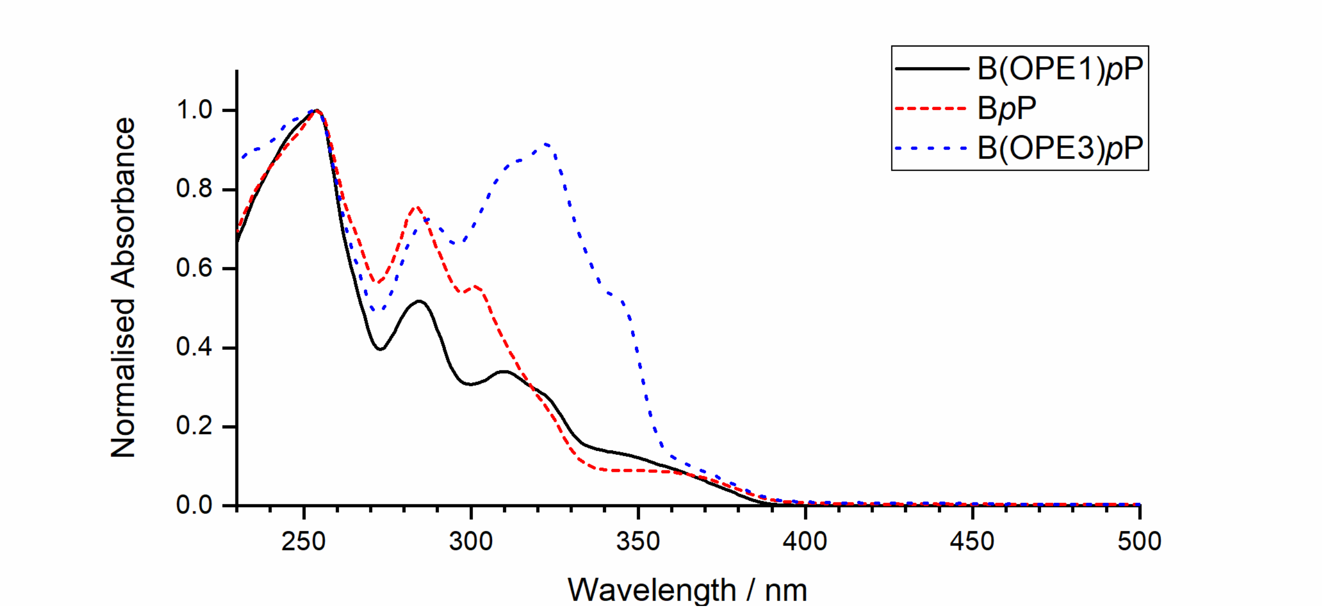

Figure S2.04. Normalised UV-visible spectra of 10 µM solutions of tetrapodal wires of different lengths in DCM. In all cases no absorbance was detected between 500 and 1100 nm (not shown).

2.3 SAM Formation and Imaging

Au surface preparation: Ultra-flat gold surfaces were prepared by modifying the template stripping method of Whitesides and Pinkhassik.[22] A 100 nm-thick layer of gold was deposited on a 1 cm × 1 cm Si substrate by thermal evaporation. A Si wafer (5 mm × 5 mm) was washed in an ultrasonic bath sequentially with acetone, methanol and isopropanol, then cleaned with oxygen plasma for 5 min. The cleaned wafer was glued onto the gold surface using EPO-TEK 353ND epoxy adhesive to form a Si/glue/Au/Si sandwich structure. After curing the glue at 150 °C for 40 min, the ultra-flat template-stripped gold was obtained by mechanical cleavage. AFM scans were performed on all gold substrates before use, and only those with roughness < 150 pm were used for SAM growth.

SAM preparation: 1 mM solutions of the molecules to be tested were prepared in a 1:5 ethanol:toluene mixture then degassed by bubbling with nitrogen for 5 min to eliminate oxygen. The template stripped gold was immersed in the solution for 3 h then rinsed several times with toluene and ethanol to wash off any physisorbed molecules. After washing, the sample was incubated in a vacuum oven (40 °C, 10^{-3} Pa) overnight to remove residual solvent.

Imaging and surface characterisation: All AFM work was done using a Multi-Mode 8 (Bruker) instrument. Image scans used peak force mode (peak force setpoint at 500 pN) with a Multi-75 probe (Budget Sensors). Representative images are shown in Figure S2.05. Nano-scratching was done by scanning the image at 40 nN in contact mode to scratch molecules from the surface (we experimentally proved this force is enough to scratch organic surfaces but not enough to do any damage to the gold),[23] followed by using peak force mode to obtain the height difference. Representative images and cross-sectional height profiles are shown in Figure S2.06.

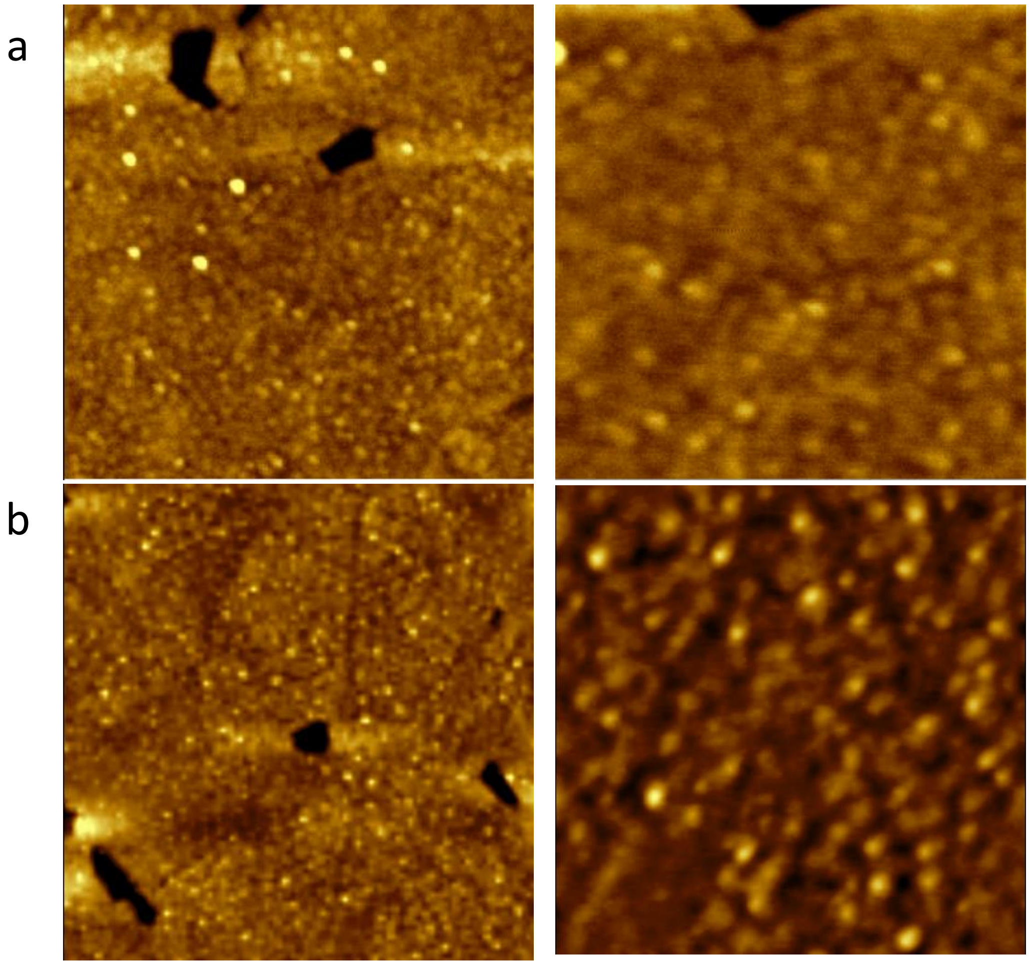

Figure S2.05. AFM topography of SAMs on template-stripped gold of a) **B*p*P**; b) **B(OPE3)*p*P**. Scan areas: 1 µm x 1 µm (left) and 400 nm x 400 nm (right).

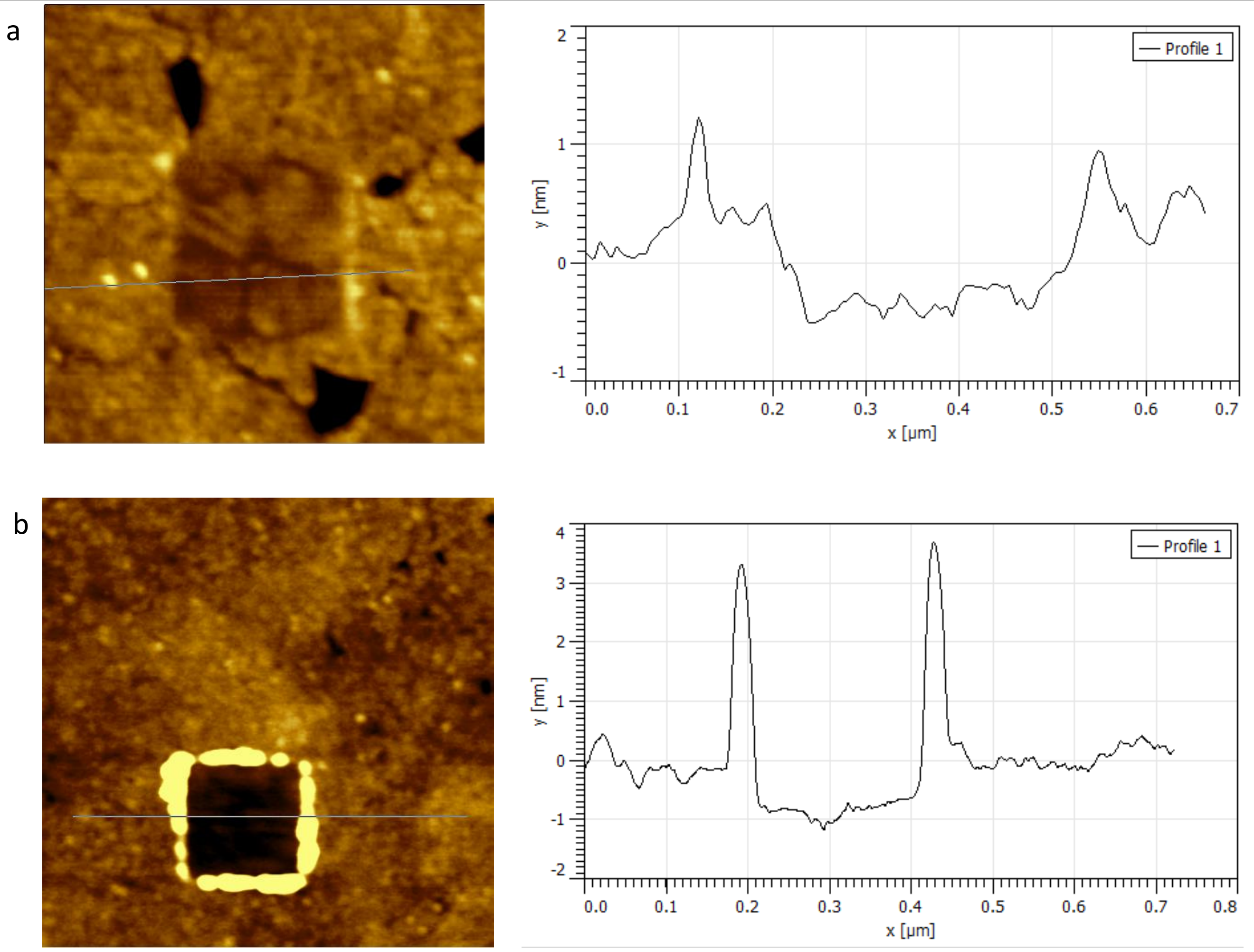

Figure S2.06. Nano-scratching of SAMs on template-stripped gold, with measured cross section data (grey line in images): a) **B*p*P**; b) **B(OPE3)*p*P**.

2.4 Reductive Desorption

Reductive desorption was performed using a CHI 660A potentiostat (CH Instruments). SAMs were prepared on template-stripped gold as described above. Aqueous 1 M KOH was used as the electrolyte, a Pt wire was used as the counter electrode and a SCE was used as the reference electrode. Data analysis was conducted using EC4-View software. The dy/dx = 0 point of the desorption voltammogram was taken as the desorption potential.

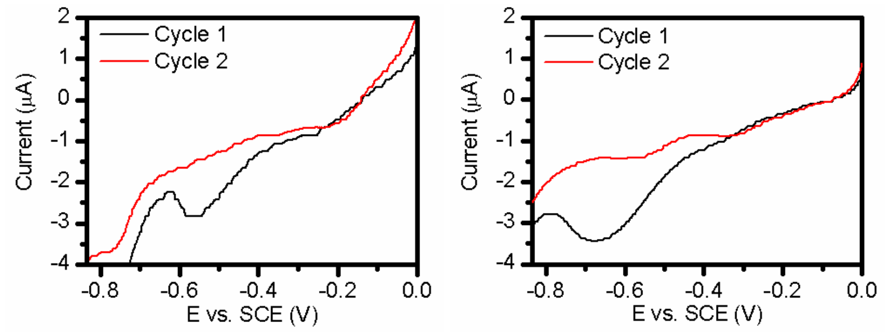

Figure S2.07. Desorption voltammograms of **BB** (left) and **PB** (right). Black curve: 1st cycle; red curve: 2nd cycle.

2.5 Quartz Crystal Microbalance

QCM measurements used a Novatech Strl QCM and polished evaporated gold QCM substrates (International Crystal Manufacturing, USA). To investigate SAM growth, the initial resonance frequency, f_0, of a substrate (which had been washed sequentially with acetone, methanol, then isopropanol, dried under a flow of nitrogen then exposed to oxygen plasma for 5 min) was first recorded. The substrate was then used as a surface for SAM growth (growing conditions same as above) and the frequency of the SAM-coated substrate was recorded, using a value after >20 min to ensure equilibration of the QCM. The resulting difference in frequency implies the number of molecules adsorbed on the substrate surface via Sauerbrey analysis.[24]

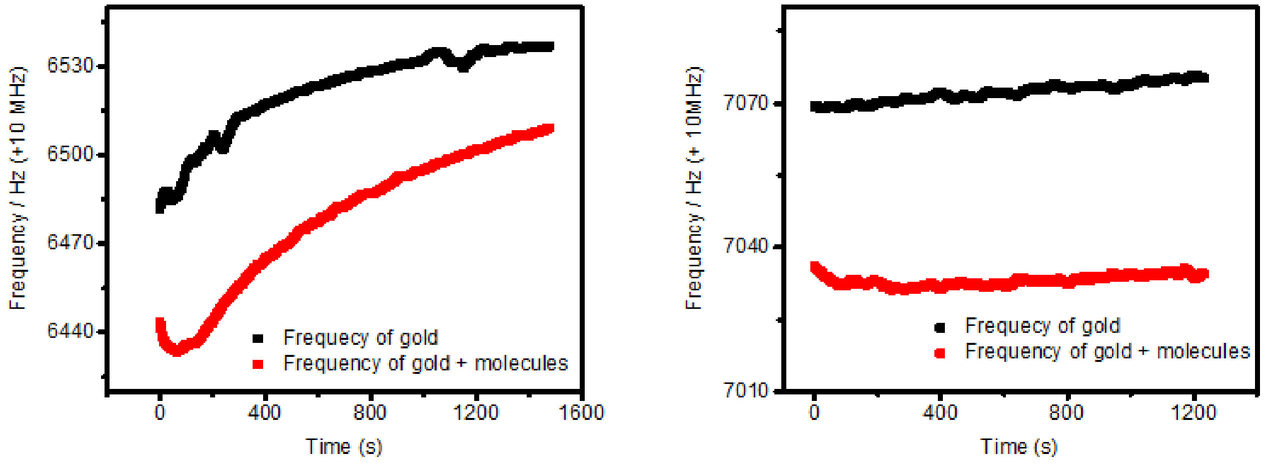

Figure S2.08. QCM frequency vs. measurement time before (black) and after (red) growth of SAMs of **BB** (left) and **PB** (right).

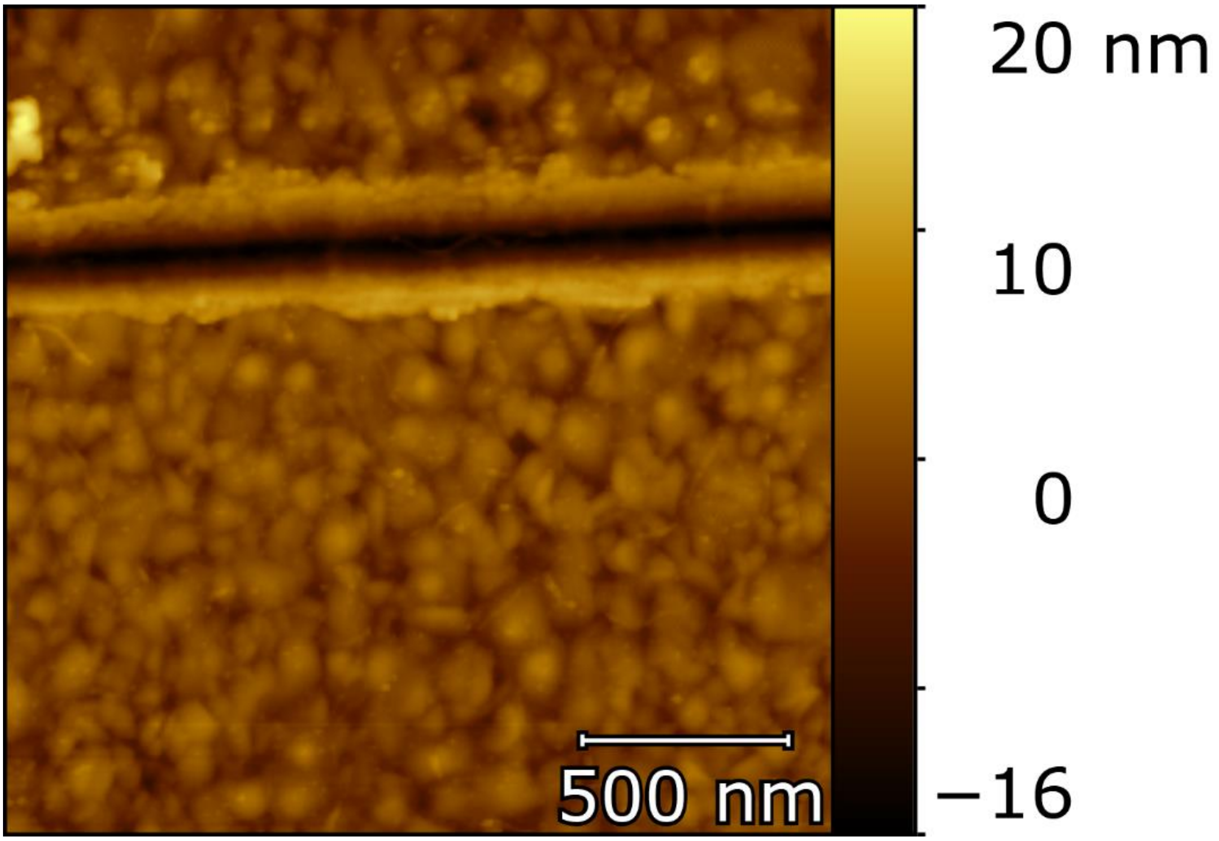

Figure S2.09. AFM image of the QCM surface. The rough surface allows for more molecules to bind per unit area relative to template stripped gold.

2.6 Conductive AFM

Conductive AFM (cAFM) studies used the Multi-Mode 8 (Bruker) instrument in conjunction with a Femto current pre-amplifier. Current maps were obtained in peak force mode (peak force setpoint at 1.4 nN). The collected signal was modulated by the z-direction motion of the piezo inside the microscope with a lock-in amplifier (SR830, SRS). IV characteristic curves were measured by cAFM in contact mode. A brand new probe was held at several points in different regions of the sample for IV curve collection. No in-plane motion was performed during the IV measurement to maintain the probe shape and the contact area between probe and sample.

To prepare graphene coated AFM probes, commercially available Pt/Cr cAFM probes (Multi-75E, Budget Sensors) were coated with high quality conformal few-layer graphene films using a Langmuir-Schaefer (LS) technique. 200 µL of graphene flake dispersion in dimethylformamide (Sigma-Aldrich, 0.2 mg/ml, 1-3 layers) was evenly spread on a water subphase of a 750 cm^2 Langmuir trough (KSV NIMA, Biolin Scientific) by micro-syringe drop – it is estimated that each droplet was approximately 15 µL. The dispersed graphene film was compressed at a rate of 5 mm/min to a solid-phase surface pressure of 8-10 mN/m to produce a densely packed graphene film. The compressed films were found to have a thickness in the range of 3-6 nm. For graphene coating, probes were fixed onto a silicon wafer with a Gel-Pac (grade 8) adhesive tab and contacted in the LS manner to the compressed film using the trough's automatic dipper. The coated probes were dried in a vacuum oven (10^{-2} mBar, 80 ˚C) for 12 h to remove any residual solvents.

Scanning electron microscopy (SEM) was used to characterise the quality of the graphene coated probes. Comparison of the clean, new probe before (Figure S2.10a) and after coating (Figure S2.10b) shows the presence of some surface wrinkles confirming the adhesion of the graphene sheets to the probe surface but, crucially, with no significant change of the curvature radius of the probe (Figure S2.10c, radius ~ 15 nm).

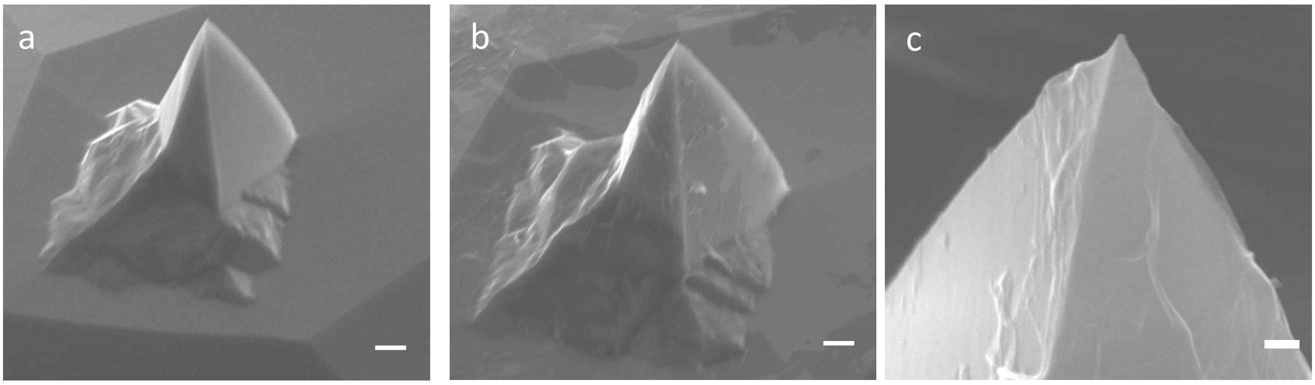

Figure S2.10. a) SEM image of a conductive AFM probe (scale bar 1 µm); b) SEM image of a conductive AFM probe coated with graphene (scale bar 1 µm); c) an enlarged image of (b) (scale bar 100 nm).

The contact area between the substrate and the probe was estimated using the Hertz model[25]:

$$r = \left(F \times R \times \frac{1}{Y}\right)^{\frac{1}{3}}$$

$$\frac{1}{Y} = \frac{3}{4} \times \left(\frac{1 - v_1^2}{E_1} + \frac{1 - v_2^2}{E_2}\right)$$

Where *r* is the real contact area, *F* is the loaded force between the sample and the probe, *R* is the tip radius (as quoted by the supplier and further confirmed by SEM imaging), v_1 *and* v_2 are the Poisson ratios[26] of the probe and sample, respectively, and E_1 and E_2 are the Young's moduli of the probe[26] and sample, respectively. E_2 was determined from nanomechanical mapping using the AFM in peak force mode.[27]

The ratio between the calculated value of *r* and the molecular area (as estimated from the reductive desorption studies) was used as the number of molecules in a given junction. This value then allows determination of the conductance contribution of a single molecule (G_M) from the junction conductance G_J. We note that this assumes that all molecules in the junction form contacts with the top probe. If this is not the case, the conductance contribution of those molecules in contact with the probe would be higher, and thus the calculated G_M values are lower bounds for the conductance of molecules in a junction.

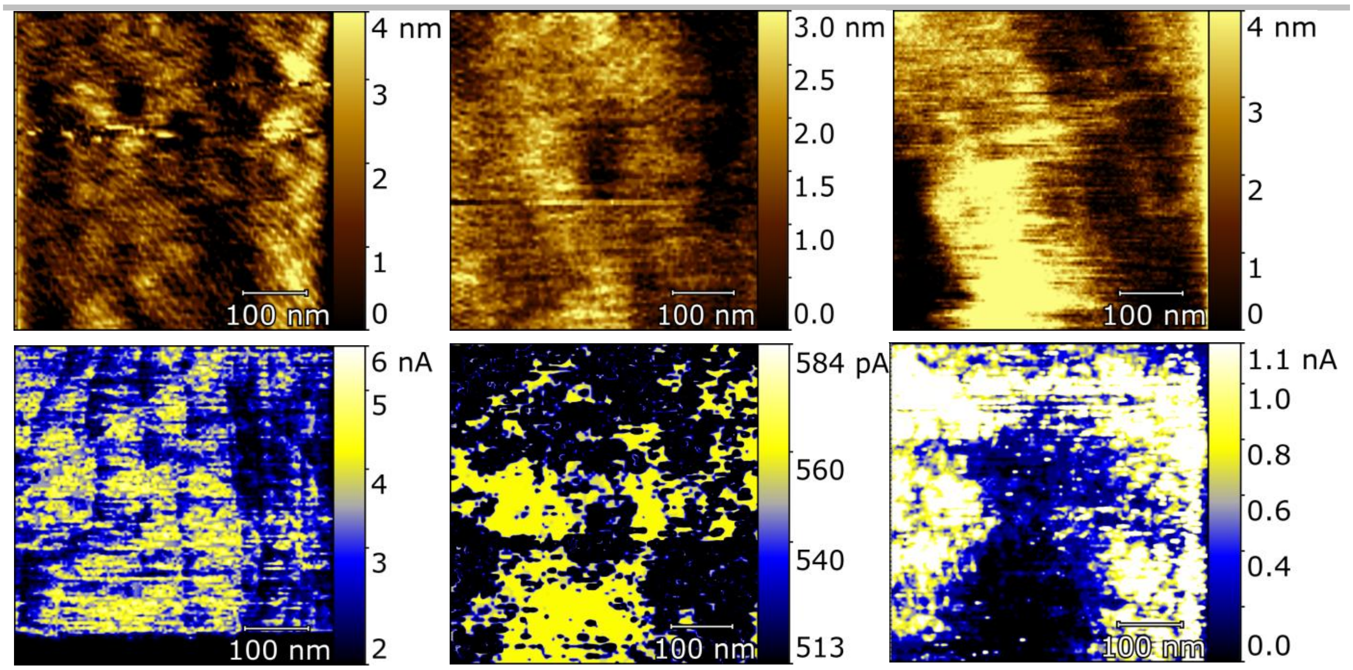

Figure S2.11. AFM topography (above) and cAFM electrical map (below) of SAMs of left: **BB**; centre: **B(OPE3)*p*P**; right: **P*p*P**. Scan area: 500 nm × 500 nm. Loaded force 2 nN.

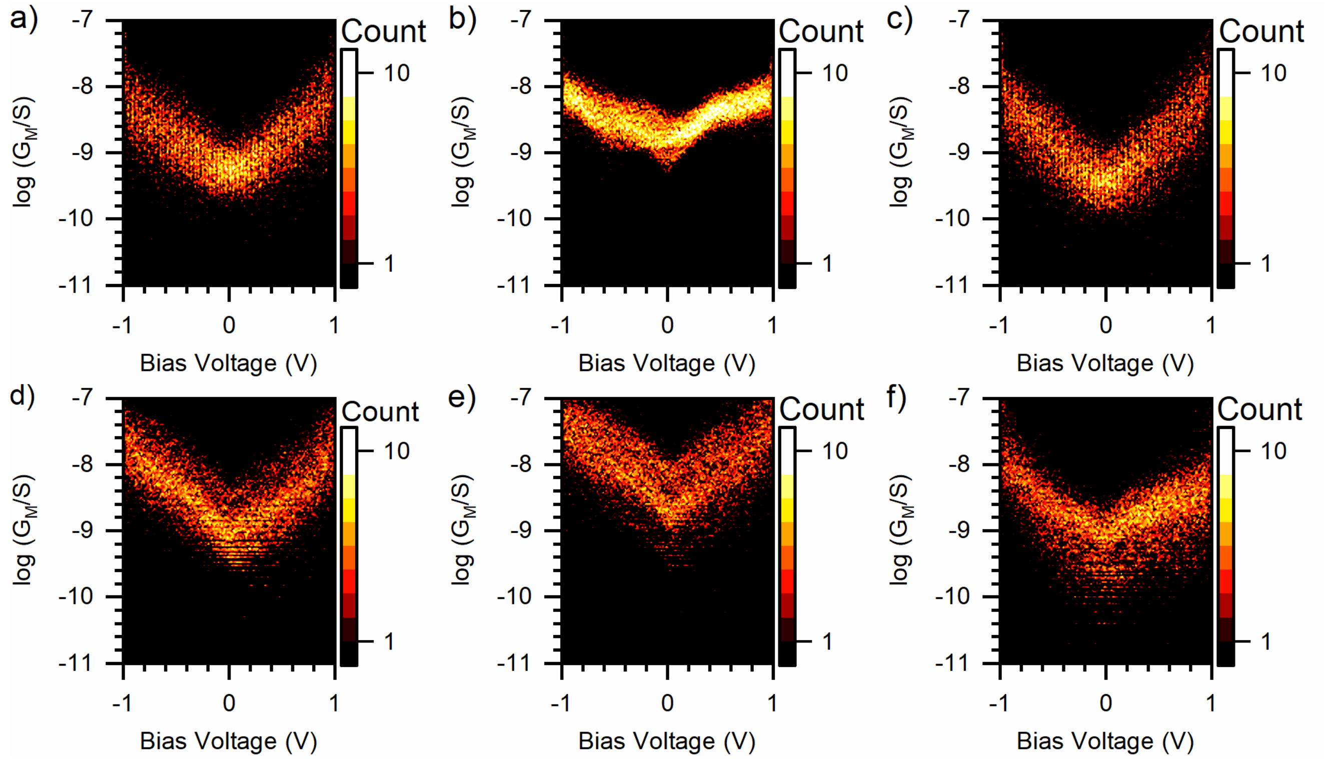

Figure S2.12. Differential conductance versus voltage for SAMs of tetrapodal OPE2 wires on template-stripped gold from cAFM with a Pt probe: a) **BB**; b) **B*p*P**; c) **B*m*P**; d) **PB**; e) **P*p*P**; f) **P*m*P**.

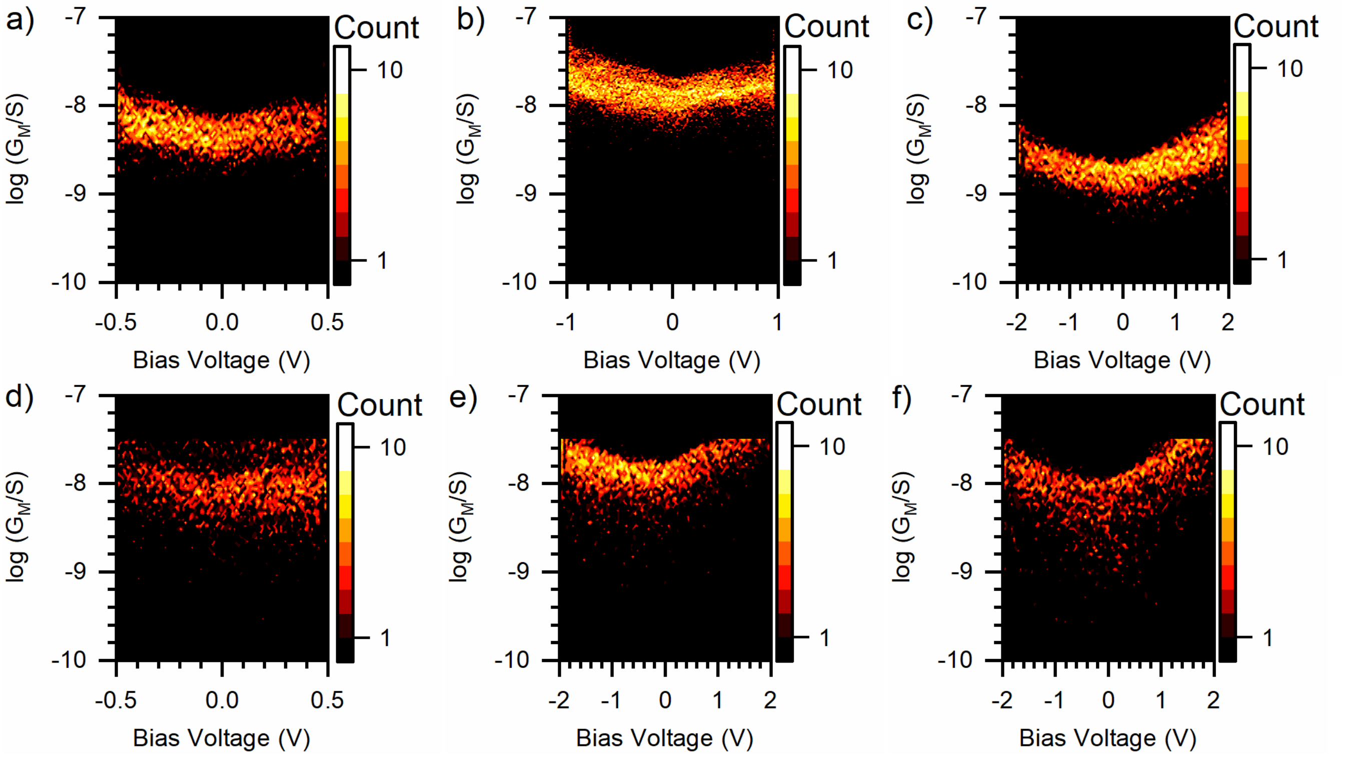

Figure S2.13. Differential conductance versus voltage for SAMs of tetrapodal OPE2 wires on template-stripped gold from cAFM with a graphene-coated probe: a) **BB**; b) **B*p*P**; c) **B*m*P**; d) **PB**; e) **P*p*P**; f) **P*m*P**.

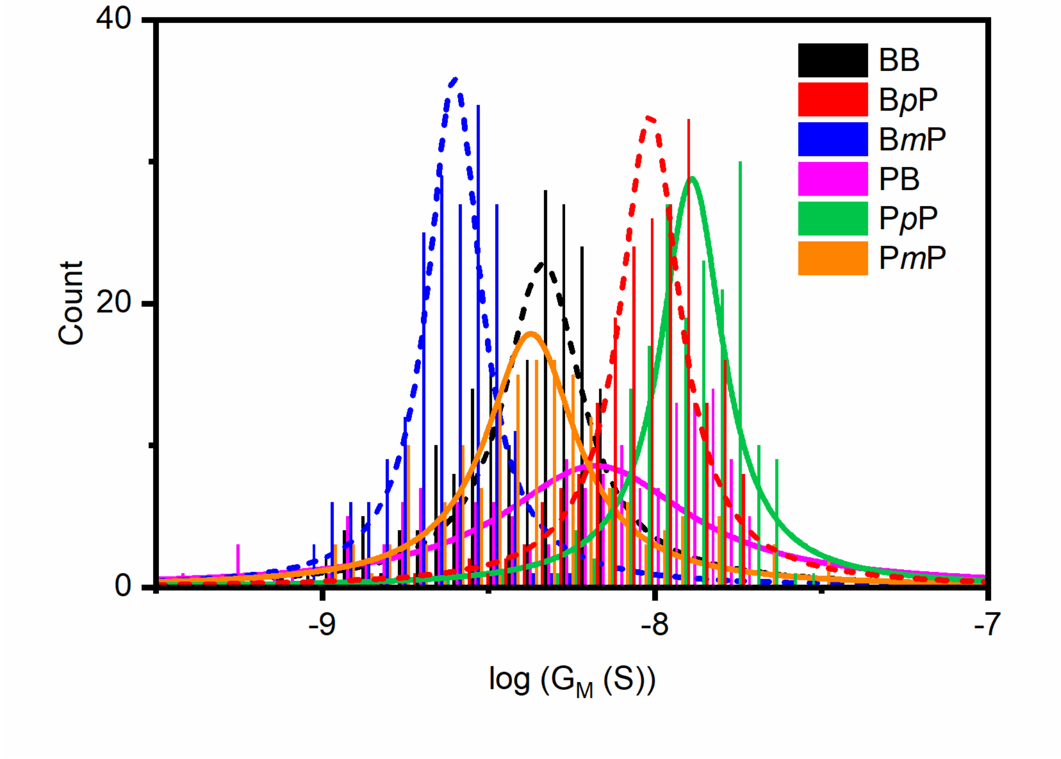

Figure S2.14. Histograms of conductance per molecule (G_M) for Au-SAM-Graphene junctions containing OPE2 tetrapodal molecular wires at low bias voltage (−0.1 V to 0.1 V). Fit curves are a guide for the eye.

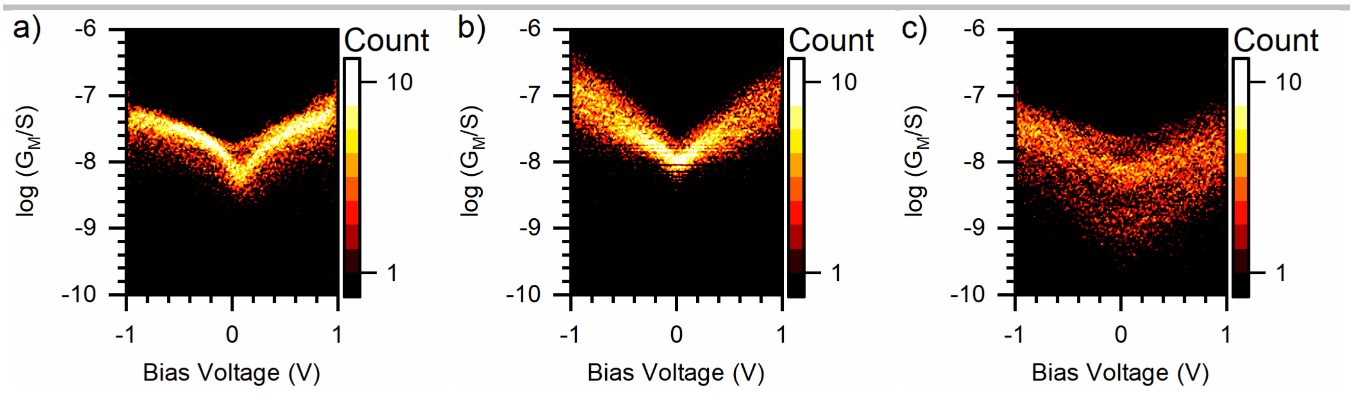

Figure S2.15. Differential conductance versus voltage for SAMs of OPE2 monothiol wires on template-stripped gold from cAFM with a Pt probe: a) **SB**; b) **S*p*P**; c) **S*m*P**.

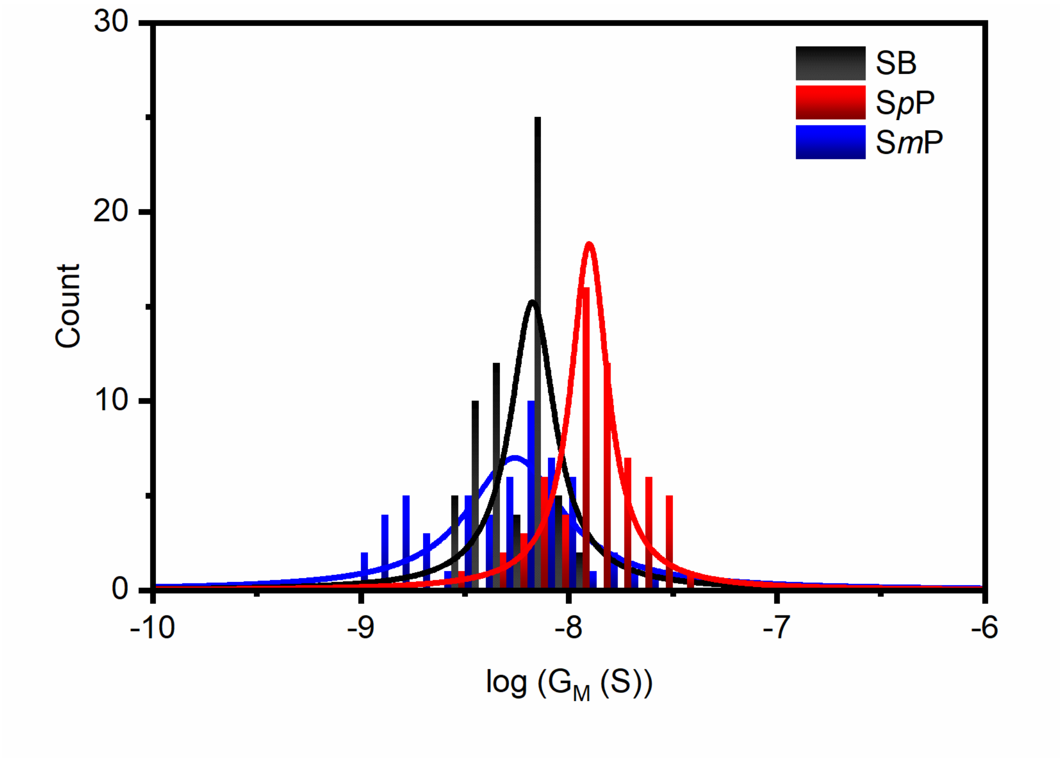

Figure S2.16. Histograms of conductance per molecule (G_M) for Au-SAM-Pt junctions containing OPE2 monothiol molecular wires at low bias voltage (−0.1 V to 0.1 V). Fit curves are a guide for the eye.

Figures S2.12, S2.13, S2.15 (above) and Figure 2c in the main text all show the effect of bias voltage on the differential conductance of the investigated molecular wires. As the wires are asymmetric and the electrodes are made of different materials, it is reasonable to expect these curves to display asymmetry. However, in most cases the curves are largely symmetric (although some clear asymmetry can be seen, e.g. in Figure 2.12f). If, as the charge transport calculations suggest, the carbazole units are not involved in the conductive pathway and electrons travel directly from the gold electrode to the OPE backbone of the tetrapodal wires, the conductive pathway is actually relatively symmetrical (particularly if π-probe interactions dominate at the top electrode). Largely symmetric I-V curves (in the region −1 V to 1 V) were previously reported for simple symmetric OPEs in cAFM junctions where the electrode materials are different (fully symmetric curves were obtained if both electrodes were made of gold).[28] Additionally, reasonably symmetric I-V curves were reported for asymmetric molecular wires in cAFM junctions using a gold bottom electrode and gold-coated probe.[14c] The tendency to symmetry observed in this work is therefore consistent with previous cAFM studies.

2.7 SAM Stability

The stability of SAMs of materials with tetrapodal anchors was investigated by monitoring the thickness, roughness and conductance of **BB** SAMs over 6 days. **BB** SAMs were prepared as described above. The characteristics of a SAM were determined just after preparation, and two SAMs were stored for 6 days, one in a vacuum chamber (10^{-3} mBar) and the other in ambient air. Neither of the stored SAMs was shielded from ambient light. The characteristics of the stored SAMs were measured after 1, 2, 3 and 6 days in storage, with little variation. Before each measurement, the SAMs were incubated in a vacuum chamber for 2 hours to minimize the effect of water. The properties measured at the beginning and end of the study are recorded in Table S3.

Table S3. Thickness, roughness and conductance of **BB** SAMs immediately after preparation or after storage under vacuum or in ambient air.

Sample	Thickness (nm)	Roughness[a] (nm)	log (G_M (S))	std
After Preparation	0.51-0.74	0.67	–8.85	0.59
After 6 days in vacuum	0.54-0.69	0.66	–8.8	0.59
After 6 days in air	0.59-0.71	0.68	–8.72	0.75

[a]mean deviation from the middle plane, averaged over several randomly selected areas of the SAM.

Based on the absence of any significant change in the SAM thickness or roughness, there is no evidence of SAM degradation after 6 days either in vacuum or ambient air. As seen in the representative images in Figure S2.17, the stored SAMs also appear similar to the as-prepared SAM. The average molecular conductance of the three SAMs is also similar*, although the SAM stored in air affords a broader conductance distribution than the as-prepared sample (see Figure S2.18). This may be due to the formation of a water film on the sample surface, which would affect the contact interface between the sample and the cAFM probe. Taken together, these results indicate that the SAMs of **BB** remain stable for at least 6 days. In contrast, previous studies using XPS have indicated that simple alkanethiol SAMs on Au show significant oxidation after only 1 day in ambient air, with complete oxidation within a week and an indication that some sulfur-containing species are lost from the surface in this time.[29]

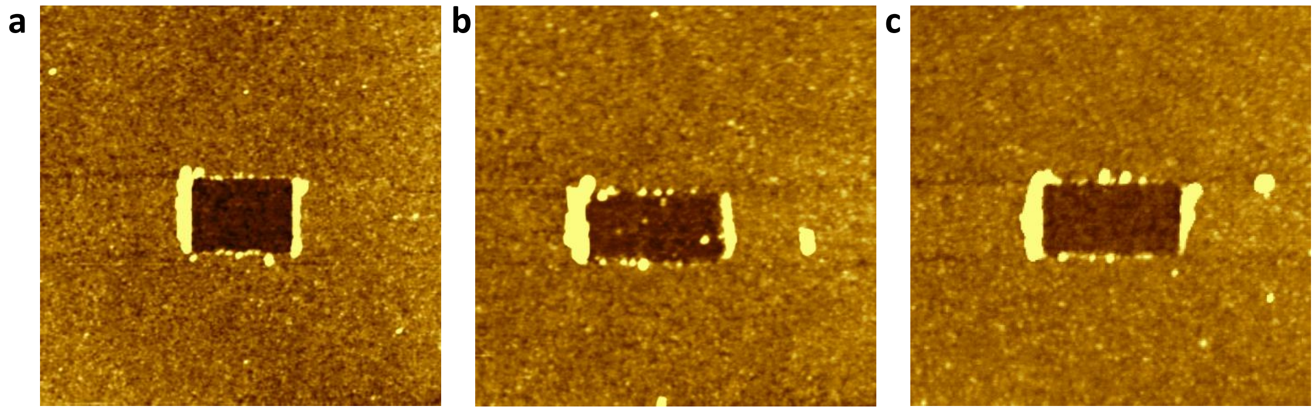

Figure S2.17. AFM topography and nano-scratching window of SAMs of **BB:** a) immediately after preparation; b) after storage in vacuum (10^{-3} mBar) for 6 days; c) after storage in ambient air for 6 days.

* The conductance obtained in this study differs slightly from that reported in the main text. This is attributed to variations between the cAFM probes used for measurements conducted at different times.

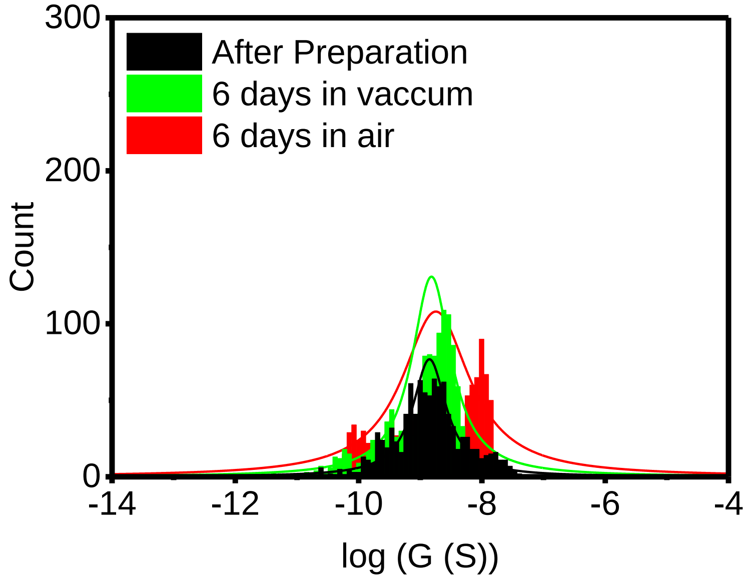

Figure S2.18. Distributions of electrical conductance per molecule (G_M) for Au-SAM-Pt junctions containing **BB**, either immediately after preparation or after storage under different conditions.

3. Computational Studies

3.1 Geometry Optimisation

Structural relaxations of the tetrapod molecules were carried out using the SIESTA[30] implementation of density functional theory (DFT) with the LDA CA[31] functional. The mesh cut-off was 200 Rydberg and a double zeta polarised basis set was used for all atoms. Structural relaxations were performed to a force tolerance of 0.01 eV/Å. Geometries of model systems relaxed in this manner in the gas phase and on Au(111) are included in the main text (Figure 1). In addition to the geometries on Au(111) where the head group is tilted away from the surface (Figure S3.01, left), it is also possible for the whole molecule to lie flat against the Au surface, as shown in Figure S3.01 (right) for the **BB** tetrapod. The binding energy for this flat configuration is -1.82 eV, compared to -1.85 eV when tilted. We note that in addition to having a slightly higher binding energy, the tilted structure is consistent with the experimentally determined film thickness. All calculated binding energies are lower bounds of the actual value as they are based on local-density approximations and do not include van der Waals interactions, which would increase the binding energy further.

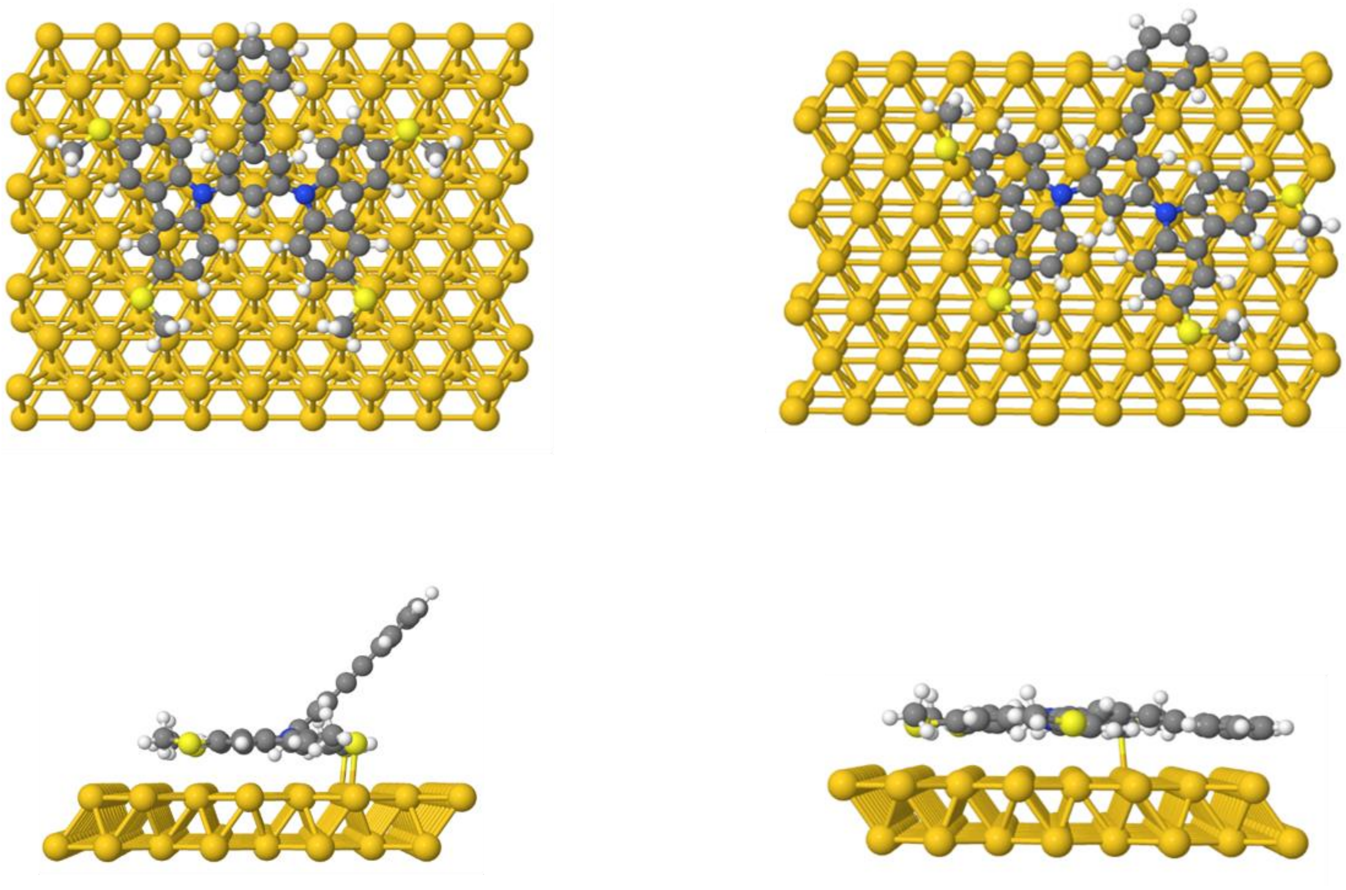

Figure S3.01. Two relaxed configurations for **BB** tetrapod on Au(111), each viewed from above and perpendicular to the surface. *Left:* tilted configuration, with binding energy of -1.85 eV. *Right:* planar configuration with binding energy of -1.82 eV.

To assess the contribution of each SMe group to the total binding energy, additional relaxations of a model, alkyne-terminated benzene-based tetrapod were performed in which one or more SMe groups were replaced by hydrogen atoms. The results are shown in Table S4 and indicate that each SMe group contributes ca. −0.3 eV to the total binding energy. The compound with no SMe groups (i.e. a model of **1**) has a calculated binding energy of −0.54 eV, showing that the carbazole π-systems also contribute to binding (although, as observed experimentally and described in the main text, not sufficiently for **1** to form a SAM on Au(111)).

Table S4. Binding energies on Au of various tetrapod molecules where one or more SMe groups have been replaced by H atoms.

**Structure**	**Binding energy on Au (111) surface (eV)**	**Structure**	**Binding energy on Au (111) surface (eV)**
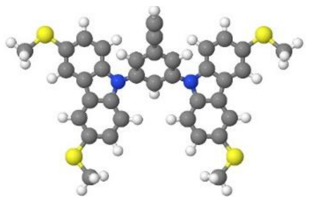	−1.86	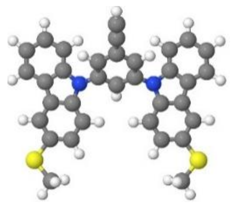	−1.15
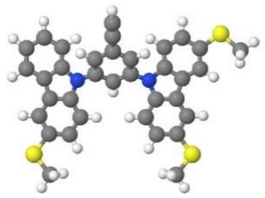	−1.51	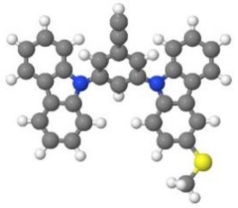	−0.84
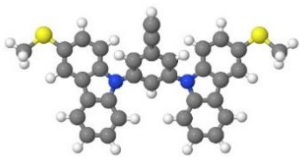	−1.29	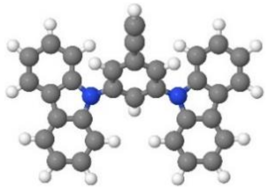	−0.54

As an approximation of the surface area occupied by a single tetrapod molecule on Au it is assumed that each molecule covers a rectangle with length and width defined by the positions of the four S atoms, with a spacing of 5 Å between these rectangles in both directions. This gives a footprint of 265 $Å^2$ for both the benzene and pyridine based molecules.

3.2 Charge Transport Studies

Electron transport through the molecules was investigated using the Green's function transport code Gollum[32] based on the DFT Hamiltonians and overlap matrices. From these the transmission coefficient as a function of energy E is:

$$T_{12}(E) = Tr\ (\Gamma_1 G \Gamma_2 G^{\dagger})$$

where G is the (retarded) Green's function of the junction and Γ_{i} is the imaginary part of the self energy of electrode i. The conductance is then[33]:

$$G = G_0 \int_{\infty}^{\infty} T_{12}(E) \left(-\frac{\partial}{\partial E} f(E)\right) dE,$$

where $f(E)$ is the Fermi distribution function. The temperature was set to 300 K. For these calculations the PBE[34] functional was used. A double zeta basis set was used for Au atoms and a double zeta polarised basis set for all other atoms.

A molecular junction was assembled with a single **BB** molecule placed between Au(111) leads spaced 11.9 Å apart. This distance was chosen based on the vertical height of the relaxed molecule on the Au surface, and the optimum distance from the top contact determined from a separate relaxation. The molecule was allowed to relax in the junction with the leads held fixed, and the resulting geometry (Figure 3a in the main text) was used to calculate the transmission function. To investigate the effect of the carbazole-based anchoring groups, they were removed from the relaxed structure and a transmission function was calculated for the conductive backbone only, held in the same geometry as for **BB** (Figure 3b in the main text). Figure 3c in the main text shows the two transmission functions. Apart from a small change in the Fermi energy, the transport is very similar, establishing that the backbone of the molecule forms the conductance pathway, with the anchor groups playing no significant role in conductance.

Figure S3.02 shows the variation of transport with molecular length for the series **B(OPE1)*p*P**, **B*p*P (i.e. B(OPE2)*p*P)**, **B(OPE3)*p*B** (described in the main text) and their extended analogues **B(OPE4)*p*B** and **B(OPE5)*p*B** (Figure S3.03, not synthesised). Assuming mid-gap transport with a Fermi energy 1 eV below the DFT predicted Fermi energy, the conductance for the series **B*p*P** – **B(OPE5)*p*P** follows the pattern $G = G_C e^{-\beta d}$ where d is the length of the molecular backbone and the attenuation factor β is 2.1 nm^{-1}. **B(OPE1)*p*P** falls outside of this pattern, as the short conductive backbone (a single pyridine ring) means that there is significant coupling between the top lead and the carbazole units, leading to an increased conductance. When coupling between the carbazole units and the top

contact is removed by setting the corresponding Hamiltonian and overlap matrix elements to zero, the resulting transmission function and conductance value lie closer to the trends observed for the longer analogues (see red curve in Figure S3.02a, and red marker in Figure S3.02b). The resulting conductance trend shows better agreement with that observed for experimentally determined values.

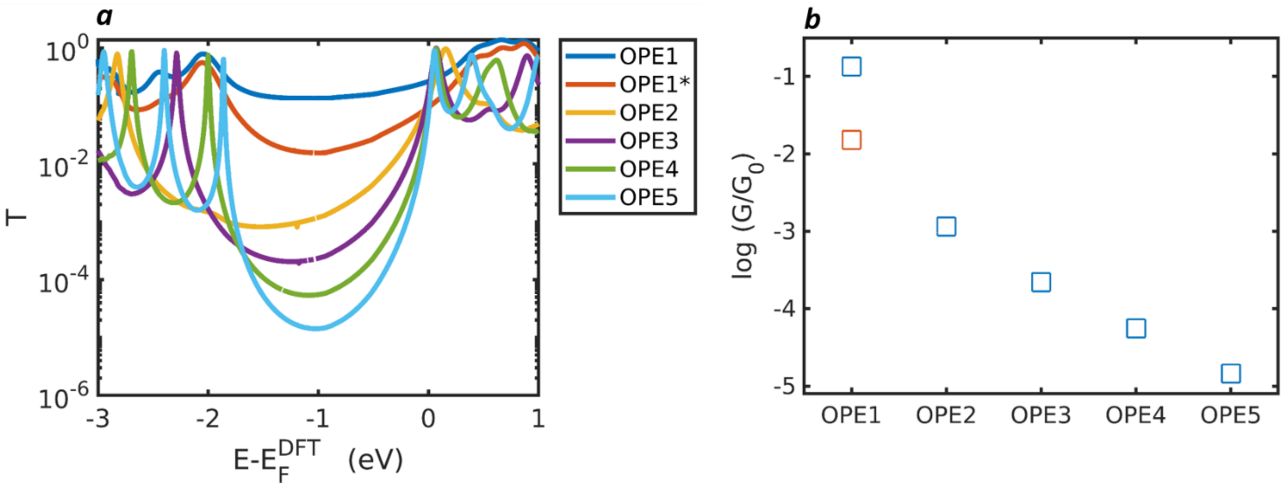

Figure S3.02: a) Transmission functions and b) conductance (at $E - E_F^{DFT} = -1$) for the "OPE1-5" series of tetrapods with *para*-pyridyl head groups **B(OPE1)*p*P**, **B*p*P** , **B(OPE3)*p*P**, **B(OPE4)*p*P** and **B(OPE5)*p*P**. The red transmission curve (marked with an asterisk) in a) is for a **B(OPE1)*p*P** molecule where all parts of the molecule except for the pyridyl backbone have been decoupled from the top electrode. The corresponding conductance is coloured red in b).

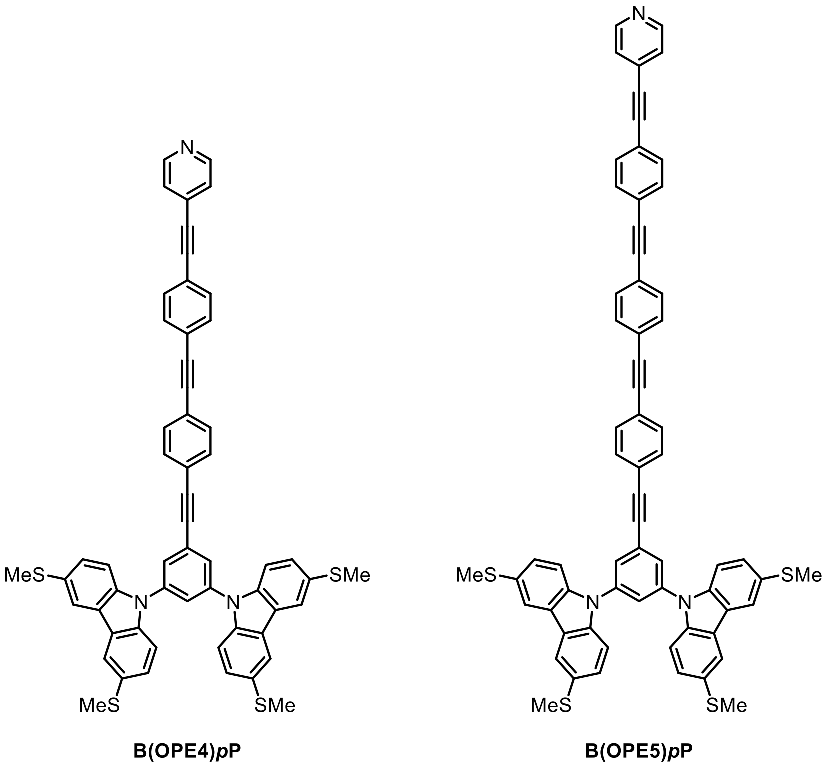

Figure S3.03: Structures of the longer tetrapod analogues investigated in the charge transport simulations.

Transmission functions for each of the tetrapods in the "OPE2" series (**BB**, **B*p*P**, **B*m*P**,**PB**, **P*p*P**, **P*m*P**) are shown in Figure S3.04. The separation of the leads is 11.9 Å in each case. The obtained curves are all similar and based on this the molecular conductance values for each molecule are expected to lie within an order of magnitude, as is found experimentally. However, the details of any trends in the calculated conductance are sensitive to small changes in the position of the Fermi energy, which cannot be reliably determined from DFT calculations. Furthermore, other factors affecting conductance are not taken into account in these simulations, such as any roughness in the leads, the presence of any contaminants in the junction and any variation in the geometry of the molecules (e.g. tilt angle) or junctions (e.g. tip-molecule contact configuration). Although the experimental conductance results are consistent with constructive (***p*P**) and destructive (***m*P**) quantum interference (QI), anti-resonance features associated with destructive QI are not seen in the simulations for tetrapods with ***m*P** head groups. For short molecules, transport through σ-orbitals can be comparable with that

through π-orbitals and mask destructive QI.[35] If the junction geometry used in these calculations permits significant contributions from σ-transport, the expected QI features could be hidden.

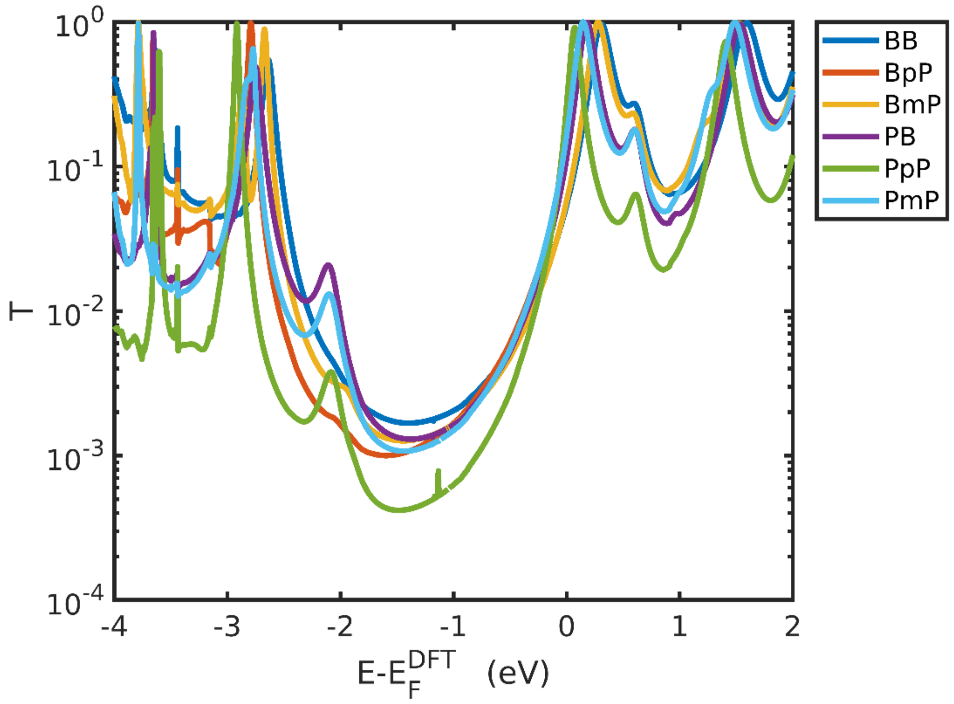

Figure S3.04 Simulated transmission functions for the six "OPE2" tetrapod molecules.

To investigate the conductance variation between different the head groups, calculations were performed on the model systems tolane and 4-(phenylethynyl)pyridine. These correspond to the conductive backbones of **BB** and **B*p*P** respectively. Based on our observations above (see also Figure 3 of the main text), these model systems are expected to be representative of the analogous tetrapods, but allow for reduced computational load. Figure S3.05 shows the transmission curves for these molecules placed in molecular junctions at a tilt angle of 45°, with the height of the top Au contact above the uppermost C or N atom varying between 2.5 and 3.5 Å. In each case the base of the molecule is held in the same position, with the C atom at the base of the lower phenyl ring placed 3.0 Å directly above a Au atom on the bottom lead. These results show that for a given height of the top lead, the molecule with the benzene head would be expected to have higher conductance than the pyridine head (at and around $E - E_F^{DFT} \approx -1$).

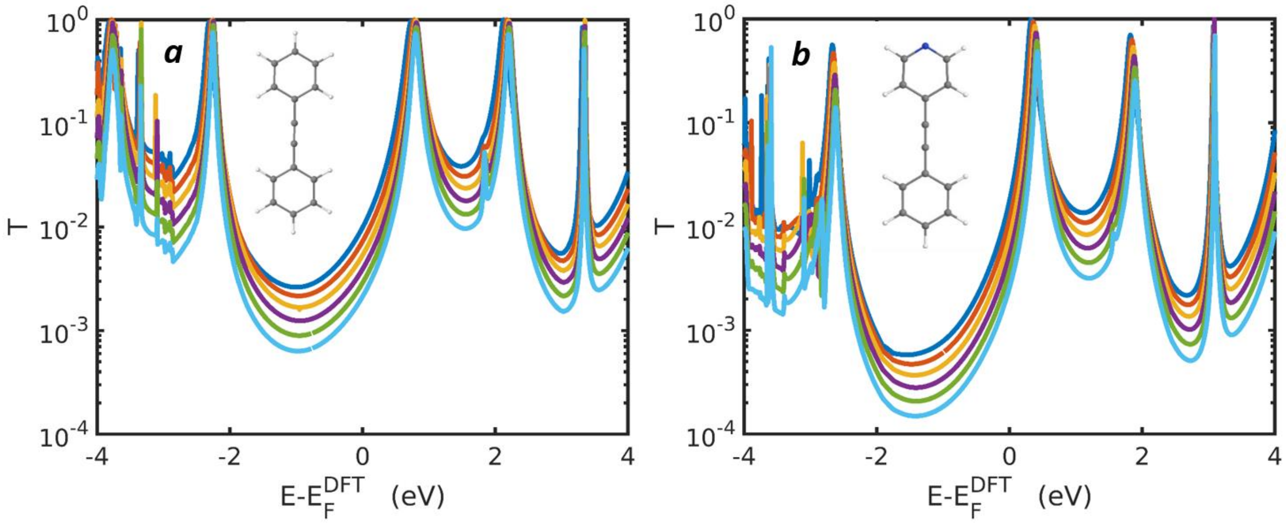

Figure S3.05 Transmission curves for a) tolane (benzene head) and b) 4-(phenylethynyl)pyridine (*para*-pyridine head) as the height of the top Au contact above the molecule is varied from 2.5 Å (dark blue curve) to 3.5 Å (light blue curve) in increments of 0.2 Å. The tilt angle is 45°.

In Figure S3.06 the binding energies of benzene and pyridine as a function of their height above a Au(111) sheet are plotted for various tilt angles. The binding energy of pyridine is generally larger than benzene, particularly for large tilt angles, with a minimum closer to

the Au surface than benzene. The binding energy minimum for benzene is more sensitive to the tilt angle due to the steric hindrance effect of the attached hydrogen atom.

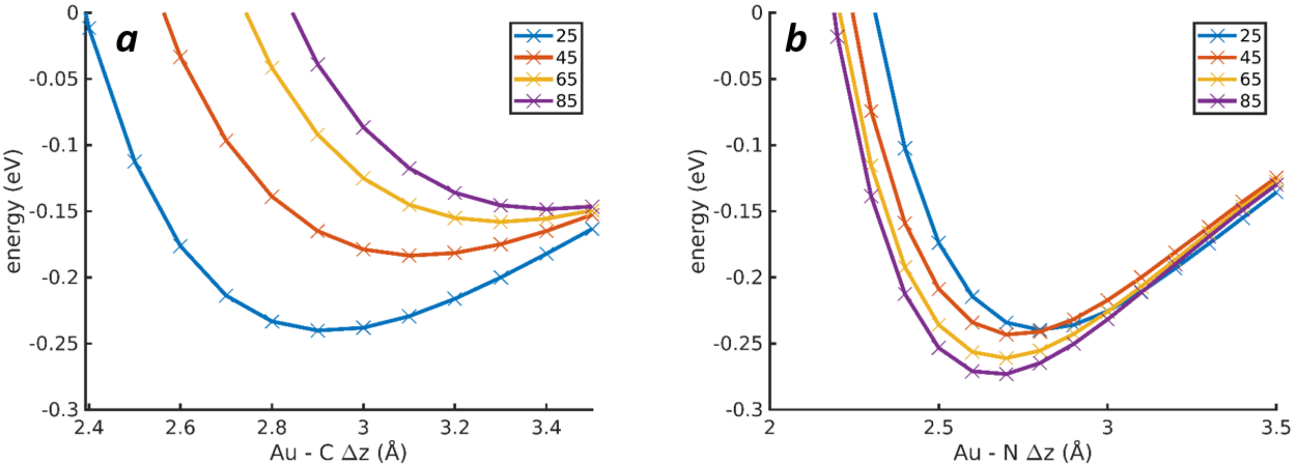

Figure S3.06: Binding energy as a function of distance for a) benzene and b) pyridine on a Au(111) sheet, for various tilt angles (degrees) between the molecule and the plane of the Au surface.

The positions of the binding energy minima from the curves in Figure S3.06 were used to set the height of the top contact on a further series of transport calculations, shown in Figure S3.07. Even taking into account the fact that pyridine headed molecules tend to lie closer than the benzene headed molecules, the conductance for a given tilt angle is still higher for the benzene headed molecule (at and around $E - E_F^{DFT} \approx -1$).

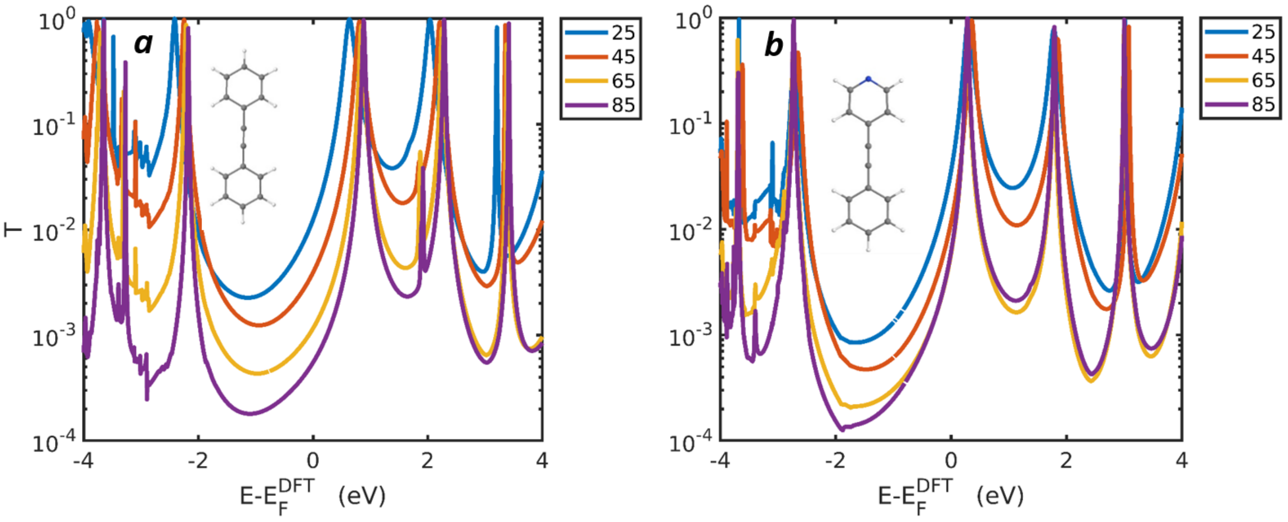

Figure S3.07 Transmission curves for a) tolane (benzene head) and b) 4-(phenylethynyl)pyridine (*para*-pyridine head) at various tilt angles (degrees) with respect to the Au plane. The height of the top Au contact is determined from the minima of the binding energy curves in Figure S3.07.

Similar calculations were performed with junctions involving leads with Au tips. Three configurations were considered, with differing angles between the plane of the molecule and the vector joining the uppermost C/N atom and the Au tip (Figure 3.08a). In position 1, this angle is 0°, corresponding to a geometry where electrons are injected into the σ system of the aromatic ring. In position 3, the angle is 90°, corresponding to injecting into the π system. Position 2 is intermediate, with an angle of 45°. The minima of the binding energy curves in Figure 3.08b-c were used to place the molecules for the transport calculations in Figure 3.08d-e. The binding energies to these undercoordinated Au atoms are significantly larger for pyridine headed molecules, particularly for position 2 which is the most strongly conducting configuration. These calculations highlight the importance of junction geometry when comparing our tetrapodal molecules in junctions; for a benzene head group, the largest binding energy is associated with position 3, whereas for a pyridine head group it is associated with position 2. Comparing the transmission functions for the most favoured binding positions of the two species (yellow curve, Figure S3.08d and orange curve, Figure S3.08e), very little difference can be seen in the region $0 \geq E - E_F^{DFT} \geq -1$. As discussed above, based on this observation the experimental conductance values for the corresponding molecules are expected to be within an order of magnitude, which is indeed the case. Although in this case some aspects of differences in junction configuration are accounted for, these calculations still do not account for the "true" position of the Fermi energy or the preferred tilt angle of the two molecules within the junction, amongst other factors.

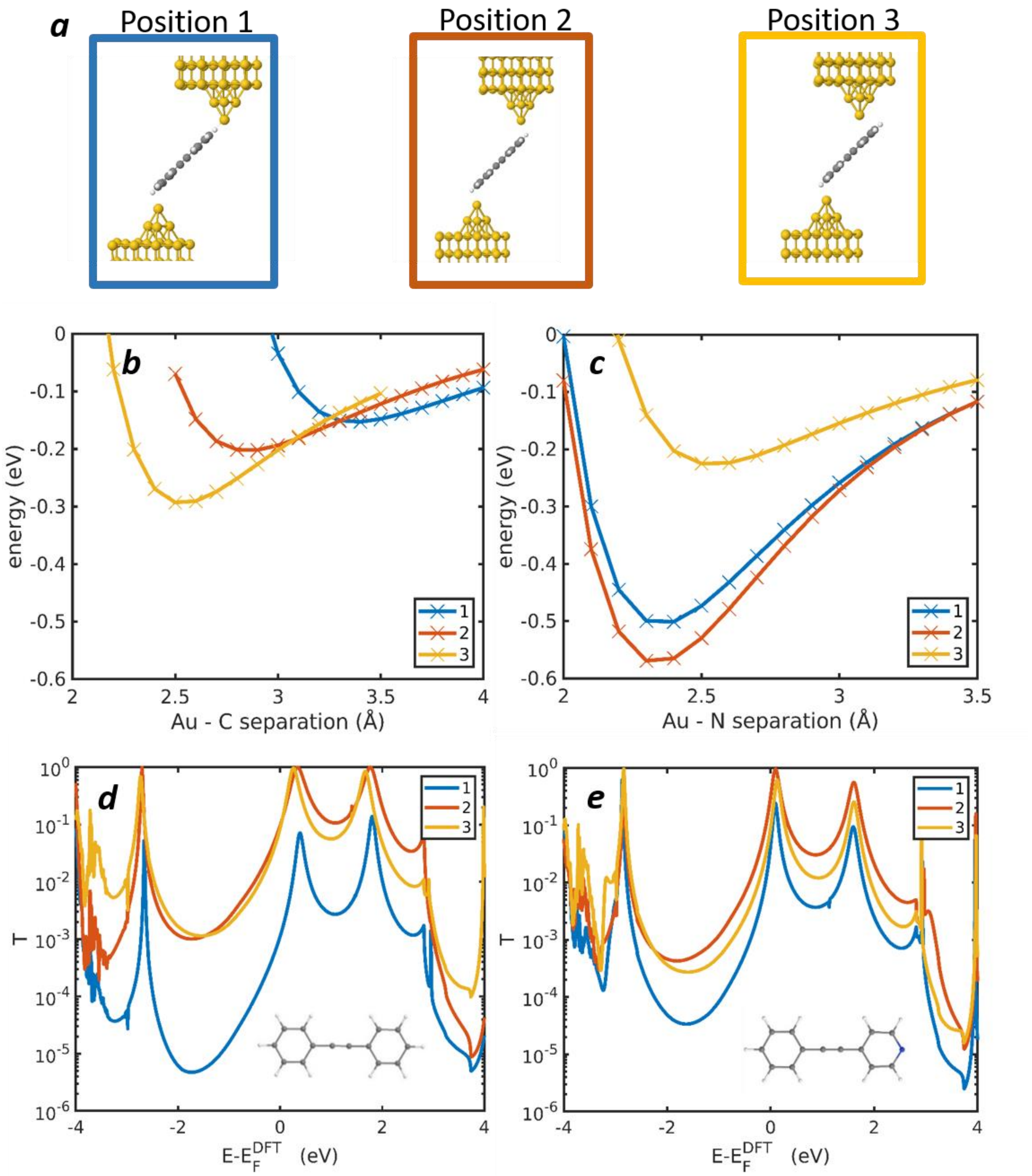

Figure S3.08 a) Three configurations for coupling to a top contact with a tip; b) and c) binding energies to Au tip as a function of distance for benzene and pyridine molecules, respectively, in positions 1, 2 and 3; d) and e) Transmission curves for tolane (benzene head) 4-(phenylethynyl)pyridine (*para*-pyridine head) in configurations from a) where the distance between Au and C/N has been set according to the position of corresponding the binding energy minimum in b) or c).

Another complicating factor when comparing theory and experiment is that the proportion of molecules in contact with the cAFM probe in the conduction experiments may be less than the assumed 100% of those in the contact area. If not all molecules contact the probe, the ratio in contact may depend on the nature of the head group. We have shown above that pyridine has a higher binding energy to gold than benzene, suggesting that pyridine headed molecules are more likely to interact with a metallic probe (particularly in the presence of any undercoordinated adatoms) and contribute to the junction conductance. This could provide an alternative explanation for the experimental observation of higher conductances for junctions containing tetrapods with ***pP*** head groups than those containing

analogues with **B** head groups: rather than a higher conductance per molecule, it could be that a larger number of conductive molecules are contacting both electrodes in the ***p*P**-headed case. However, in this case, it would be reasonable to expect that ***m*P** head groups would be more likely to contact the top electrode than **B** head groups, potentially affording higher junction conductances despite the potential negative effects of QI, which is not observed experimentally. Additionally, similar trends are observed for both Pt and graphene cAFM probes, for which comparable head group-probe interactions are expected in all cases, as pyridine has been shown to preferentially interact with graphene through π-π overlap rather than its lone pair.[36] On this basis, we favour the interpretation that the proportion of molecules in contact with the cAFM probe is high, and similar for all structures. By assuming all molecules make contact, the G_M values represent a lower bound for conductance per molecule. The reasonable agreement between the cAFM results for **S*p*P** and **S*m*P** with literature MCBJ conductance values[37] further supports this interpretation.

References

[1] A. F. Burchat, J. M. Chong, N. Nielsen, *J. Organomet. Chem.* **1997**, *542*, 281-283.
[2] H. E. Gottlieb, V. Kotlyar, A. Nudelman, *J. Org. Chem.* **1997**, *62*, 7512-7515.
[3] M. Y. Wong, G. J. Hedley, G. Xie, L. S. Kölln, I. D. W. Samuel, A. Pertegás, H. J. Bolink, E. Zysman-Colman, *Chem. Mater.* **2015**, *27*, 6535-6542.
[4] C.-H. Chen, T.-H. Hu, T.-C. Huang, Y.-L. Chen, Y.-R. Chen, C.-C. Cheng, C.-T. Chen, *Chem. Eur. J.* **2015**, *21*, 17379-17390.
[5] A. Ken, M. Kenichi, F. Katsuhiko, Y. Kimihisa, *Angew. Chem. Int. Ed.* **2015**, *54*, 5677-5682.
[6] K. Kobayashi, E. Koyama, K. Namatame, T. Kitaura, C. Kono, M. Goto, T. Obinata, N. Furukawa, *J. Org. Chem.* **1999**, *64*, 3190-3195.
[7] N. I. Baranova, V. I. Shishkina, *Chem. Heterocycl. Compd.* **1971**, *7*, 1009-1010.
[8] L. Wang, N. Liu, B. Dai, *RSC Adv.* **2015**, *5*, 82097-82111.
[9] I.-Y. Wu, J. T. Lin, J. Luo, S.-S. Sun, C.-S. Li, K. J. Lin, C. Tsai, C.-C. Hsu, J.-L. Lin, *Organometallics* **1997**, *16*, 2038-2048.
[10] S. Wu, M. T. González, R. Huber, S. Grunder, M. Mayor, C. Schönenberger, M. Calame, *Nat. Nanotechnol.* **2008**, *3*, 569.
[11] D. T. Gryko, C. Clausen, K. M. Roth, N. Dontha, D. F. Bocian, W. G. Kuhr, J. S. Lindsey, *J. Org. Chem.* **2000**, *65*, 7345-7355.
[12] M. A. Bartucci, P. M. Wierzbicki, C. Gwengo, S. Shajan, S. H. Hussain, J. W. Ciszek, *Tetrahedron Lett.* **2010**, *51*, 6839-6842.
[13] M. A. Bartucci, J. W. Ciszek, *J. Org. Chem.* **2014**, *79*, 5586-5594.
[14] a) M. Valášek, K. Edelmann, L. Gerhard, O. Fuhr, M. Lukas, M. Mayor, *J. Org. Chem.* **2014**, *79*, 7342-7357; b) Y. Ie, T. Hirose, H. Nakamura, M. Kiguchi, N. Takagi, M. Kawai, Y. Aso, *J. Am. Chem. Soc.* **2011**, *133*, 3014-3022; c) Z. Wei, X. Wang, A. Borges, M. Santella, T. Li, J. K. Sørensen, M. Vanin, W. Hu, Y. Liu, J. Ulstrup, G. C. Solomon, Q. Chi, T. Bjørnholm, K. Nørgaard, B. W. Laursen, *Langmuir* **2014**, *30*, 14868-14876; d) T. C. Pijper, O. Ivashenko, M. Walko, P. Rudolf, W. R. Browne, B. L. Feringa, *J. Phys. Chem. C* **2015**, *119*, 3648-3657; e) R. J. Davidson, D. C. Milan, O. A. Al-Owaedi, A. K. Ismael, R. J. Nichols, S. J. Higgins, C. J. Lambert, D. S. Yufit, A. Beeby, *RSC Adv.* **2018**, *8*, 23585-23590; f) V. Kolivoška, J. Šebera, T. Sebechlebská, M. Lindner, J. Gasior, G. Mészáros, M. Mayor, M. Valášek, M. Hromadová, *Chem. Commun.* **2019**, *55*, 3351-3354.
[15] S. M. Soria-Castro, A. B. Peñéñory, *Beilstein Journal of Organic Chemistry* **2013**, *9*, 467-475.
[16] C. Wang, A. S. Batsanov, M. R. Bryce, I. Sage, *Synthesis* **2003**, 2089-2095.
[17] D. L. Pearson, J. M. Tour, *J. Org. Chem.* **1997**, *62*, 1376-1387.
[18] G. Sheldrick, *Acta Crystallogr. A* **2008**, *64*, 112-122.
[19] G. Sheldrick, *Acta Crystallogr. A* **2015**, *71*, 3-8.
[20] G. Sheldrick, *Acta Crystallogr. C* **2015**, *71*, 3-8.
[21] O. V. Dolomanov, L. J. Bourhis, R. J. Gildea, J. A. K. Howard, H. Puschmann, *J. Appl. Crystallogr.* **2009**, *42*, 339-341.
[22] a) E. A. Weiss, G. K. Kaufman, J. K. Kriebel, Z. Li, R. Schalek, G. M. Whitesides, *Langmuir* **2007**, *23*, 9686-9694; b) L. T. Banner, A. Richter, E. Pinkhassik, *Surf. Interface Anal.* **2009**, *41*, 49-55.
[23] a) R. Garcia, R. V. Martinez, J. Martinez, *Chem. Soc. Rev.* **2006**, *35*, 29-38; b) N. A. Amro, S. Xu, G. Y. Liu, *Langmuir* **2000**, *16*, 3006-3009; c) M. Kaholek, W. K. Lee, B. LaMattina, K. C. Caster, S. Zauscher, *Nano Lett.* **2004**, *4*, 373-376.
[24] G. Sauerbrey, *Z. Phys.* **1959**, *155*, 206-222.
[25] a) T. P. Weihs, Z. Nawaz, S. P. Jarvis, J. B. Pethica, *Appl. Phys. Lett.* **1991**, *59*, 3536-3538; b) N. A. Burnham, R. J. Colton, H. M. Pollock, *Phys. Rev. Lett.* **1993**, *70*, 247-247; c) E. Gomar-Nadal, G. K. Ramachandran, F. Chen, T. Burgin, C. Rovira, D. B. Amabilino, S. M. Lindsay, *J. Phys. Chem. B* **2004**, *108*, 7213-7218.
[26] N. A. Burnham, R. J. Colton, H. M. Pollock, *Phys. Rev. Lett.* **1992**, *69*, 144-147.
[27] Y. Zhang, X. Qiu, P. Gordiichuk, S. Soni, T. L. Krijger, A. Herrmann, R. C. Chiechi, *J. Phys. Chem. C* **2017**, *121*, 14920-14928.
[28] K. Liu, G. Li, X. Wang, F. Wang, *J. Phys. Chem. C* **2008**, *112*, 4342-4349.
[29] a) M. H. Schoenfisch, J. E. Pemberton, *J. Am. Chem. Soc.* **1998**, *120*, 4502-4513; b) G. Mani, D. M. Johnson, D. Marton, V. L. Dougherty, M. D. Feldman, D. Patel, A. A. Ayon, C. M. Agrawal, *Langmuir* **2008**, *24*, 6774-6784.
[30] J. M. Soler, E. Artacho, J. D. Gale, A. García, J. Junquera, P. Ordejón, D. Sánchez-Portal, *J. Phys.: Condens. Matter* **2002**, *14*, 2745.
[31] J. P. Perdew, A. Zunger, *Phys. Rev. B* **1981**, *23*, 5048-5079.
[32] a) J. Ferrer, C. J. Lambert, V. M. García-Suárez, D. Z. Manrique, D. Visontai, L. Oroszlany, R. Rodríguez-Ferradás, I. Grace, S. W. D. Bailey, K. Gillemot, S. Hatef, L. A. Algharagholy, *New J. Phys.* **2014**, *16*, 093029; b) H. Sadeghi, *Nanotechnology* **2018**, *29*, 373001.
[33] C. J. Lambert, *Chem. Soc. Rev.* **2015**, *44*, 875-888.
[34] J. P. Perdew, K. Burke, M. Ernzerhof, *Phys. Rev. Lett.* **1996**, *77*, 3865-3868.
[35] A. Borges, E.-D. Fung, F. Ng, L. Venkataraman, G. C. Solomon, *J. Phys. Chem. Lett.* **2016**, *7*, 4825-4829.
[36] E. N. Voloshina, D. Mollenhauer, L. Chiappisi, B. Paulus, *Chem. Phys. Lett.* **2011**, *510*, 220-223.
[37] X. Liu, S. Sangtarash, D. Reber, D. Zhang, H. Sadeghi, J. Shi, Z.-Y. Xiao, W. Hong, C. J. Lambert, S.-X. Liu, *Angew. Chem. Int. Ed.* **2017**, *56*, 173-176.

Author Contributions

LJO designed, synthesised and characterised the molecules studied, and led the drafting of the manuscript.

XW conducted the microscopy, reductive desorption and QCM studies and contributed to the drafting of the manuscript.

MJ conducted computational studies and contributed to the drafting of the manuscript.

ASB solved the X-ray crystal structures.

HS supervised and contributed to the computational studies.

CJL, BJR and MRB were responsible for supervision and project administration, and acquisition of project funding.
